# Global and regional prevalence of osteopenia in chronic kidney disease: a systematic review and meta-analysis

**DOI:** 10.1007/s10238-025-01909-3

**Published:** 2025-11-11

**Authors:** Mobin Ghazaiean, Iradj Maleki, Tahoora Mousavi, Behnam Najafi, Mahmood Moosazadeh

**Affiliations:** 1 Gut and Liver Research Center, Non-Communicable Disease Institute, Mazandaran University of Medical Sciences, Sari, Iran; 2 Gut and Liver Research Center, Non-Communicable Disease Institute, Mazandaran University of Medical Sciences, Sari, Iran; 3 Molecular and Cell Biology Research Center, Hemoglobinopathy Institute, Mazandaran University of Medical Sciences, Sari, Iran; 4 Student Research Committee, School of Medicine, Mazandaran University of Medical Sciences, Sari, Iran; 5 Gastrointestinal Cancer Research Center, Non-Communicable Diseases Institute, Mazandaran University of Medical Sciences, Sari, Iran

**Keywords:** Chronic kidney disease, Low bone density, Osteopenia, Adults, Epidemiology, Prevalence

## Abstract

The global prevalence of osteopenia among people with chronic kidney disease (CKD) remains uncertain. This research aimed to estimate the global and regional prevalence of osteopenia in CKD patients. Databases including PubMed, Scopus, Science Direct, Web of Science, and Embase, along with Google Scholar engine, were explored from January 1, 2000, to January 1, 2025, in English. The eligibility criteria included adults (≥ 18 years) with CKD stages 3a-5D. The risk of bias assessment performed using the Joanna Briggs Institute (JBI) checklist. The I-square index was employed to assess heterogeneity, whereas publication bias was evaluated through Egger’s test and funnel plots. Statistical analyses were performed using Stata software Version 17. The prevalence of femoral neck osteopenia in CKDs estimated 47% (95% CI: 43 to 50, *n* = 45). The lumbar spine osteopenia estimated 35% (95% CI: 33 to 37, *n* = 52). Based on specific bone site, the osteopenia prevalence is 40% (95% CI: 35 to 45, *n* = 24) in total hip, 28% (95% CI: 19 to 38, *n* = 5) in forearm, and 31% (95% CI: 24 to 37, *n* = 9) in distal radius. The sex-specific rate is 37% (95% CI: 32 to 42, *n* = 30) for men and 36% (95% CI: 31 to 41, *n* = 30) for women. Our study uncovered a significant prevalence of osteopenia in CKD patients stages 3a-5D, with the highest rate in bone areas abundant in cortical content (femoral neck region).

**Supplementary Information:**

The online version contains supplementary material available at 10.1007/s10238-025-01909-3.

## Introduction

Chronic kidney disease (CKD) is a prevalent condition that has emerged as a significant concern for public health [1]. CKD is estimated to affect around 8–16% of the global population [2]. Around 772 million individuals globally have been diagnosed with CKD [3]; in the USA, more than 20 million people are impacted [3]. The CKD stages can be classified based on the glomerular filtration rate (GFR), from G1 to G5. G1 encompassed normal GFR (over 90 mL/min/1.73 m^2^) while G2 consisted of a mild reduction in GFR (60–89 mL/min/1.73 m^2^). A mild to moderate decrease in GFR is classified as CKD stage 3a (40–59 mL/min/1.73 m^2^), while a moderate to severe decrease is categorized as CKD stage 3b (30–44 mL/min/1.73 m^2^). The significant impairment is classified as CKD stage 4 (15–29 mL/min/1.73 m^2^) and kidney failure or CKD stage 5 (below 15 mL/min/1.73 m^2^) [4]. The repercussions associated with CKD included a decline in kidney function, progressing to end-stage renal disease (ESRD), and impacting the function of other organs. Renal replacement therapy for patients with ESRD includes dialysis modalities like hemodialysis or peritoneal dialysis, as well as kidney transplantation [5].

Bone density loss is typically evaluated in clinical settings using dual-energy X-ray absorptiometry (DEXA) under most circumstances [6]. This technique is employed for various reasons, including identifying BMD loss, assessing fracture risk, monitoring density changes over time, and evaluating treatment results [7]. Utilizing suitable reference data, reporting DEXA measurements at the lumbar spine, femoral neck, total hip, and/or “one-third radius” is used for diagnosing osteoporosis (lowest T-score ≤ −2.5), considering the lowest T-score from any site [8, 9]. Osteopenia or reduced bone density (−2.5 < lowest T-score < −1) threshold has been proposed for osteoporosis prevention. This category encompassed patients with varying bone mineral density, including low-normal BMD and low BMD [10]. The significance of identifying bone density loss stems from the reality that osteopenia and osteoporosis lack symptoms until fractures take place due to the severity of the disease, which is associated with ongoing health issues. The initiatives aimed at preventing bone fractures are highly significant due to their increasing global influence resulting from an aging population [11, 12].

As CKD advances, the decrease in BMD primarily affects the hip area rather than the lumbar spine [13]. Researchers have recognized the link between CKD and its effect on BMD. Impaired kidney function influences the quality of bone tissue, leading to metabolic disturbances in bone and minerals, which are classified as a systemic condition referred to as chronic kidney disease–mineral bone disorder (CKD-MBD). CKD-MBD may manifest as one or a combination of disorders, including abnormalities in parathyroid hormone (PTH), phosphorus, calcium, vitamin D metabolism, and bone issues like volume, strength, linear growth, mineralization, and turnover, along with soft tissue calcification like vascular calcification [14]. The clinical significance of BMD assessment in CKD patients pertains to the bone deterioration and associated health issues arising from diverse changes in hormonal and metabolic processes [15, 16]. Patients with ESRD frequently exhibit mineral and bone disorders, which are linked to a higher risk of fractures, soft tissue calcification, including vascular calcification, cardiovascular events, and mortality associated with cardiovascular events [17, 18].

Compared to individuals without CKD, those with CKD have more than a 2.5-fold higher risk of fractures, whereas individuals on dialysis experience a risk that surpasses fourfold [19]. A systematic review and meta-analysis released in 2023 found that the prevalence of low bone mineral density (T-score ≤ -2.5) in adults with CKD stages 3a-5D was 24.5% (95% CI: 21.3 to 27.8), with estimated prevalence in dialysis and non-dialysis patients being 30% (95% CI: 25 to 35) and 12% (95% CI: 8 to 16), respectively [20]. In the National Health and Nutrition Examination Survey (NHANES III), participants with eGFR < 60 mL/min had a higher osteoporosis rate, being twice as common when compared to those with eGFR > 60 mL/min [21]. A 2020 meta-analysis assessing the associations among CKD, falls, and fractures revealed that the risk of fractures increased as kidney function worsened, with the highest risks identified in patients with CKD stage 5 or undergoing dialysis. A significant association was identified between fractures and eGFR categories of 45–59, 30–44, 15–29, and under 15. In the eGFR category of 45–59, a marginal correlation was detected with a pooled HR of 1.36 (95%CI: 0.99 to 1.86). The risk escalated in later stages of CKD, with the highest risk noted in patients with an eGFR < 15, pooled HR 2.63 (95%CI: 1.74 to 3.98). This impact was most apparent for hip fractures and all types of fractures [22]. Earlier research showed that those with osteoporosis have a heightened fracture risk; however, adults with osteopenia have a higher fracture rate due to the larger number of osteopenia cases [23]. Osteopenia encompasses individuals who face both low and very high chances of sustaining fractures. Osteopenia offers limited diagnostic significance, but improving bone health in older adults with it is vital as most fractures occur in this group [24]. Therefore, osteopenia, as the early stage of osteoporosis, is a critical phase for preventing osteoporosis and fractures.

Lately, numerous independent studies have demonstrated the prevalence of osteopenia in specific regions. The prevalence of osteopenia varied notably among different studies and regions. To our understanding, there is presently no thorough and detailed summary of the evidence concerning the prevalence of osteopenia in CKDs. This information is crucial for guiding healthcare planning and policy-making, offering understanding of existing trends and future forecasts, and facilitating the development of long-term epidemiological strategies and required treatment resources for those with osteopenia, thus alleviating the serious risks of fractures and lowering related mortality rates. Due to the considerable clinical, economic, and social consequences of osteoporosis, precise prevalence estimates of osteopenia are essential for guiding policy choices. These choices influence how individuals in need of treatment are identified and their ability to access drug therapies and continuous monitoring for fracture risk reduction.

## Methods

### Search strategy and selection criteria

Leveraging the existing data, our research estimated the global and regional prevalence of osteopenia among patients with CKD. This research does not require an ethical declaration because it is based on a systematic review and meta-analysis of existing literature. The research methods used in this study were conducted in accordance with the 2020 PRISMA (Preferred Reporting Items for Systematic Reviews and Meta-Analyses) guidelines [25]. This review has been recorded with PROSPERO under the code CRD420251007415.

### Systematic search

We conducted a systematic literature review to assess the prevalence of osteopenia in adults suffering from CKD. We subsequently performed a meta-analysis to evaluate the prevalence of osteopenia and to illustrate the prevalence of the condition across different status of CKD and skeletal regions. We conducted thorough electronic searches across various databases including PubMed, Scopus, Web of Science, Embase, and Science Direct, along with the Google Scholar engine, to identify published studies investigating the prevalence of osteopenia in CKD patients from January 1, 2000, to January 1, 2025, in English.

### Search strategy

The search terms were carefully selected from the Medical Subject Headings (MeSH) database, literature reviews, and other relevant index terms. The search terms included “chronic kidney disease,” “chronic renal disease,” “chronic kidney insufficiency,” “chronic renal insufficiency,” “kidney disease,” “renal disease,” “kidney insufficiency,” “renal insufficiency,” “kidney failure,” “renal failure,” “non-dialysis,” “predialysis,” “pre-dialysis,” “hemodialysis,” “haemodialysis,” “hemofiltration,” “haemofiltration,” “hemodiafiltration,” “haemodiafiltration,” “peritoneal dialysis,” “osteopenia,” “bone loss,” “bone health,” “bone density,” and “bone mineral density,” with all possible combinations tailored to the specific patterns of each database. Additionally, the search was enhanced by carefully reviewing the reference lists of the identified articles to ensure completeness. Additional details regarding the study search can be found in the supplementary file.

### Inclusion criteria

The study’s inclusion criteria encompassed all research focused on osteopenia in individuals aged 18 and older with CKD in stages 3a-5D (i.e., eGFR < 60 ml/min per 1.73 m^2^), determined by assessing BMD via DEXA, following definitions by the International Society for Clinical Densitometry (ISCD, defined as T-score between −1 and −2.5) [26, 27], from January 1, 2000, through January 1, 2025. This included observational studies such as cross-sectional, case–control, cohort studies, and studies detailing the initial data of osteopenia in CKDs. The prevalence rate will be showcased as lumbar, femoral neck, total hip, forearm, distal radius, and general osteopenia (a general category of osteopenia that includes studies not specifying osteopenia rates by particular skeletal areas). Fifteen articles were not included in the screening process due to their lack of availability [28–42].

### Exclusion criteria

The exclusion criteria for this research included duplicate studies, research not relevant to the study’s topic and goals, studies with unclear methodology, interventional research lacking baseline osteopenia data, as well as experimental and qualitative studies, case reports, and studies not published in English. Additionally, we omitted conference abstracts, protocols, books/book chapters, preprints, reviews (whether narrative or systematic), as well as letters, news articles, opinions, and commentaries. Studies that were excluded during the full-text screening stage are briefly summarized with explanations in the supplementary file, eTable 1.

### Selection process

By using reference management software, EndNote X7 (Version 17), duplicate entries eliminated. The appropriateness of the studies was assessed by assessing their titles and abstracts. Afterward, two researchers, M.G. and M.M., independently evaluated the full texts. Any inconsistencies were resolved with the participation of the third author, T.M.

### Data collection process

Two researchers (M.G. and I.M.) collected data independently utilizing a structured data collection form. Any differences among researchers were resolved, and an agreement was achieved through conversations with the third author (M.M.). The following details were gathered from each study: author name, year of publication, location, sample size, age (mean ± SD data for osteopenic and normal BMD patients), sex, diagnostic criteria for osteopenia, HDI Tier, CKD status (including non-dialysis (ND) and dialysis such as hemodialysis (HD), peritoneal dialysis (PD), and hemodialysis plus peritoneal dialysis (HD + PD)), total number of osteopenia across various skeletal sites such as lumbar, femoral neck, total hip, forearm, and distal radius, along with the number of general osteopenia cases.

### Definition

HDI Tier: This study additionally evaluated the prevalence of CKDs, informed by the HDI ratings of nations in 2024. The HDI, created by the United Nations Development Programme (UNDP) in 1990 and published yearly, assesses human development by emphasizing "expanding individuals’ options." It includes the fundamental components of health, education, and income, which serve as the basis for these choices. A nation’s HDI score is determined by taking into account various indicators, including life expectancy, literacy levels, access to electricity in rural regions, Gross Domestic Product (GDP) per capita, trade data, homicide rates, the multi-dimensional poverty index, income disparity, and internet availability. These metrics are merged to form one value ranging from 0 to 1.0, where 1.0 denotes the peak level of human development [43, 44]. The HDI is divided into four categories: very high (0.8–1.0), high (0.7–0.79), medium (0.55–0.70), and low (below 0.55) (https://worldpopulationreview.com/country-rankings/hdi-by-country).

### Risk of bias assessment

In this research, two investigators (M.G. and I.M.) conducted the risk of bias (ROB) assessment separately, with any discrepancies resolved by the supervisor (M.M.) when necessary. The quality of the articles was assessed using the JBI Critical Appraisal Checklist [45]. The JBI tool comprises nine items, with each providing four potential responses: yes, no, unclear, or not applicable. A higher number of "yes" replies indicate enhanced quality of the study. Additional details about the methodological assessment can be found in the supplementary file, eTable 2.

#### Data analysis

The analysis of the data employed Stata version 17 software to determine the prevalence of osteopenia in CKDs, using the binomial distribution formula. Heterogeneity was assessed visually via forest plots. The Cochran’s Q test (employing a significance level of 0.05) and I^2^ statistic (with I^2^ values of ≥ 50% denoting substantial heterogeneity) were used to assess the significance of statistical heterogeneity [46]. The evaluation of publication bias was performed utilizing a funnel plot in conjunction with Egger’s test. The Trim and Fill method was utilized to assess publication bias. A random effects model was employed to assess the prevalence of osteopenia in CKDs. The forest plot depicts the prevalence estimates along with their 95% confidence intervals. Subgroup analyses performed based on varying skeletal locations, gender, status of CKD, continent, and HDI category. Furthermore, the meta-regression approach was employed to determine the source of heterogeneity, including continent, HDI category, and CKD status (HD, PD, HD + PD, and ND). In addition, a sensitivity analysis was conducted to assess the impact of each primary study on the overall estimate.

Moreover, the study incorporated findings that outlined the mean and standard deviation for the quantitative variable, age, among CKD adults with osteopenia (case group) in contrast with CKDs with normal BMD (control group). The BMD was classified as osteopenia (T-score between −1.0 and −2.5) based on the T-score classifications [26, 27]. For the studies incorporated that offered median and interquartile range or median and range, we applied the techniques described by Luo et al. and Wan et al. [47, 48] to calculate the mean and standard deviation of the data. Employing Methane’s equation, a random effects model, and Cohen’s d coefficient, the SMD was computed, along with a 95% confidence interval. We evaluated the SMD, participant number, means, and standard deviations of the quantitative data for each case and control group separately. The benchmarks for assessing the significance of the SMD between two groups (case and control) state that zero must be absent from the upper and lower confidence intervals of the SMD [49].

## Result

### Study selection

**Fig. 1:**
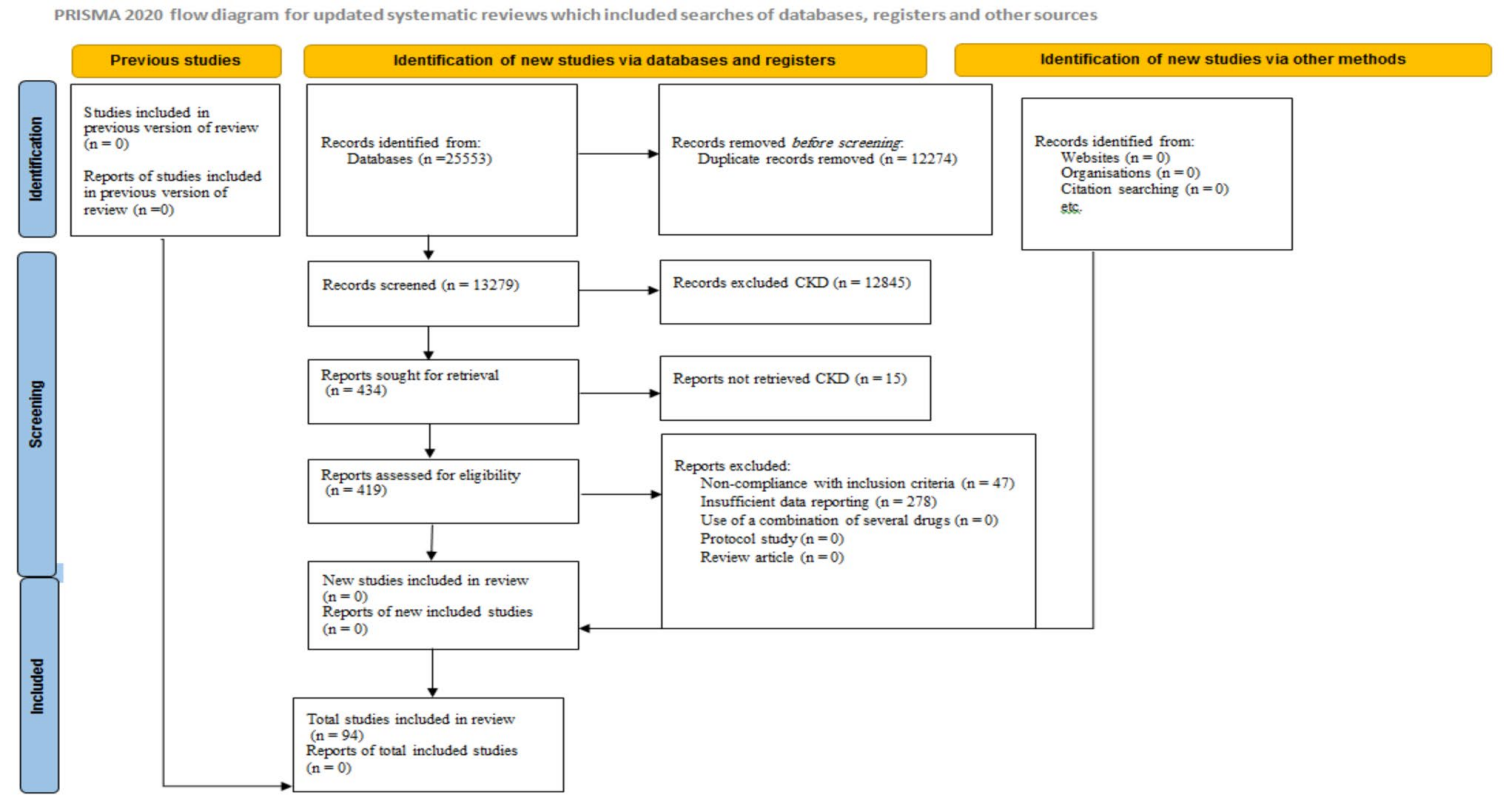
Flow diagram of included/excluded studies

By performing extensive searches in multiple databases with appropriate keywords, we first discovered 25,553 primary studies. Duplicate articles were first identified and eliminated with End Note software (*n* = 12,274). Following this, the titles and abstracts of the entries were reviewed to remove unrelated material (*n* = 12,845). The next step involved excluding reports that were not obtained (*n* = 15). Furthermore, we eliminated 325 studies for different reasons, which included 47 studies for not meeting inclusion criteria and 278 studies for inadequate data reporting. A list of studies that were excluded along with brief reasons can be found in the supplementary file, eTable 1. By employing a thorough screening procedure and applying particular inclusion and exclusion criteria, 94 primary studies were ultimately selected. The PRISMA diagram (Fig. 1) visually illustrates the selection process for the included studies.

### Study characteristics

The research included 94 studies conducted in 27 nations and across five continents. The distribution of studies by continent is as follows: 53 in Asia, 22 in Europe, 10 in America, 3 in Africa, 5 in Australia, and one multi-national. These investigations included 16,033 individuals with CKD, consisting of 6,348 men and 7,046 women. The study designs included 68 cross-sectional studies, 22 cohort studies, 2 case–control studies, and 2 randomized controlled trials. In addition, 30 studies provided data about the gender of the osteopenic individuals. Additionally, 18 studies offered details concerning the age of osteopenic patients compared to those with normal BMD. According the HDI Tier, 58 studies were assigned to “Very High” category, 27 studies were categorized in the “High” category, and 9 studies were identified in the “Medium” category. None of the studies referenced were conducted in countries with a Low HDI. The characteristics of the studies that were incorporated can be found in the supplementary file, eTables 3 and 4.

### Bone site prevalence

#### Prevalence of osteopenia in the femoral neck region

**Fig. 2:**
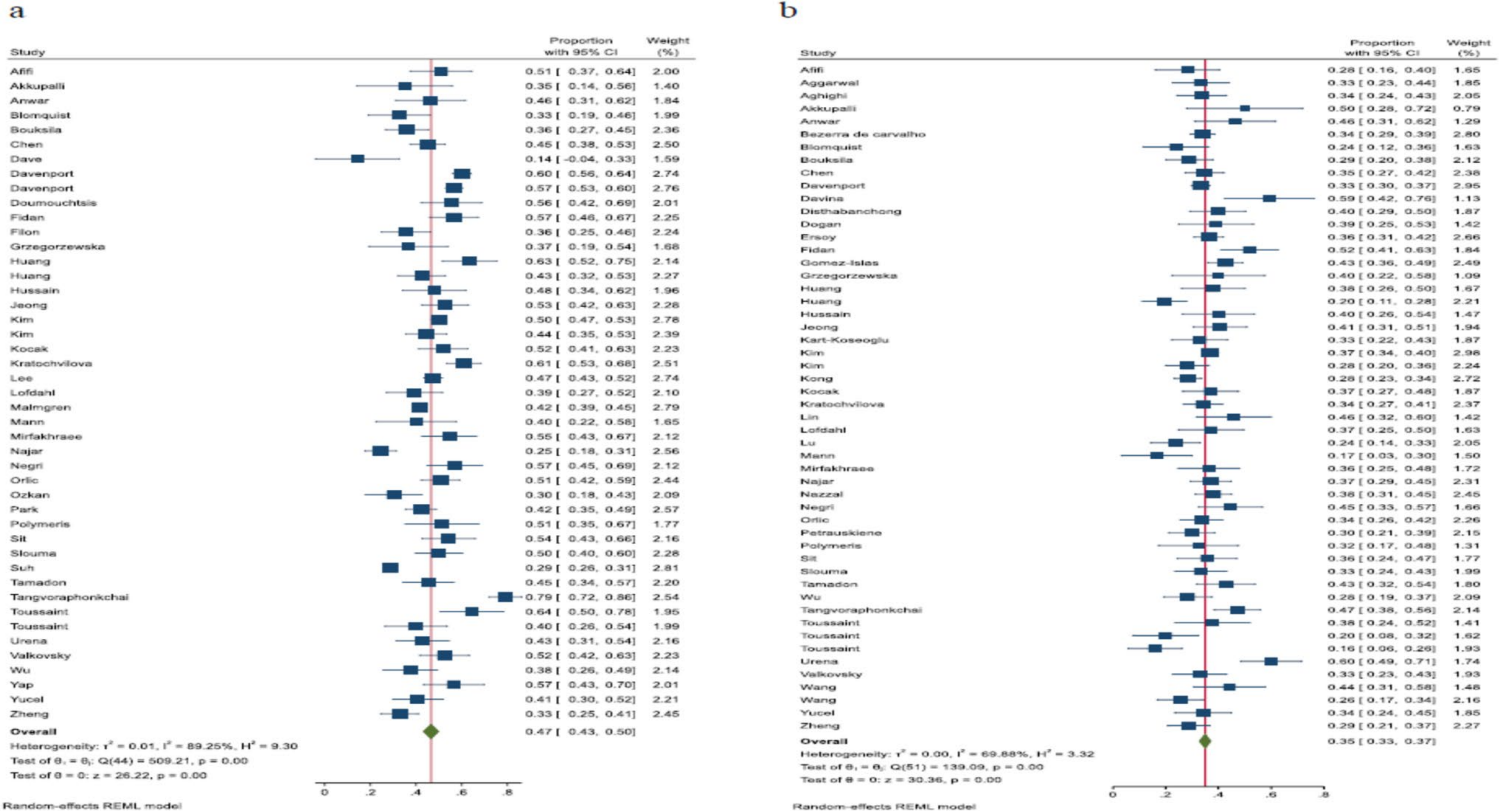
Forest plot of the prevalence of osteopenia by the primary studies in adults with chronic kidney disease and the overall estimate (95% CI). a: Femoral neck region. b: Lumbar spine region

Osteopenia in the femoral neck has been documented in 45 studies. Among these, there are 13 studies from Europe, 21 from Asia, 4 from America, 3 from Africa, and 4 from Australia. Among these, 32 studies pertain to nations with a very high HDI, 9 studies concern countries with a high HDI, and 4 studies with medium HDI. The prevalence of osteopenia in the femoral neck showed significant variation, with estimates from 14% in Dave’s et al. to 79% in Tangvoraphonkchai et al.’s research. Heterogeneity indices reveal a significant degree of variation among the outcomes of primary studies (I-squared: 89.25%, Q: 509.21, *P* < 0.001). By integrating the results from 45 studies, the estimated prevalence of osteopenia in the femoral neck is found to be 47% (95% CI: 43 to 50) (Table 1 and Fig. 2a).

**Table 1:** Summary of meta-analysis and subgroups analysis results of the osteopenia prevalence among adults with CKD

Characteristics	No. of studies/ Total	Prevalence rate		Heterogeneity		
		ES (95%CI)	Model	Chi square	P-value	I-square (%)
*Sex-based prevalence*						
Male	30/94	37% (32–42)	Random	278.37	< 0.001	88.54
Female	30/94	36% (31–41)	Random	185.66	< 0.001	84.04
*Bone site prevalence*						
Lumbar osteopenia	52/94	35% (33–37)	Random	139.09	< 0.001	69.88
Femoral neck osteopenia	45/94	47% (43–50)	Random	509.21	< 0.001	89.25
Total hip osteopenia	24/94	40% (35–45)	Random	237.7	< 0.001	90.32
Forearm osteopenia	5/94	28% (19–38)	Random	16.41	< 0.001	69.95
Distal radius osteopenia	9/94	31% (24–37)	Random	35.3	< 0.001	78.28
General osteopenia	29/94	40% (34–45)	Random	452.05	< 0.001	93.83
*Femoral neck osteopenia subgroups (Sex-based prevalence)*						
Male	17/45	49% (43–55)	Random	87.78	< 0.001	79.63
Female	16/45	45% (38–52)	Random	69.75	< 0.001	80.11
*Femoral neck osteopenia subgroups (CKD status-based prevalence)*						
HD	24/45	46% (42–50)	Random	64.73	< 0.001	65.36
PD	7/45	55% (43–66)	Random	59.73	< 0.001	94.53
HD + PD	4/45	49% (38–59)	Random	15.78	< 0.001	78.69
ND	8/45	40% (33–48)	Random	144.5	< 0.001	94.61
*Femoral neck osteopenia subgroups (Continent-based prevalence)*						
Europe	13/45	52% (45–58)	Random	152.07	< 0.001	92.18
Asia	21/45	44% (39–48)	Random	204.28	< 0.001	86.07
America	4/45	47% (35–58)	Random	9.01	< 0.001	66.56
Africa	3/45	45% (35–55)	Random	5.09	< 0.001	59.78
Australia	4/45	44% (23–66)	Random	21.21	< 0.001	88.01
*Femoral neck osteopenia subgroups (HDI-based prevalence)*						
Very high	32/45	48% (44–52)	Random	447.89	< 0.001	91.37
High	9/45	44% (38–50)	Random	23.80	< 0.001	67.28
Medium	4/45	38% (25–50)	Random	13.18	< 0.001	71.70
*Lumbar osteopenia subgroups (Sex-based prevalence)*						
Male	16/52	33% (28–38)	Random	48.69	< 0.001	72.47
Female	14/52	35% (30–39)	Random	24.88	< 0.001	49.04
*Lumbar osteopenia subgroups (CKD status-based prevalence)*						
HD	27/52	34% (31–37)	Random	50.50	< 0.001	50.18
PD	9/52	38% (33–44)	Random	23.14	< 0.001	70.15
HD + PD	4/52	37% (32–41)	Random	0.77	< 0.001	0
ND	11/52	36% (30–42)	Random	48.08	< 0.001	86.27
*Lumbar osteopenia subgroups (Continent-based prevalence)*						
Europe	10/52	38% (32–43)	Random	28.74	< 0.001	74.58
Asia	29/52	36% (33–38)	Random	69.94	< 0.001	63.62
America	6/52	34% (26–42)	Random	18.25	< 0.001	78.75
Africa	3/52	30% (25–36)	Random	0.56	< 0.001	0.01
Australia	3/52	24% (11–37)	Random	6.19	< 0.001	69.08
*Lumbar osteopenia subgroups (HDI-based prevalence)*						
Very high	28/52	35% (32–39)	Random	77.11	< 0.001	74.28
High	18/52	33% (30–36)	Random	43.51	< 0.001	62.42
Medium	6/52	42% (35–49)	Random	8.49	< 0.001	42.55
*Total hip osteopenia subgroups (CKD status-based prevalence)*						
HD	11/24	44% (40–49)	Random	33.63	< 0.001	61.07
HD + PD	4/24	42% (29–56)	Random	24.22	< 0.001	88.88
ND	7/24	35% (24–46)	Random	126.50	< 0.001	95.41

CKD, Chronic kidney disease; HD, Hemodialysis; PD, Peritoneal dialysis; ND, Non-dialysis; HDI, Human development index

Based on sex-specific rates in individuals with femoral neck osteopenia, the prevalence of osteopenia in male and female CKD patients has been extensively studied. The prevalence of osteopenia in males has exhibited notable differences, varying from 26% in Ozkan’s research to 69% in Huang’s research. Heterogeneity indices (I-squared: 79.63%, Q: 87.78, *P* < 0.001) suggest significant heterogeneity in the outcomes of these primary studies. By aggregating the results of these 17 studies, the estimated prevalence of osteopenia in male patients with CKD is 49% (95% CI: 43 to 55). In females, the prevalence of osteopenia has demonstrated considerable variation, ranging from 17% in Najar’s research to 66% in Kratochvilova’s study. Heterogeneity metrics (I-squared: 80.11%, Q: 69.75, *P* < 0.001) show significant heterogeneity within the findings of these primary studies. When aggregating the results from these 16 studies, the estimated prevalence of osteopenia in women with CKDs is 45% (95% CI: 38 to 52).

In relation to the CKD status, there were 24 studies performed on HD patients, 7 on PD patients, 4 on those with both HD and PD, 8 on ND patients, one study was mixture of dialysis and non-dialysis patients, and one study was undefined. The prevalence of femoral neck osteopenia was 46% (95% CI: 42 to 50) in HD, 55% (95% CI: 43 to 66) in PD, 49% (95% CI: 38 to 59) in studies with combination of HD and PD patients, and 40% (95% CI: 33 to 48) in ND patients.

According to subgroup analysis by continent, the prevalence of femoral neck osteopenia was 44% (95% CI: 39 to 48, *n* = 21) in Asia, 52% (95% CI: 45 to 58, *n* = 13) in Europe, 47% (95% CI: 35 to 58, *n* = 4) in America, 45% (95% CI: 35 to 55, *n* = 3) in Africa, and 44% (95% CI: 23 to 66, *n* = 4) in Australia (Table 1). In nations with very high HDI, the prevalence is 48% (95% CI: 44 to 52, *n* = 32), whereas in nations with high HDI, it is estimated to be 44% (95% CI: 38 to 50, *n* = 9). The prevalence indicated in nations with medium HDI was estimated at 38% (95% CI: 25 to 50, *n* = 4) (Table 1). Additionally, publication bias was assessed by the funnel plot (eFigure 1) and Egger’s test (*β* = -0.73, *P* = 0.39). In order to tackle this bias, a trim and fill analysis was conducted. The findings from this analysis indicate that a new study has not been included, and the prevalence of osteopenia is still the same. The meta-regression analysis, designed to investigate elements causing heterogeneity, indicated that the continent (*β* = 0.03, *P* = 0.09), the HDI level (*β* = −0.05, P = 0.09), and CKD status (*β* = −0.01, *P* = 0.37) were not related to heterogeneity. It is important to emphasize that, based on the findings of the sensitivity analysis, the influence of each individual study on the overall estimate was not significant. Table 1 provides a detailed account of the meta-analysis findings and subgroup analysis for the femoral neck region.

#### Prevalence of osteopenia in the lumbar region

The prevalence of osteopenia in the lumbar region has been evaluated in 52 studies. Among these, there are 10 studies conducted in Europe, 29 in Asia, 6 in America, 3 in Africa, 3 in Australia, and 1 multi-national study. Among these, 28 studies pertain to nations with very high HDI, 18 studies concern countries with high HDI, and 6 studies are associated with medium HDI. The prevalence of osteopenia in the lumbar region showed significant variation, ranging from 16% in Toussaint’s study to 60% in Urena et al.’s research. Heterogeneity indices show a significant degree of variation among the findings of primary studies (I-squared: 69.88%, Q: 139.09, *P* < 0.001). By integrating the results from the 52 studies, the projected prevalence of osteopenia in the lumbar region is 35% (95% CI: 33 to 37) (Table 1 and Fig. 2b). The prevalence of osteopenia in male and female CKD patients has been extensively studied based on sex-specific rates in individuals with lumbar osteopenia. The prevalence of osteopenia in males has exhibited significant differences, varying from 10% in Lu’s research to 59% in Davina’s research. The heterogeneity indices (I-squared: 72.47%, Q: 48.69, *P* < 0.001) demonstrate a significant level of heterogeneity across the findings of these primary studies. After aggregating the results of these 16 studies, the estimated prevalence of osteopenia in male CKDs is 33% (95% CI: 28 to 38). In females, the prevalence of osteopenia has exhibited significant variation, varying from 18% in Kim’s research to 61% in Anwar’s research. The heterogeneity indices (I-squared: 49.04%, Q: 24.88, *P* < 0.001) suggest a significant degree of variability among the findings of these primary studies. By aggregating the results of these 14 studies, the projected prevalence of osteopenia in females with CKDs is 35% (95% CI: 30 to 39) (Table 1).

According to the CKD status, 27 studies involved HD patients, 9 focused on PD patients, 4 included both HD and PD patients, 11 centered on ND patients, and 1 study assessed HD, PD, and non-dialysis patients. The prevalence of lumbar osteopenia was 34% (95% CI: 31 to 37) among HD patients, then 38% (95% CI: 33 to 44) in PD patients, 37% (95% CI: 32 to 41) in studies with combination of HD and PD patients, and 36% (95% CI: 30 to 42) in ND patients.

According to subgroup analysis categorized by continent, the prevalence of lumbar osteopenia was 36% (95% CI: 33 to 38, *n* = 29) in Asia, 38% (95% CI: 32 to 43, *n* = 10) in Europe, 34% (95% CI: 26 to 42, *n* = 6) in America, 30% (95% CI: 25 to 36, *n* = 3) in Africa, and 24% (95% CI: 11 to 37, *n* = 3) in Australia (Table 1). In countries with very high HDI, the prevalence is 35% (95% CI: 32 to 39, *n* = 28), while in those with high HDI, it is estimated to be 33% (95% CI: 30 to 36, *n* = 18). The estimated prevalence in nations with medium HDI was reported to be 42% (95% CI: 35 to 49, n = 6) (Table 1). Additionally, publication bias was assessed by the funnel plot (eFigure 2) and Egger’s test (*β* = 1.28, *P* = 0.06). In order to tackle this bias, a trim and fill analysis was conducted. The findings of this analysis indicate that a new study has not been included and the rate of osteopenia stays the same. The meta-regression analysis, designed to investigate factors linked to heterogeneity, indicated that the continent (*β* = 0.01, *P* = 0.45), the HDI level (*β* = 0.01, *P* = 0.53), and CKD status (*β* = 0.01, *P* = 0.57) showed no association with heterogeneity. It is important to emphasize that, based on the outcomes of the sensitivity analysis, the effect of each main study on the overall estimate was not significant. The findings of the meta-analysis and the subgroup analysis concerning the lumbar region are outlined in Table 1.

#### Prevalence of osteopenia in the total hip region

In 24 studies, researchers have investigated the prevalence of osteopenia in the total hip area. Of these, 7 studies originate from Europe, 10 from Asia, 6 from America, and 1 from Africa. Among these studies, 16 were carried out in nations with very high HDI, 7 in nations with high HDI, and 1 in a nation with medium HDI. The prevalence of osteopenia in the total hip showed significant variation, between 8% in Chue’s research and 60% in the study by Kratochvilova et al. Heterogeneity indices (I-squared: 90.32%, Q: 237.7, *P* < 0.001) demonstrated significant heterogeneity within the primary studies. By integrating the results from these 24 studies, the projected prevalence of osteopenia in the total hip area is 40% (95% CI: 35 to 45) (Table 1 and eFigure 3).

According to the CKD status, there were 11 studies performed on HD patients, 1 on PD patients, 4 on HD + PD patients, 7 on ND patients, and 1 with an undefined CKD status. The prevalence of total hip osteopenia was 44% (95% CI: 40 to 49) among HD patients, 23% (95% CI: 8 to 38) in PD patients, 42% (95% CI: 29 to 56) in studies with combination of HD and PD patients, and 35% (95% CI: 24 to 46) in ND patients.

The prevalence of osteopenia in the total hip is 39% (95% CI: 32 to 45, *n* = 16) in nations with very high HDI and 41% (95% CI: 37 to 46, *n* = 7) in nations with high HDI (Table 1). Publication bias was assessed by the funnel plot (eFigure 4) and Egger’s test (*β* = 1.39, *P* = 0.27). A trim and fill analysis was performed to tackle this bias. According to the study’s results, a new study has not been conducted, and the prevalence of osteopenia in the total hip area remains the same. The meta-regression analysis indicated that the continent (*β* = -0.01, *P* = 0.7), HDI level (*β* = 0.05, *P* = 0.25), and CKD status (*β* = −0.03, *P* = 0.1) showed no relationship with heterogeneity. Findings from the sensitivity analysis suggest that the impact of each main study on the overall estimate was not significant. The findings of the meta-analysis and subgroup analysis for the total hip area are presented in Table 1.

#### Prevalence of osteopenia in the forearm region

Five studies have investigated the prevalence of osteopenia in the forearm area. The heterogeneity indices reveal a significant degree of variability among the outcomes of the primary studies (I-squared: 69.95%, Q: 16.41, *P* < 0.001). Merging the findings from these studies, the projected prevalence of osteopenia in the forearm is 28% (95% CI: 19 to 38). Publication bias was evaluated through Begg’s test, yielding a non-significant result (*P* = 0.81). To counteract this bias, a trim and fill assessment was performed. According to the study’s findings, a new study has not been conducted, and the prevalence of osteopenia in the forearm area stays the same. Moreover, the findings from the sensitivity analysis concerning the influence of the individual studies on the overall estimate were not significant. The results of the meta-analysis for the forearm area are shown in Table 1.

#### Prevalence of osteopenia in the distal radius region

Osteopenia in the distal radius has been recorded in nine studies. The indices of heterogeneity (I-squared: 78.28%, Q: 35.3, *P* < 0.001) reveal significant variability among the results of the primary studies. Evaluation of these studies suggests that the prevalence of osteopenia in the distal radius area is 31% (95% CI: 24 to 37) (Table 1). Publication bias was evaluated through Begg’s test, which showed no significant result (*P* = 0.25). To tackle this bias, a trim and fill evaluation was performed. According to the study’s results, a new study has not been conducted, and the prevalence of osteopenia in the distal radius area remains the same. The sensitivity analysis indicated that none of the primary studies had a significant impact on the overall estimate. The findings of the meta-analysis for the distal radius area are shown in Table 1.

#### Prevalence of general osteopenia

In 29 studies, the prevalence of general osteopenia was documented. Of these, 18 studies originated in Asia, 5 in Europe, 3 in America, and 3 in Africa. The prevalence of general osteopenia in CKDs varied from 7% in Shin’s study to 69% in the research by Jamal et al. The heterogeneity indices (I-squared: 93.83%, Q: 452.05, *P* < 0.001) suggested a significant level of heterogeneity in the findings of the primary studies. The aggregate findings of 29 studies suggest that the prevalence of general osteopenia in CKDs is 40% (95% CI: 34 to 45) (eFigure 5). Moreover, publication bias was assessed by the funnel plot diagram (eFigure 6) and Egger’s test (*β* = 1.20, *P* = 0.36). A trim and fill analysis was performed to tackle this bias. According to this test, the trim and fill analysis indicated that the outcome remains the same. Additionally, the sensitivity analysis results concerning the influence of individual studies on the overall estimate were not significant.

### Gender-specific prevalence

#### Prevalence of osteopenia in male patients

In 30 studies, the prevalence of osteopenia in male CKD patients has been extensively analyzed. The prevalence of osteopenia in men has displayed considerable fluctuation, varying from 3% in Shin’s research to 61% in Lee’s research. Heterogeneity indices (I-squared: 88.54%, Q: 278.37, *P* < 0.001) reveal significant heterogeneity in the findings of these primary studies. By integrating the results of these 30 studies, the projected prevalence of osteopenia in male CKD patients is 37% (95% CI: 32 to 42) (eFigure 7). Publication bias was assessed by the funnel plot (eFigure 8) and Egger’s test (*β* = 1.27, *P* = 0.28). A trim and fill analysis was performed to tackle this bias. According to the results of this analysis, the outcome remains the same. The findings of the sensitivity analysis showed that the impact of each main study on the total estimate was not significant.

#### Prevalence of osteopenia in female patients

The prevalence of osteopenia in female CKD patients has been assessed in 30 studies. The osteopenia prevalence among women has demonstrated notable differences, varying from 8% in Shin’s research to 61% in Anwar’s research. The heterogeneity indices (I-squared: 84.04%, Q: 185.66, *P* < 0.001) demonstrate significant variability in the outcomes of the primary studies. Merging the results from these 30 studies, it is estimated that the prevalence of osteopenia in women with CKDs is 36% (95% CI: 31 to 41) (Table 1 and eFigure 9). Publication bias was assessed by the funnel plot (eFigure 10) and Egger’s test (*β* = 1.03, *P* = 0.25). A trim and fill analysis was performed to tackle this bias. According to this analysis, the prevalence of osteopenia in women with CKDs remains unchanged. It is important to note that the sensitivity analysis revealed that the effect of each main study on the overall estimate was not significant.

### Standardized mean difference of the age finding comparing CKDs with osteopenia to those with normal BMD

From the initial studies, 18 studies offered sufficient data for a comparative analysis of the average age differences between CKD patients with osteopenia and those with normal BMD. A meta-analysis of random effects was performed on the average age of adults. This evaluation was divided based on T-score classifications. In adult patients with osteopenia, age [SMD = 1.35, 95% CI −0.25 to 2.94, *P* = 0.10] was not significantly greater than in patients with normal BMD. Based on the CKD status, the age of adult HD patients experiencing osteopenia [SMD = 0.60, 95% CI 0.34 to 0.86, *P* < 0.001] was significantly greater than that of HD patients with normal BMD. Moreover, the age of adult PD patients exhibiting osteopenia [SMD = 0.24, 95% CI −0.15 to 0.63, *P* = 0.23] was not significantly greater than that of PD patients with normal BMD.

### Risk of bias assessment

Of 94 studies, 68 were identified as having a moderate risk of bias, whereas 15 studies were categorized with low risk and 11 with high risk of bias, respectively. Comprehensive evaluations of the ROB are available in Supplementary file, eTable 2.

## Discussion

### General comprehension

This meta-analysis marks a groundbreaking attempt to assess the prevalence of osteopenia in individuals with CKDs. Possible sources of heterogeneity among studies were investigated through predefined subgroup analysis categorized by continent, HDI level, and CKD status. The pronounced heterogeneity might arise from differences in sociodemographic and socioeconomic variables, clinical traits of the targeted samples, such as the differing levels of renal dysfunction, various factors like BMD assessed through diverse methods, patient evaluations across distinct dialysis modalities, lifestyle elements including dietary histories and sun exposure, as well as clinical targets and management approaches for CKD-MBD in both dialysis and non-dialysis CKD patients. The small number of studies included in subgroup and meta-regression analysis might lead to challenges in pinpointing the source of heterogeneity. A sensitivity analysis using a random effects model conducted by omitting one individual study at a time indicated the statistical robustness of the findings. The greatest prevalence observed in the bone area abundant in cortical content (femoral neck region). The increased prevalence of osteopenia in these areas significantly influences the outlook for these patients, raising their susceptibility to fractures and the risk of mortality.

This research uncovered a significant worldwide prevalence of osteopenia, with rates of 47% for the femoral neck, 40% for the total hip, and 35% for the lumbar region. The prevalence of osteopenia in males for femoral neck and lumbar region was 49% vs. 33% and in females for these regions was 45% vs. 35%, respectively. In hemodialysis patients, the prevalence of osteopenia in the femoral neck, total hip, and lumbar region was 46%, 44%, and 34%, respectively. One potential reason for this disparity could be linked to the reality that DEXA has certain limitations in individuals with advanced CKD. It is unable to identify a particular pathologic subtype of renal osteodystrophy (ROD) and may be influenced by heightened skeletal and extraskeletal calcification frequently observed in CKD patients as CKD advances, resulting in an underestimation of BMD results [50]. Another potential explanation could be that cortical bone seems to be influenced more than trabecular bone in ROD. Due to the substantial amount of cortical bone in the femoral neck and hip, these regions might be more prone to changes in bone density. This finding highlights the significance of evaluating total hip and femoral neck BMD when measuring BMD in CKD patients. The most frequently assessed locations for BMD evaluation are the lumbar spine and femoral neck [51]. Our results showed that the femoral neck and total hip were more vulnerable to osteopenia than the lumbar spine in CKDs. The prevalence of osteopenia was heightened when identified at these locations in CKDs; we suggest that the lumbar spine and hip regions be evaluated simultaneously for osteopenia diagnosis in CKDs.

### Potential concerns about methodological inconsistencies during ROB assessment

Regarding ROB of the included studies, the majority of the studies included, specifically 68 studies, were classified as having moderate ROB based on the JBI checklist. This classification may raise worries about the possible methodological inconsistencies of these studies among readers. Nonetheless, it is important to recognize that this might stem from the JBI checklist items, as the majority of the included studies received scores based on the aspects related to “Conducting of the data analysis in sufficient coverage of the identified sample,” “Adequacy of the response rate,” “Validity of the method for evaluating the condition,” and “The standard way of measuring the condition for all participants.” As a result, the majority of studies were classified as having a moderate ROB. A great portion of this finding can be devoted to the inclusion and exclusion criteria, as we focused on studies that utilized DEXA for BMD measurement; this approach to article selection omitted other studies reporting the outcome of interest through self-reporting or other valid tools, which their results could influence the pooled estimates. Thus, this approach cannot adversely affect the pooled estimates.

For subgroups involving the forearm area, all studies showed a moderate ROB and all studies received scores from the related items discussed. In the distal radius area, out of nine studies, six exhibited moderate ROB, two showed high ROB, and one indicated low ROB. All studies concerning the distal radius area received scores from the discussed items. According to the ROB findings of 68 studies, the items that were different were mainly associated with sample size and sampling method. For the subgroups comprising forearm and distal radius areas, also the insufficient number of studies might have resulted the fact that the estimated prevalence diverged from the true prevalence, either through underestimation or overestimation.

### Potential concerns of low BMD estimates based on regional analysis

Based on regional analysis of femoral neck osteopenia, the highest prevalence of osteopenia was found in Europe (52%) and in countries with a very high HDI level (48%). The findings of this research indicate that osteopenia is a prevalent problem in certain developed nations. The explanation for this outcome may relate to elements like the elevated frequency of healthcare appointments and the extended average lifespan of people in regions with very high HDI. The heightened disease burden noted in developed nations can be linked to their considerably larger populations. According to the regional assessment, the majority of the research carried out in Asia and Europe, particularly in developed nations with very high and high HDI levels. Even though we contemplated an extensive search across multiple databases, this research suffered from sufficient research conducted in other regions, including Africa, America, and Australia, along with medium and low HDI countries, which may restrict the generalizability of our findings. This constraint impacts not just the outcomes for every bone site but can also influence the results of other subgroups such as age and sex. To recapitulate, it is important for readers to interpret the results carefully, as the findings mostly come from Asia and Europe, particularly in countries with very high HDI, and these estimates do not accurately represent the actual prevalence of osteopenia in adult CKD patients in stages 3a-5D globally. Consequently, additional research is needed to evaluate the prevalence of bone density loss in the specified areas to yield improved estimates among these patients, which is essential for informing healthcare planning and policy-making for implementing long-term strategies for them. Recognizing the significance of estimate prevalence is linked to improving budget impact models for accurately assessing medication costs, expenses for related medical services (like office appointments, hospital stays), and forecasting the required number of physicians, nurses, and other healthcare staff and facilities essential to achieve the desired level of care availability. Furthermore, it is recommended that future research provides clearer definitions when reporting prevalence rates, especially by detailing the osteopenic rates in CKDs for each bone site instead of merely presenting as an general osteopenic rate.

### Potential sources of heterogeneity in BMD measurement across studies

Because of high accuracy and precision, DEXA is regarded as the preferred method for measuring BMD [52]. The principle behind DEXA’s application in the body is the varying absorption of x-ray photons by different tissues, from which bone density is determined by a detector based on the difference in attenuation between bone and soft tissue due to the transmission of low and high energy photons [7, 53]. The extent of variation between the computed BMD and the reference database (the average mean) is expressed as either a T-score or a Z-score. This method is utilized for numerous purposes such as detecting loss of BMD, evaluating fracture risk, tracking changes in density over time, and analyzing treatment outcomes[7]. Notably, the outcomes of BMD assessments in different studies may differ due to various factors; A) Patient preparation including ability to be motionless during the assessment, patient history such as prior surgery, previous fracture, risk factors, endocrine or metabolic diseases, and other conditions, medications-related bone loss. B) Acquisition including equipment (slight image distortion and increased scatter in DEXA scanners using fan-beam technology), quality control (the requirements including scanning of a dedicated phantom and automatic analysis, which the procedures differ by manufacturer), area of study (in cases of incorrect measurement and interpretation due to confounding factors, especially for hip or spine regions), positioning (one of the usual errors for BMD measurements is incorrect positioning, which must be manually adjusted by the technologist for region of interest). C) Analysis (considering patient’s positioning, motion, and artifacts such as barium, overlying hardware, and metal while analyzing images). D) Radiation dose. E) Variation in abdominal fat mass/folds may lead to overestimation or underestimation in BMD measurement [54, 55]. F) Differences in bone structure and microarchitecture between ethnic groups [56, 57]. G) Variation in muscle strength among different ethnicities: This difference should be considered while studying bone both within and between different ethnic groups [58, 59].

### Preferred bone site(s) for BMD measurement in CKD patients stages 3a-5D

Early identification of low BMD in CKD patients is vital, especially for those awaiting kidney transplants, since managing the condition becomes more difficult after transplantation. There is a well-recognized link between rising osteopenia levels and diminishing kidney function [60, 61]. Consequently, this research recommends that healthcare providers should closely monitor BMD in individuals with CKD across stages 3a-5D, especially advanced CKD stages. As per ISCD, to diagnose osteoporosis (T-score of -2.5 or lower), the preferred region for diagnosis is the femoral neck [26]. Moreover, as per the IOF (International Osteoporosis Foundation), the designated site for epidemiological research is the femoral neck [62]. Nonetheless, there are no specific guidelines available for determining the best bone site for evaluating bone loss in CKD/dialysis patients. In individuals with eGFR < 60 ml/min/1.73 m^2^, the hip is the preferred bone location for osteopenia/osteoporosis, which is strongly associated with hyperparathyroidism [63]. Huang et al. performed a cross-sectional study involving 11,050 CKD patients in the USA and found a positive correlation between femoral neck BMD and eGFR [64]. In individuals with predialysis CKD and those undergoing hemodialysis, prospective studies have shown that decreased BMD at the hip and forearm can forecast future fractures, and the WHO T-Score thresholds are suitable for classifying fracture risk [65–67]. While renal function plays a part in low BMD at the spine, this relationship does not appear to be robust. Although the existing KDIGO guidelines suggest utilizing DEXA for fracture prediction, the link between low BMD at the lumbar spine and fractures in CKD patients remains unproven [68–70]. Moreover, hip BMD is acknowledged as the most reliable fracture predictor for CKD patients [65, 71, 72] because PTH primarily affects cortical bone, the dominant type found in the hip, whereas the lumbar spine is mainly made up of trabecular bone [72–75]. Furthermore, when compared to BMD measurements at different anatomical sites, BMD evaluated in the hip region may act as the most dependable predictor of mortality risk in CKD patients, particularly in those with ESRD or receiving dialysis. BMD in the hip region may provide a more precise indication of bone disease and metabolic alterations in CKD patients and seems particularly important for tracking CKD prognosis [76, 77], taking into account the measurement error that could arise from confounding factors for the lumbar spine.

Lately, the distal third of the radius has been regarded as the most dependable area for predicting osteoporosis [78–80]. Taking into account that movement artifacts in forearm BMD are relatively frequent, this location is largely devoid of confounding elements and artifacts when compared to the lumbar spine site [81, 82]. Significantly, the movement artifacts when lying down might be reduced in comparison to the seated position at the table [83]. Its predictive reliability is supported by the metaphysis of this location, which encompasses both trabecular and cortical bone, aligning better with age-related variations, ultimately leading to increased spongy bone loss [84]. Moreover, the radiation dose from a typical DEXA scan for the distal forearm is 0.1 microsieverts, which is considered minimal [85]. A meta-analysis released in 2025 showed that DEXA measurement at the forearm location had excellent predictive value for central osteoporosis. A significant relationship found between forearm scans and BMD values at the lumbar site (pooled effect size 0.603, 95% CI: 0.579 to 0.627) and femoral site (pooled effect size 0.641, 95% CI: 0.600 to 0.680), indicating that distal forearm measurement serves as a useful additional option when BMD assessment in lumbar and forearm areas is difficult [86].

### Clinical implications: the link between the low BMD estimates to clinical practice

#### Risk of fracture, mortality, and vascular calcification

The clinical importance of low bone density heavily depends on the DEXA measurement tied to the quality of scan acquisition, the quality of analysis, and the quality of interpretation. Therefore, healthcare providers must be optimally trained and prepared to perform BMD measurements according to established standards to ensure precise and accurate reporting. The significance of low bone density is primarily associated with the risk of fractures, with hip fractures being the most crucial concern [87]. Recent research has shown a link between low measured BMD (whole body) and heightened fracture risk and mortality due to cardiovascular disease in patients with ESRD or undergoing hemodialysis [76, 88–90]. A recent meta-analysis has shown that lower BMD in the spine, hip, arm, and total body is linked to higher all-cause mortality in CKD patients. Reduced BMD at the hip/femoral neck is associated with the greatest risk of overall mortality in persons with CKD (pooled RR = 1.69, 95% CI: 1.20 to 2.40). Moreover, the risk of death linked to every standard deviation reduction in BMD is considerably greater at the hip/femoral neck (pooled RR = 1.43, 95% CI: 1.15 to 1.77) than at the arm (pooled RR = 1.03, 95% CI: 1.00 to 1.06) and lumbar spine (pooled RR = 1.17, 95% CI: 0.98 to 1.39) [19].

In CKD stages 3a-5D, people exhibit reduced BMD and lower mechanical strength, resulting in a significantly higher fracture risk (1.5–2 times more than the general population) and increased morbidity and mortality relative to healthy individuals [69, 91]. Growing evidence indicates that the incidence of fractures is more common in dialysis-dependent CKD patients compared to those with pre-dialysis CKD [92]. The processes believed to elevate this fracture risk include secondary hyperparathyroidism stemming from decreased phosphaturia and vitamin D deficiency caused by inadequate calcitriol production, both of which result in harmful impacts on bones [93, 94], which are linked to functional impairment, hospitalization, and increased mortality, as hip fractures account for nearly half of deaths related to osteoporosis [95]. The secondary hyperparathyroidism caused by CKD results in bone resorption, which facilitates the release of calcium and phosphorus. The link between BMD and mortality risk might be attributed to secondary hyperparathyroidism and hyperphosphatemia [15, 96]. Per the updated KDIGO CKD-MBD guidelines from 2017, it is recommended that in patients with CKD G3a-G5D who show signs of CKD-MBD and/or have osteoporosis risk factors (such as advanced age, female gender, low BMI, previous fragility fractures, family history of hip fractures, loss of height (> 4 cm), secondary osteoporosis, glucocorticoid use, high alcohol consumption and/or smoking, lengthy dialysis duration, and postmenopausal status), BMD testing should be conducted to evaluate fracture risk if the results might influence treatment choices [97], particularly for the hip, though it may not always apply to the lumbar region [65, 72, 98, 99].

Furthermore, the significance of low bone density in these patients is linked not only to a heightened fracture risk but also to its effect on outcomes such as vascular calcification and cardiovascular disease. In CKD patients, decreased bone density is connected to the altered bone-vascular axis via multiple pathways, resulting in vascular calcification like aortic and coronary artery calcification noted in ESRD patients [100, 101]. The vascular calcification caused by elevated calcium and phosphorus levels is facilitated by the induction of osteoblast-like cells derived from vascular smooth muscle cells [102]. Cardiovascular events and death-related cardiovascular events have risen as a consequence of vascular calcification in CKD patients [88–90, 103].

#### Sex-specific estimates

In addition, sex-specific evaluation of femoral neck and lumbar osteopenia shows an osteopenia rate of nearly 50% and 35% in both males and females, respectively. Considering the mean age data of osteopenic patients (61.25 years, 95% CI: 57.96 to 64.54) and CKD patients with normal BMD (55.92 years, 95% CI: 52.68 to 59.16), these findings indicate that the lumbar spine and hip regions of CKD patients ought to be eligible for DEXA scans earlier in life. Our research clearly cannot verify that gender is a significant factor for osteopenia because of the limited age data on CKDs with osteopenia found in the current literature. CKD itself causes significant alterations in mineral, hormonal, and bone metabolism, meaning that gender would not be a primary factor. These findings indicate that both male and female CKD patients ought to be eligible for DEXA scans at an earlier age compared to non-CKD people, as advised by KDIGO and EUROD guidelines [104, 105]. Enhancing bone health in elderly individuals with osteopenia is crucial since the majority of fractures happen in this demographic. The risk of fractures should be evaluated by combining BMD and clinical risk factors, and treatment should be contemplated for patients at an elevated fracture risk [24].

### Impact of CKD-MBD management on low BMD rates

#### Dialysis modalities

Individuals with CKD before transplantation exhibit elevated rates of osteopenia and osteoporosis [106, 107]. A low BMD is thus an effective indicator of future fracture risk, especially when combined with age, previous fractures, and other risk factors, highlighting the significance of this study. The capacity to identify the pathophysiologic mechanism behind the abnormality is crucial, as the early commencement of suitable treatment can prevent or improve the mineral and bone disorder that arises in late CKD. [108]. As the GFR keeps decreasing, alterations in mineral metabolism influence the bone microstructure via remodeling, a condition referred to as CKD-MBD. CKD-MBD develops early as kidney function declines and is observed in almost all patients with CKD stage 5 [109]. As renal function declines in dialysis patients, phosphorus levels rise, leading to increased PTH. Nephrologists frequently prevent bone loss by reducing the patient’s serum phosphorus and PTH concentrations. Even with these initiatives, dialysis patients are at a higher risk for osteoporosis compared to the general population [110, 111]. Proper kidney function is vital for the retention of cortical bone. While renal transplantation is viewed as the optimal choice for ESRD patients, there exists an alternative replacement therapy prior to transplantation: HD or PD. As the first replacement therapy, PD may offer advantages in terms of reduced fracture risk compared to HD, as suggested by certain studies, which could result in improved bone health among ESRD patients [112–115].

There is limited information regarding changes in BMD after starting dialysis, as well as, the possible connection to mortality. In a study with 242 ESRD patients, 138 patients underwent PD while 104 patients were treated with HD as their initial dialysis therapy, and whole-body DEXA was evaluated. During the initial year of therapy, PD demonstrated a more protective effect on BMD changes than HD. A notable decrease in BMD at various bone sites (BMD_leg, trunk, rib, pelvis, and spine_) and a reduction in BMD_total_ observed in HD patients compared to those receiving PD. Additionally, adverse alterations were noted for HD therapy compared to PD therapy, BMD_total_ (*β* =  − 0.15), BMD_head_ (*β* =  − 0.14), BMD_leg_ (*β* =  − 0.18), and BMD_trunk_ (*β* =  − 0.16) [116]. Regarding the connection between low bone density and later outcomes, fractures, with dialysis modalities in ESRD patients, the majority of research focuses on HD patients rather than PD patients. Concerning the impact of dialysis modalities on fracture incidences, a reduced fracture risk has been noted with PD in comparison to HD in certain studies [112, 113], which may lead to a decreased mortality risk. The Taiwan National Database, noted a higher risk of fractures in HD patients, highlighting that the incidence of hip fractures was 31% greater in HD patients in comparison with PD patients [112]. Additionally, in the US Renal Data System, the odds of experiencing a hip fracture were found to be 1.6 times greater in HD patients compared to PD patients [113]. In a meta-analysis published in 2015, BMD among CKD patients of stages 3–5 with fracture was lower compared to those without, which was noted both in the 1/3 radius and lumbar spine regions. This result was observed among both pre-dialysis and dialysis patients which emphasized the importance of assessing BMD among these patients for preventing future risk of fracture [117].

Although the mechanism (s) related to the effect of dialysis modalities (PD compared to HD) on risk of fracture has not been fully comprehended, it may be related to the residual renal function, which may be better preserved in PD versus HD [113]. The ongoing nature of PD might be linked to the beneficial effects of PD over HD on BMD. In fact, the fluctuations of fluids and solutes are not seen in PD therapy when compared to HD. Consequently, the levels of circulating solutes, including electrolytes associated with CKD-MBD like phosphate and calcium, as well as acid–base balance, remain stable, potentially resulting in improved preservation of residual renal function; therefore, they may contribute to better retention of BMD and a lower rate of hip fractures in PD patients compared to HD patients [112–115]. An alternative explanation related to the protective role of PD after starting dialysis might be related to the bone strength, which its loss may be lesser in PD compared to HD. This positive impact of PD over HD might be connected to factors influencing bone strength and mass, such as nutritional status and muscle strength, which require further investigation in future research [116].

#### Vitamin D supplementation

As kidney function declines, advancing vitamin D deficiency and hyperphosphatemia will promote hypertrophy of the parathyroid gland and aid in the onset of secondary hyperparathyroidism. The buildup of secondary hyperparathyroidism and uremic toxins subsequently speeds up bone turnover by stimulating osteoclastogenesis and enhancing the release of phosphate and calcium from the bone [118]. Hyperphosphatemia diminishes vitamin D function in the kidneys and causes a deficiency in calcitriol (active form of vitamin D), resulting in impaired bone mineralization and increased fragility [119]. A drop in 1, 25dihydroxyvitamin D may cause hypocalcemia, leading to continuous PTH secretion and consequently resulting in secondary hyperparathyroidism [120]. A lack of vitamin D worsens secondary hyperparathyroidism and increases the chances of proteinuria, abnormal bone turnover and mineralization, bone loss, and mortality [96, 121]. Nonetheless, proteinuria may lead to a deficiency in vitamin D as a result of a reduction in calcium-binding proteins [122, 123]. Regarding the site-specific impact of vitamin D on BMD in a CKD population, vitamin D deficiency is more reliably linked to reduced BMD in the femoral and hip regions compared to the lumbar spine [124]. Sarcopenia may lead to a decrease in BMD due to reduced mechanical stimulation and the existence of proinflammatory substances associated with sarcopenia [125]. Sarcopenia and a deficiency in vitamin D collectively worsen the bone loss in the femor and hip regions [126].

Vitamin D supplementation is advised early in CKD, but its optimal use is still being debated [127, 128]. At present, there is no distinct guideline endorsing one formulation of nutritional vitamin D over others, nor is there clarity on the benefits of combining nutritional vitamin D with activated vitamin D for CKD patients [129]. In a study involving hemodialysis patients with 25(OH)D levels < 30 ng/ml that were randomized to receive either a placebo or cholecalciferol repletion (25,000IU, weekly orally), no fractures were reported in the active treatment group; however, ten fractures occurred among nine patients in the placebo group, with half of these fractures happening within the initial 13 weeks, which appeared that this difference cannot be attributed to the intervention [130]. Another study evaluated the combined effect of cholecalciferol alongside cinacalcet/calcitriol among 60 HD patients suffering from severe secondary hyperparathyroidism. Patients received a stable dosage of cinacalcet and calcitriol, which were adjusted based on various conditions during the study and were randomly assigned to receive either a placebo or cholecalciferol (5000 IU/day). Almost 40% of patients experienced greater than 10% improvement in BMD of the femoral neck, although this was not significant after 24 weeks [131]. In a systematic review and meta-analysis of RCTs (with follow-up exceeding 3 months) involving adult patients with CKD stages 3, 4, or 5 (including those on dialysis), vitamin D demonstrated ambiguous effects on fractures when compared to placebo (RR: 0.68, 95% CI: 0.37 to 1.23) [132].

Currently, there is nonetheless insufficient evidence demonstrating the benefits of sole vitamin D supplementation in reducing bone loss and fracture risks in both children and adults with CKD as stated by the expert panel of the European Renal Osteodystrophy initiative from the European Renal Association (ERA) and the European Society for Paediatric Nephrology (ESPN) [133]. According to the KDIGO 2017 Clinical Practice Guideline Update, the possibility of vitamin D deficiency should be evaluated in adult patients with CKD stage G3a-G5 (GFR < 60 mL/min/1.73 m^2^, not receiving dialysis). Nonetheless, there is no existing agreement on the way how to measure and administer vitamin D [4, 17]. Additionally, it has been proposed that vitamin D analogs (such as paricalcitol, doxercalciferol, and alfacalcidol) and calcitriol should not be routinely prescribed for these patients, unless in cases of CKD G4-G5 with advancing and severe secondary hyperparathyroidism [4]. For patients undergoing dialysis, the benefits of active vitamin D are outlined in its ability to manage PTH [134, 135], demonstrating greater effectiveness during dialysis compared to home treatments [136].

### Age- and sex-specific prevalence estimates

#### Sex-specific analysis

According to the sex-specific osteopenic rates found in the studies, the estimated prevalence of osteopenia among both males and females was significant. Bone loss in renal bone disease is specific to certain locations, and in cases of both primary and secondary hyperparathyroidism, the loss of cortical bone surpasses that of trabecular bone [137]. Our research demonstrated that in cases of femoral neck osteopenia, men experience greater cortical bone loss compared to women (49% versus 45%), whereas this comparison for lumbar region was 33% versus 35%. Moreover, the prevalence of osteopenia among male and female HD patients was 33% (95% CI: 27 to 40) and 34% (95% CI: 30 to 38), respectively. The potential explanation for the difference between men and women among these regions might be due to the rate of cortical bone loss in both genders [138, 139]. Furthermore, this difference in bone density loss may be resulted from the varying CKD status and duration of dialysis [140]. Moreover, this difference in results may be due to the underestimation of bone density loss that could arise from confounding factors for the lumbar spine region. The significant rate of bone loss across genders results from various factors. Women, particularly those who are postmenopausal, are more prone to developing osteoporosis than experiencing bone loss [141]. The proportion of women with osteopenia continuously increases, showing similar patterns in men. Women between the ages of 45 and 50 have a 24% prevalence of osteopenia, reaching a high of 66% at ages 64 to 69, then decreasing slightly to 60% by ages 80–84 as osteoporosis prevalence rises. In males, the prevalence of osteopenia is 28% among those aged 45 to 49 years and 63–64% for individuals aged 64–79 years. Consequently, most men and women exhibit bone density levels indicative of osteopenia after reaching the age of 60 [142]. Also, upon turning 50, there is a notable decline in muscle mass, accompanied by similar gender-neutral transformations including heightened aging of satellite cells and inflammation, diminished protein synthesis, and myocyte regeneration, along with various other sex-specific changes resulting from lower sex hormone levels [143], which lower levels of sex hormones in both genders, frequently observed in the natural course of CKD [144]. In addition, this loss may be attributed to variations in BMD at maturity among men and women [138, 139]. Although osteoporosis is more common in women of all ages, the differing pattern of bone loss indicates that men should prioritize early prevention and take a proactive stance like women to halt bone loss progression [141]. At a greater rate than women, men tend to remain in the osteopenia stage, thus men’s bone health must also be considered important [145].

#### Age-specific analysis

In this research, comparing the age data among CKD patients, the age of patients with osteopenia was not significantly greater than in patients with normal BMD among 18 studies. This result may be resulted from the heterogeneity of the included studies, particularly in terms of dialysis modality, which 3 studies were ND (all included showed significant results), ten were HD patients (two were significant), and five were PD patients (one was significant). Interpretation based on the findings of age results according to dialysis modalities subgroups showed that the age of adult HD patients experiencing osteopenia was the only subgroup that was significantly greater than that of HD patients with normal BMD, which the effect was moderate to large (SMD = 0.60, 95% CI: 0.34 to 0.86). The inconsistencies regarding the age results across various dialysis modalities may result from the differences in the sample size of the studies between the groups, considering different genders between the groups, the misconception related to the osteopenia and normal BMD categorization according to the ISCD definition, for example, the T-scores of −1.0, −1.0, and −1.0 for the L1-L4 vertebrae, femoral neck, and total hip are viewed as normal BMD, etc.

### The importance of psychological issues in CKD patients

Osteopenia acts as a precursor to osteoporosis, appearing with a gradual onset. Once it progresses to osteoporosis, it heightens fracture risk for patients leading to both physical and psychological impacts, imposing a significant burden on families and society. Psychosocial issues negatively impact the quality of life for CKD patients and lead to overall unfavorable outcomes of CKD [146, 147]. Depression associated with CKD is linked to worse health outcomes, such as higher mortality rates [148], reduced self-assessed quality of life [149–151], greater healthcare usage [152], and diminished treatment adherence [153]. Based on the systematic review and meta-analysis in 2024, clinical depression is common among those with CKD, which the overall prevalence of depression in the CKD population was 27.6% (95% CI: 23.9 to 31.5). The rate among chronic hemodialysis patients was 31.1% (95% CI: 25.3 to 37.2, *n* = 42 studies), while this rate was 18.9% (95% CI: 11.9 to 27.1, *n* = 10 studies) for those before dialysis. Furthermore, the prevalence of depression was higher in the hemodialysis group than in the peritoneal dialysis group, 31.9% (95% CI: 26.0 to 38.1, *n* = 41 studies) vs. 20.4% (95% CI: 13.1 to 28.7, *n* = 3 studies) [154]. Healthcare professionals ought to intentionally assess CKD patients for depression, particularly those in the later stages of the condition. Observing bone density in patients undergoing dialysis is crucial. Since bone loss shows no symptoms, it is often overlooked. It is essential to routinely assess the BMD of dialysis patients to identify those at high risk of fractures thanks to physical and psychological impacts, medical expenses, and mortality.

## Limitations, strengths, and recommendations

As far as we are aware, this systematic review and meta-analysis is the inaugural effort to specifically assess the prevalence of osteopenia in CKDs. The analysis of our results needs to consider various limitations. Firstly, many of the findings in this review rely on observational data, leading to worries about the significant heterogeneity among the included studies, potentially stemming from various factors such as the studies’ inclusion criteria, including the date of the study, the population studied, sample size, ethnicity, and gender distribution. We aimed to lessen the influence of heterogeneity in our analysis by utilizing a random effects model for the meta-analysis, which assumes that the main studies are varied and offers a more conservative effect estimate. We also made efforts to tackle bias via meta-regression and subgroup analyses. In addition, our research is influenced by language bias and an absence of searching for grey literature. Additionally, in this meta-analysis, information pertaining to study design was not utilized, and our analyses were inconsistent across the studies. The use of various designs and approaches might have resulted in hidden biases, potentially threatening the reliability of our findings. Concerning methodological quality, numerous studies displayed medium quality, potentially impacting the validity of overall findings and restricting the generalizability of results, even within the evaluated areas; thus, we recommend performing additional high-quality research to enhance our comprehension of osteopenic prevalence in CKDs. To address these limitations, we have implemented various strategies, including the use of random effects models in our analyses and conducting evaluations based on definitions including gender, CKD status, HDI tier, and continent levels. In addition, most of the research included in our meta-analysis comes from Asia and Europe, with insufficient studies available from Africa, Australia, and, to a lesser extent, America; as a result, the scarcity of research in these areas restricts the capacity to accurately represent the global prevalence. Therefore, additional epidemiological studies are suggested to assess the prevalence of osteopenia in countries across these continents.

Most of the research included in the analysis was performed in nations with high and very high HDI, which could restrict the applicability of the results to other areas. Consequently, care must be taken when extending our results to other nations, and we advocate for acquiring more accurate and country-specific prevalence rates, especially in medium and low HDI countries in the future. Furthermore, to draw more conclusive insights, further studies exploring the impact of ethnicity on bone loss among CKDs are necessary, considering the varying outcomes noted across different countries. Furthermore, conclusive statements about osteopenic rates are challenging due to variations in outcome definitions, statistical adjustments, types of BMD measurement devices used, and reference data for BMD measurements across different studies. Moreover, socioeconomic elements might have limited access to DEXA in specific nations, possibly leading to the concern of underdiagnosed osteopenia among CKD patients. Furthermore, patients’ weight, instead of height, accounted for the relationship between BMI and BMD [155]. This finding might lead to a specific amount of measurement error associated with DEXA scans in people with obesity [156, 157]. While DEXA is still the gold standard for assessing BMD, the presence of osteophytes, facet sclerosis, and abdominal aortic calcification (AAC) can result in unanticipated overestimation of BMD readings in the lumbar region and inconsistent BMD outcomes between the lumbar and femor regions [158]. The majority of published research on BMD in CKD patients, particularly those with ESRD, typically excludes densitometry of the distal third of the radius, an area primarily composed of cortical bone. Further studies reporting the densitometry of the distal third of the radius on CKDs are necessary. In addition, considering the trim and fill analysis, the prevalence of osteopenia in CKDs is influenced by publication bias; thus, upcoming systematic reviews and meta-analyses ought to conduct more extensive searches to address this issue.

Nonetheless, our research has several important advantages. This meta-analysis combines data from 94 studies, rendering it the most extensive investigation to date regarding the prevalence of osteopenia in CKD patients stages 3a-5D. The search approach uncovered a significant number of studies and participants, facilitating a clearer understanding of LBMD across various CKD status, bone locations, genders, regions, and HDI tier.

## Conclusion

In summary, our systematic review and meta-analysis showed a significant global prevalence of osteopenia, with the highest rates observed in bone sites abundant in cortical content (femoral neck region). Sex-specific evaluation of femoral neck osteopenia shows an osteopenia rate of nearly 50% in both males and females. Our results show that Europe exhibited the highest prevalence of osteopenia, particularly at the femoral neck and lumbar sites. Taking into account the rate of osteopenia among CKD status, dialysis patients exhibited higher prevalence of osteopenia in comparison to non-dialysis patients. This information is crucial for guiding healthcare planning and policy-making, offering understanding of existing trends and future forecasts, and facilitating the development of long-term epidemiological strategies and required treatment resources for those with osteopenia, thus alleviating the serious risks of fractures and lowering related mortality rates. Due to the considerable clinical, economic, and social consequences of osteoporosis, precise prevalence estimates of osteopenia are essential for guiding policy choices. These choices influence how individuals in need of treatment are identified and their ability to access drug therapies and continuous monitoring for fracture risk reduction.

## Supplementary Information

Below is the link to the electronic supplementary material.

Supplementary file1 (DOCX 479 KB)

## Data Availability

All the data involved in this study are provided in supplementary file, eTable 4 and efigures 1–22.

## Abbreviations

CKD: Chronic kidney disease
eGFR: Estimated glomerular filtration rate
JBI: Joanna Briggs Institute
ESRD: End-stage renal disease
DEXA: Dual-energy X-ray absorptiometry
BMD: Bone mineral density
CKD-MBD: Chronic kidney disease–mineral bone disorder
PTH: Parathyroid hormone
NHANES III: Third National Health and Nutrition Examination Survey
HDI: Human development index
SMD: Standardized mean difference
MBD: Mineral bone disorder
ROD: Renal osteodystrophy
KDIGO: Kidney Disease Improving Global Outcomes
EUROD: European Renal Osteodystrophy
LBMD: Low BMD
PRISMA: Preferred Reporting Items for Systematic Reviews and Meta-Analyses
MeSH: Medical Subject Headings
ISCD: International Society for Clinical Densitometry
UNDP: United Nations Development Programme
GDP: Gross Domestic Product
ROB: Risk of bias
JBI: Joanna Briggs Institute
ND: Non-dialysis
HD: Hemodialysis
PD: Peritoneal dialysis
HD + PD: Hemodialysis plus peritoneal dialysis
WHO: World Health Organization
AAC: Abdominal aortic calcification
ERA: European Renal Association
ESPN: European Society for Paediatric Nephrology
