# Supplementary material for: Global and regional prevalence of osteopenia in chronic kidney disease: a systematic review and meta-analysis
Source: Clin Exp Med. 2025 Nov 11;25(1):362. doi: 10.1007/s10238-025-01909-3 (PMC12605369; doi:10.1007/s10238-025-01909-3)
Supplement: Supplementary file 1 — Supplementary file1 (DOCX 479 KB) [file 10238_2025_1909_MOESM1_ESM.docx]

**Online Supplementary file:**

1. List of supplementary file, **page 1.**
2. Search strategies, **pages 2-4.**
3. **eTable 1.** List of excluded studies at full-text screening stage with brief reasons, **pages 5-24.**
4. **eTable 2.** Risk of bias assessment of included studies according to the JBI checklist, **pages 25-28.**
5. **eTable 3.** The characteristics of included studies, **pages 29-32.**
6. **eTable 4.** The osteopenic rate of the included studies, **pages 33-36.**
7. **eFigures 1-10, pages 37-46.**
8. **References, pages 47-52.**

**Search strategies.** Search syntaxes based on each database.

**Pubmed = 3716**

* ((Chronic kidney disease[tiab] OR Chronic renal disease[tiab] OR Chronic kidney insufficiency[tiab] OR Chronic renal insufficiency[tiab] OR Kidney disease[tiab] OR Renal disease[tiab] OR Kidney insufficiency[tiab] OR Renal insufficiency[tiab] OR Kidney failure[tiab] OR Renal failure[tiab] AND Osteopenia[tiab] OR Bone loss[tiab] OR bone mineral density[tiab] OR bone health[tiab] OR bone density[tiab]))

** (Non-dialysis[tiab] OR Predialysis[tiab] OR Pre-dialysis[tiab] OR Hemodialysis[tiab] OR Haemodialysis[tiab] OR Hemofiltration[tiab] OR Haemofiltration[tiab] OR Hemodiafiltration[tiab] OR Haemodiafiltration[tiab] OR Peritoneal dialysis[tiab]) AND (Osteopenia[tiab] OR Bone loss[tiab] OR Bone mineral density[tiab] OR Bone health[tiab] OR Bone density[tiab])

**Web of Science = 8572**

* ((TS=Chronic kidney disease OR TS=Chronic renal disease OR TS=Chronic kidney insufficiency OR TS=Chronic renal insufficiency OR TS=Kidney disease OR TS=Renal disease OR TS=Kidney insufficiency OR TS=Renal insufficiency OR TS=Kidney failure OR TS=Renal failure) AND (TS=Osteopenia OR TS=Bone loss OR TS=Bone mineral density OR TS=bone health OR TS=bone density))

** ((TS=Non-dialysis OR TS=Predialysis OR TS=Pre-dialysis OR TS=Hemodialysis OR TS=Haemodialysis OR TS=Hemofiltration OR TS=Haemofiltration OR TS=Hemodiafiltration OR TS=Haemodiafiltration OR TS=Peritoneal dialysis) AND (TS=Osteopenia OR TS=Bone loss OR TS=Bone mineral density OR TS=Bone health OR TS=Bone density))

**Scopus = 4361**

* ((TITLE-ABS(“Chronic kidney disease”) OR TITLE-ABS(“Chronic renal disease”) OR TITLE-ABS(“Chronic kidney insufficiency”) OR TITLE-ABS(“Chronic renal insufficiency”) OR TITLE-ABS(“Kidney disease”) OR TITLE-ABS(“Renal disease”) OR TITLE-ABS(“Kidney insufficiency”) OR TITLE-ABS(“Renal insufficiency”) OR TITLE-ABS(“Kidney failure”) OR TITLE-ABS(“Renal failure”)) AND (TITLE-ABS(Osteopenia) OR TITLE-ABS(“Bone loss”) OR TITLE-ABS(“Bone mineral density”) OR TITLE-ABS(“Bone health”) OR TITLE-ABS(“Bone density”)))

** ((TITLE-ABS(“Non-dialysis”) OR TITLE-ABS(“Predialysis”) OR TITLE-ABS(“Pre-dialysis”) OR TITLE-ABS(“Hemodialysis”) OR TITLE-ABS(“Haemodialysis”) OR TITLE-ABS(“Hemofiltration”) OR TITLE-ABS(“Haemofiltration”) OR TITLE-ABS(“Hemodiafiltration”) OR TITLE ABS(“Haemodiafiltration”) OR TITLE-ABS(“Peritoneal dialysis”)) AND (TITLE-ABS(Osteopenia) OR TITLE-ABS(“Bone loss”) OR TITLE-ABS(“Bone mineral density”) OR TITLE-ABS(“Bone health”) OR TITLE-ABS(“Bone density”)))

**Science direct = 5271**

* ((TITLE-ABS-KEY(Chronic kidney disease) OR TITLE-ABS-KEY(Chronic renal disease) OR TITLE-ABS-KEY(Chronic kidney insufficiency) OR TITLE-ABS-KEY(Chronic renal Insufficiency) OR TITLE-ABS-KEY(Kidney disease) OR TITLE-ABS-KEY(Renal disease) OR TITLE-ABS-KEY(Kidney insufficiency) OR TITLE-ABS-KEY(Renal insufficiency) OR TITLE-ABS-KEY(Kidney failure) OR TITLE-ABS-KEY(Renal failure)) AND (TITLE-ABS-KEY(Osteopenia) OR TITLE-ABS-KEY(Bone loss) OR TITLE-ABS-KEY(Bone mineral density) OR TITLE-ABS-KEY(Bone health) OR TITLE-ABS-KEY(Bone density)))

** ((TITLE-ABS-KEY(Non-dialysis) OR TITLE-ABS-KEY(Predialysis) OR TITLE-ABS-KEY(Pre-dialysis) OR TITLE-ABS-KEY(Hemodialysis) OR TITLE-ABS-KEY(Haemodialysis) OR TITLE-ABS-KEY(Hemofiltration) OR TITLE-ABS-KEY(Haemofiltration) OR TITLE-ABS-KEY(Hemodiafiltration) OR TITLE ABS(Haemodiafiltration) OR TITLE-ABS-KEY(Peritoneal dialysis)) AND (TITLE-ABS-KEY(Osteopenia) OR TITLE-ABS-KEY(Bone loss) OR TITLE-ABS-KEY(Bone mineral density) OR TITLE-ABS-KEY(Bone health) OR TITLE-ABS-KEY(Bone density)))

**Embase = 3505**

* ((“Chronic kidney disease”:ti,ab OR “Chronic renal disease”:ti,ab OR “Chronic kidney insufficiency”:ti,ab OR “Chronic renal insufficiency”:ti,ab OR “Kidney disease”:ti,ab OR “Renal disease”:ti,ab OR “Kidney insufficiency”:ti,ab OR “Renal insufficiency”:ti,ab OR “Kidney failure”:ti,ab OR “Renal failure”:ti,ab) AND (Osteopenia:ti,ab OR “Bone loss”:ti,ab OR “Bone mineral density”:ti,ab OR “Bone health”:ti,ab OR Bone density:ti,ab))

** ((“Non-dialysis”:ti,ab OR “Predialysis”:ti,ab OR “Pre-dialysis”:ti,ab OR “Hemodialysis”:ti,ab OR “Haemodialysis”:ti,ab OR “Hemofiltration”:ti,ab OR “Haemofiltration”:ti,ab OR “Hemodiafiltration”:ti,ab OR “Haemodiafiltration”:ti,ab OR “Peritoneal dialysis”:ti,ab OR “Non-dialysis dependent chronic kidney disease”:ti,ab) AND (Osteopenia:ti,ab OR “Bone loss”:ti,ab OR “Bone mineral density”:ti,ab OR “Bone health”:ti,ab OR Bone density:ti,ab))

**Google Scholar = 128**

allintitle: "Chronic kidney disease" + "Bone mineral density”

**eTable 1.** List of excluded studies at full-text screening stage with brief reasons.

| No. | Author/Year | Title | Brief report for excluded studies |
| --- | --- | --- | --- |
| 1 | Avramovski et al., 2012 | The Progression of Bone Mineral Density Loss in Dialysis Patients Compared with the General Population | Insufficient data reporting |
| 2 | Fouad et al., 2015 | The Impact of Chronic Kidney Disease - Mineral and Bone Disorder on the Locomotor System and Quality of Life in Hemodialysis Patients | Insufficient data reporting |
| 3 | Hashimoto et al., 2021 | Calcium‑based phosphate binder use is associated with lower risk of osteoporosis in hemodialysis patients | Insufficient data reporting |
| 4 | Kinsella et al., 2010 | Moderate Chronic Kidney Disease in Women Is Associated with Fracture Occurrence Independently of Osteoporosis | Insufficient data reporting |
| 5 | Malluche et al., 2014 | Bone Mineral Density and Serum Biochemical Predictors of Bone Loss in Patients with CKD on Dialysis | Insufficient data reporting |
| 6 | Marques et al., 2017 | Biopsy vs. peripheral computed tomography to assess bone disease in CKD patients on dialysis: differences and similarities | Insufficient data reporting |
| 7 | Myong et al., 2013 | Relationship between Bone Mineral Density and Moderate to Severe Chronic Kidney Disease among General Population in Korea | Insufficient data reporting |
| 8 | NOURI et al., 2008 | Bone Mineral Density in Kidney Transplant Recipients and Patients on Hemodialysis: A Comparison With Healthy Individuals | Insufficient data reporting |
| 9 | Pongchaiyakul et al., 2005 | Bone Mineral Density among Patients Undergoing Continuous Ambulatory Peritoneal Dialysis (CAPD) | Insufficient data reporting |
| 10 | Wu et al., 2010 | The Association of Serum Osteoprotegerin and Osteoporosis in Postmenopausal Hemodialysis Patients: A Pilot Study | Insufficient data reporting |
| 11 | Yenchek et al., 2012 | Bone Mineral Density and Fracture Risk in Older Individuals with CKD | Insufficient data reporting |
| 12 | Jafari et al., 2021 | T Scores, FRAX, Frailty Phenotype, Falls, and Its Relationship to Fractures in Patients on Maintenance Hemodialysis | Insufficient data reporting |
| 13 | Harris et al., 2024 | Bone Mineral Density T-Score is an Independent Predictor of Major Blood Loss in Adult Spinal Deformity Surgery | Insufficient data reporting |
| 14 | Davenport et al., 2023 | Calcium balance in peritoneal dialysis patients treated by continuous ambulatory peritoneal dialysis (CAPD) and automated peritoneal dialysis (APD) cyclers | Insufficient data reporting |
| 15 | Huang et al., 2020 | Association between Bone Mineral Density and Severity of Chronic Kidney Disease | Insufficient data reporting |
| 16 | Jung et al., 2014 | Renal Function Is Associated with Bone Mineral Density and Arterial Stiffness in Healthy Postmenopausal Women | Insufficient data reporting |
| 17 | Toussaint et al., 2006 | Calcium phosphate metabolism and bone mineral density with nocturnal hemodialysis | Insufficient data reporting |
| 18 | West et al., 2015 | Bone Mineral Density Predicts Fractures in Chronic Kidney Disease | Insufficient data reporting |
| 19 | Yavropoulou et al., 2017 | Bone Quality Assessment as Measured by Trabecular Bone Score in PatientsWith End-Stage Renal Disease on Dialysis | Insufficient data reporting |
| 20 | Ghasem-Zadeh et al., 2022 | Bone microarchitecture and estimated failure load are deteriorated whether patients with chronic kidney disease have normal bone mineral density, osteopenia or osteoporosis | Insufficient data reporting |
| 21 | Janckila et al., 2009 | Tartrate-resistant acid phosphatase isoform 5a as an inflammation marker in end-stage renal disease | Insufficient data reporting |
| 22 | Tamimi et al., 2024 | Bone mineral density and related clinical and laboratory factors in peritoneal dialysis patients: Implications for bone health management | Insufficient data reporting |
| 23 | Yamanouchi et al., 2013 | Bone mineral density 5 years after parathyroidectomy in hemodialysis patients with secondary hyperparathyroidism | Insufficient data reporting |
| 24 | Montenegro et al., 2022 | Osteosarcopenia in Patients with Non-Dialysis Dependent Chronic Kidney Disease | Insufficient data reporting |
| 25 | Orlic et al., 2017 | Forearm bone mass predicts mortality in chronic hemodialysis patients | Insufficient data reporting |
| 26 | Zhang et al., 2024 | Surgical vs. conservative treatment for hip osteoporotic fracture in maintenance hemodialysis patients: a retrospective analysis | Insufficient data reporting |
| 27 | Yoshikoshi et al., 2024 | Prevalence of osteosarcopenia and its association with mortality and fractures among patients undergoing hemodialysis | Insufficient data reporting |
| 28 | Yong et al., 2023 | Prevalence and outcomes associated with hypocalcaemia and hypercalcaemia among pre-dialysis chronic kidney disease patients with mineral and bone disorder | Insufficient data reporting |
| 29 | Yang et al., 2024 | The relationship between uric acid and bone mineral density in the intermediate stage of CKD 1-3 | Insufficient data reporting |
| 30 | Xu et al., 2024 | Knowledge, attitude, and practice toward osteoporosis among patients with chronic kidney disease in Zhejiang | Insufficient data reporting |
| 31 | Wu et al., 2024 | The relationship between dietary inflammatory index and bone mineral density in CKD patients | Insufficient data reporting |
| 32 | Tavares et al., 2024 | Exploring the impact of short daily haemodialysis on muscle strength and bone health in end-stage kidney disease patients | Insufficient data reporting |
| 33 | Piñon-Ruiz et al., 2024 | Assessment of body composition by dual-energy X-Ray absorptiometry in renal transplant patients, hemodialysis patients, and a control group of healthy subjects | Insufficient data reporting |
| 34 | Park et al., 2024 | Skeletal Muscle Measurements Based on Abdominal Computerized Tomography (CT) Predict Risk of Osteoporosis in Incident Hemodialysis Patients | Insufficient data reporting |
| 35 | Moreno-González et al., 2024 | Kidney function and other associated factors of sarcopenia in community-dwelling older adults: The SCOPE study | Insufficient data reporting |
| 36 | de Castro et al., 2024 | Effects of cluster set resistance training on bone mineral density and markers of bone metabolism in older hemodialysis subjects: A pilot study | Insufficient data reporting |
| 37 | Liu et al., 2024 | Circulating FGF-23 Is Associated With Increased Risk of Osteoporosis and Fractures in Hemodialysis Patients: A Prospective Observational Study | Insufficient data reporting |
| 38 | Lee et al., 2024 | Erythropoietin treatment and osteoporotic fracture risk in hemodialysis patients: A nationwide population-based study | Insufficient data reporting |
| 39 | Lee et al., 2024 | The association between bone density of lumbar spines and different daily protein intake in different renal function | Insufficient data reporting |
| 40 | Kuang et al., 2024 | Risk factors and clinical prediction models for osteoporosis in pre-dialysis chronic kidney disease patients | Insufficient data reporting |
| 41 | Kratochvílová et al., 2024 | Increase in lumbar spine but not distal radius bone mineral density in adults after pancreas kidney transplantation | Insufficient data reporting |
| 42 | Komaba et al., 2024 | Lower Parathyroid Hormone Levels are Associated With Reduced Fracture Risk in Japanese Patients on Hemodialysis | Insufficient data reporting |
| 43 | Kim et al., 2024 | Effect of Denosumab on Bone Health, Vascular Calcification, and Health-Related Quality of Life in Hemodialysis Patients with Osteoporosis: A Prospective Observational Study | Insufficient data reporting |
| 44 | Kato et al., 2024 | Long-term effects of denosumab on bone mineral density and turnover markers in patients undergoing hemodialysis | Insufficient data reporting |
| 45 | Kato et al., 2024 | Calcium-based phosphate binders and bone mineral density in patients undergoing hemodialysis: a retrospective cohort study | Insufficient data reporting |
| 46 | Iseri et al., 2024 | Association between CKD-MBD and hip-bone microstructures in dialysis patients | Insufficient data reporting |
| 47 | Hashimoto et al., 2024 | The effect of antihypertensive therapy on skeletal muscle mass and bone mineral density in patients with end-stage kidney disease | Insufficient data reporting |
| 48 | Errihani et al., 2024 | Risk Factors for Osteoporosis in Chronic Hemodialysis Patients | Insufficient data reporting |
| 49 | Chen et al., 2024 | Denosumab Decreases Epicardial Adipose Tissue Attenuation in Dialysis Patients with Secondary Hyperparathyroidism and Low Bone Mass | Insufficient data reporting |
| 50 | Mendoza Carrera et al., 2024 | Uric Acid Correlates with Serum Levels of Mineral Bone Metabolism and Inflammation Biomarkers in Patients with Stage 3a–5 Chronic Kidney Disease | Insufficient data reporting |
| 51 | Bover et al., 2024 | Osteoporosis management in patients with chronic kidney disease (ERCOS Study): a challenge in nephrological care | Insufficient data reporting |
| 52 | Avila et al., 2024 | Inflammation and Vitamin D Receptor Polymorphism: Impact on All-Cause and Cardiovascular Mortality in Mexican Women on Dialysis | Insufficient data reporting |
| 53 | Xiang et al., 2023 | Sarcopenia and osteosarcopenia among patients undergoing hemodialysis | Insufficient data reporting |
| 54 | Ozawa et al., 2023 | The implication of calf circumference and grip strength in osteoporosis and bone mineral density among hemodialysis patients | Insufficient data reporting |
| 55 | Lv et al., 2023 | Associated factors of osteoporosis and vascular calcification in patients awaiting kidney transplantation | Insufficient data reporting |
| 56 | Lee et al., 2023 | Serum Intact Fibroblast Growth Factor 23 Levels Are Negatively Associated with Bone Mineral Density in Chronic Hemodialysis Patients | Insufficient data reporting |
| 57 | Kashgary et al., 2023 | Incidence of bone fractures among patients on maintenance hemodialysis | Insufficient data reporting |
| 58 | Kanagalingam et al., 2023 | Reducing the risk of denosumab-induced hypocalcemia in patients with advanced chronic kidney disease: a quality improvement initiative | Insufficient data reporting |
| 59 | Han et al., 2023 | Interaction Effect of Phase Angle and Age on Femoral Neck Bone Mineral Density in Patients with Non-Dialysis Chronic Kidney Disease Stage 5 | Insufficient data reporting |
| 60 | Gronskaya et al., 2023 | Denosumab for osteoporosis in patients with primary hyperparathyroidism and mild-to-moderate renal insufficiency | Insufficient data reporting |
| 61 | Fassio et al., 2023 | Radiofrequency echographic multi-spectrometry and DXA for the evaluation of bone mineral density in a peritoneal dialysis setting | Insufficient data reporting |
| 62 | Chen et al., 2023 | The relationship between skeletal muscle mass and bone mass at different sites in older adults | Insufficient data reporting |
| 63 | Chaturvedy et al., 2023 | Relationship between biochemical parameters of mineral bone disease and static bone histomorphometry in chronic kidney disease patients on hemodialysis: An Indian cross-section study | Insufficient data reporting |
| 64 | Anumas et al., 2023 | The Association of Beta-Blocker Use and Bone Mineral Density Level in Hemodialysis Patients: A Cross-Sectional Study | Insufficient data reporting |
| 65 | Young et al., 2022 | Risk Factors for Fracture in Patients with Coexisting Chronic Kidney Disease and Type 2 Diabetes: An Observational Analysis from the CREDENCE Trial | Insufficient data reporting |
| 66 | Wu et al., 2022 | Role of Fracture Risk Assessment Tool and Bone Turnover Markers in Predicting All-Cause and Cardiovascular Mortality in Hemodialysis Patients | Insufficient data reporting |
| 67 | Wang et al., 2022 | Bone Marrow Adiposity, Bone Mineral Density and Wnt/β-catenin Pathway Inhibitors Levels in Hemodialysis Patients | Insufficient data reporting |
| 68 | Wakasugi et al., 2022 | Polypharmacy, chronic kidney disease, and incident fragility fracture: a prospective cohort study | Insufficient data reporting |
| 69 | Uhlinova et al., 2022 | Significant associations between bone mineral density and vascular calcification in patients with different stages of chronic kidney disease | Insufficient data reporting |
| 70 | Udomkarnjananun et al., 2022 | Effects of phosphate binders on bone biomarkers and bone density in haemodialysis patients | Insufficient data reporting |
| 71 | Roy et al., 2022 | 25-OH vitamin D threshold for optimal bone mineral density in elderly patients with chronic kidney disease | Insufficient data reporting |
| 72 | Nawar et al., 2022 | The Relationship between Serum Osteopontin level and Parameters of Chronic Kidney Disease – Mineral Bone Disease in Patients on Regular Hemodialysis | Insufficient data reporting |
| 73 | Malle et al., 2022 | Usefulness of the trabecular bone score in maintenance dialysis patients : A single center observational study | Insufficient data reporting |
| 74 | Kim et al., 2022 | Low bone mineral density is associated with coronary arterial calcification progression and incident cardiovascular events in patients with chronic kidney disease | Insufficient data reporting |
| 75 | Kim et al., 2022 | Serum calcification propensity and its association with biochemical parameters and bone mineral density in hemodialysis patients | Insufficient data reporting |
| 76 | Kang et al., 2022 | Association between the appendicular lean mass index or handgrip strength and bone mineral density in patients undergoing peritoneal dialysis | Insufficient data reporting |
| 77 | Jirasirirak et al., 2022 | Prevalence and predictors of asymptomatic vertebral fracture in patients with end-stage renal disease | Insufficient data reporting |
| 78 | Hauck et al., 2022 | Bisphosphonates and bone mineral density in patients with end-stage kidney disease and renal transplants: A 15-year single-centre experience | Insufficient data reporting |
| 79 | Han et al., 2022 | Lower response to denosumab in diabetes patients on hemodialysis | Insufficient data reporting |
| 80 | Dosogi et al., 2022 | Evaluation of Chronic Kidney Disease-Mineral and Bone Disorder (CKDMBD) Among Patients on twice weekly Hemodialysis in Khartoum Teaching Hospital, Sudan | Insufficient data reporting |
| 81 | Covino et al., 2022 | Hip fracture risk in elderly with non-end-stage chronic kidney disease: a fall related analysis | Insufficient data reporting |
| 82 | Costa et al., 2022 | Hyperuricemia is associated with secondary hyperparathyroidism in patients with chronic kidney disease | Insufficient data reporting |
| 83 | Conley et al., 2022 | Relationship Between Dietary Phosphate Intake and Biomarkers of Bone and Mineral Metabolism in Australian Adults With Chronic Kidney Disease | Insufficient data reporting |
| 84 | Chiang et al., 2022 | Inverse Relationship between Mean Corpuscular Volume and T-Score in Chronic Dialysis Patients | Insufficient data reporting |
| 85 | Campagnaro et al., 2022 | Bone mass measurement by DXA should be interpreted with caution in the CKD population with vascular calcification | Insufficient data reporting |
| 86 | Bittencourt et al., 2022 | Cortical bone density by quantitative computed tomography mirrors disorders of bone structure in bone biopsy of non-dialysis CKD patients | Insufficient data reporting |
| 87 | Aleksova et al., 2022 | DXA-derived advanced hip analysis and the trabecular bone score in end-stage kidney disease secondary to type 1 diabetes | Insufficient data reporting |
| 88 | Al Kalbani et al., 2022 | Females had a worse T-score at lumbar region and males had a worse T-score at femoral region among chronic kidney disease | Insufficient data reporting |
| 89 | Zhu et al., 2021 | Fibroblast growth factor 21 (FGF21) is a sensitive marker of osteoporosis in haemodialysis patients: a cross-sectional observational study | Insufficient data reporting |
| 90 | Yoshioka et al., 2021 | Replacing sedentary time for physical activity on bone density in patients with chronic kidney disease | Insufficient data reporting |
| 91 | Xie et al., 2021 | A retrospective study of end-stage kidney disease patients on maintenance hemodialysis with renal osteodystrophy-associated fragility fractures | Insufficient data reporting |
| 92 | Xiang et al., 2021 | Value of quantitative ultrasound and bioelectrical impedance analysis in detecting low bone mineral density in hemodialysis | Insufficient data reporting |
| 93 | Watanabe et al., 2021 | Home-based exercise and bone mineral density in peritoneal dialysis patients: a randomized pilot study | Insufficient data reporting |
| 94 | Shroff et al., 2020 | Naturally Occurring Stable Calcium Isotope Ratios in Body Compartments Provide a Novel Biomarker of Bone Mineral Balance in Children and Young Adults | Insufficient data reporting |
| 95 | Sethi et al., 2021 | Changing Spectrum of Mineral Bone Disorder in Chronic kidney disease stage 3 to 5 D and Its Associated Factors, A Prospective Cross-Sectional Study from Tertiary Care Hospital in Northern India | Insufficient data reporting |
| 96 | Okada et al., 2021 | Predictors of Bone Mineral Density Improvement after Parathyroidectomy for Secondary Hyperparathyroidism: A Retrospective Single-Center Analysis | Insufficient data reporting |
| 97 | Mizuiri et al., 2021 | Association and predictive value of geriatric nutritional risk index, body composition, or bone mineral density in haemodialysis patients | Insufficient data reporting |
| 98 | Mattera et al., 2021 | Prevalence and Risk Factors for Sarcopenia in Chronic Kidney Disease Patients Undergoing Dialysis: A Cross-Sectional Study | Insufficient data reporting |
| 99 | Kužmová et al., 2021 | Fibroblast Growth Factor 23 and Klotho Are Associated With Trabecular Bone Score but Not Bone Mineral Density in the Early Stages of Chronic Kidney Disease: Results of the Cross-Sectional Study | Insufficient data reporting |
| 100 | Kałużna et al., 2021 | Is Preptin a New Bone Metabolism Parameter in Hemodialysis Patients? | Insufficient data reporting |
| 101 | Iwasaki et al., 2021 | Association Between Bone Mineral Density of the Distal Third of the Radius and Mortality in Patients on Hemodialysis, a Retrospective Cohort Study | Insufficient data reporting |
| 102 | Hori et al., 2021 | Impact of serum magnesium and bone mineral density on systemic fractures in chronic hemodialysis patients | Insufficient data reporting |
| 103 | Chou et al., 2021 | Being elderly is not a contraindication of parathyroidectomy for renal hyperparathyroidism and chronic kidney disease-mineral and bone disorder | Insufficient data reporting |
| 104 | Broadwell et al., 2021 | Denosumab Safety and Efficacy Among Participants in the FREEDOM Extension Study With Mild to Moderate Chronic Kidney Disease | Insufficient data reporting |
| 105 | Abdallah et al., 2021 | The Relationship between Serum Sclerostin Levels and Bone Mineral Disorders and Vascular Calcification in Hemodialysis Patients | Insufficient data reporting |
| 106 | Yun et al., 2020 | Trabecular bone score may indicate chronic kidney disease-mineral and bone disorder (CKD-MBD) phenotypes in hemodialysis patients: a prospective observational study | Insufficient data reporting |
| 107 | Rampersad et al., 2020 | Trabecular bone score in patients with chronic kidney disease | Insufficient data reporting |
| 108 | Ong et al., 2020 | Optimising bone health among older people with hip fractures and co-existing advanced chronic kidney disease | Insufficient data reporting |
| 109 | Malmgren et al., 2020 | Kidney function and its association to imminent, short-and long-term fracture risk—a longitudinal study in older women | Insufficient data reporting |
| 110 | Luo et al., 2020 | Effect of CKD–MBD phenotype on health-related quality of life in patients receiving maintenance hemodialysis: a cross-sectional study | Insufficient data reporting |
| 111 | Lin et al., 2020 | Effects of Chronic Kidney Disease on Hemiarthroplasty Outcomes for Fragility Hip Fracture in Diabetic Patients: A Nationwide Population-Based Observational Study | Insufficient data reporting |
| 112 | Lee et al., 2020 | The impact of protein diet on bone density in people with/without chronic kidney disease: An analysis of the National Health and Nutrition Examination Survey database | Insufficient data reporting |
| 113 | Lamacchia et al., 2020 | Glomerular filtration rate is associated with trabecular bone score in patients with type 2 diabetes mellitus | Insufficient data reporting |
| 114 | Kwon et al., 2020 | Vertebral fracture is associated with myocardial infarction in incident hemodialysis patients: a Korean nationwide population-based study | Insufficient data reporting |
| 115 | Kunizawa et al., 2020 | Denosumab for dialysis patients with osteoporosis: A cohort study | Insufficient data reporting |
| 116 | Hughes-Austin et al., 2020 | Biomarkers of Bone Turnover Identify Subsets of Chronic Kidney Disease Patients at Higher Risk for Fracture | Insufficient data reporting |
| 117 | Desbiens et al., 2020 | Predictive value of quantitative ultrasound parameters in individuals with chronic kidney disease: A population-based analysis of CARTaGENE | Insufficient data reporting |
| 118 | Costa et al., 2020 | Cortical unlike trabecular bone loss is not associated with vascular calcification progression in CKD patients | Insufficient data reporting |
| 119 | Choudhary et al., 2020 | Prevalence of Mineral Bone Disease in Chronic Kidney Disease Patients using Biochemical Markers | Insufficient data reporting |
| 120 | Castro-Alonso et al., 2020 | Prevalence of Vertebral Fractures and Their Prognostic Significance in the Survival in Patients with Chronic Kidney Disease Stages 3-5 Not on Dialysis | Insufficient data reporting |
| 121 | Carbonara et al., 2020 | Renal osteodystrophy and clinical outcomes: data from the Brazilian Registry of Bone Biopsies - REBRABO | Insufficient data reporting |
| 122 | Brunerová et al., 2020 | Loss of bone mineral density and trabecular bone score in elderly hemodialysis patients: a 2-year follow-up, prospective, single-centre study | Insufficient data reporting |
| 123 | Anderson et al., 2020 | Preoperative bone health assessment and optimization in spine surgery | Insufficient data reporting |
| 124 | You et al., 2019 | Prevalence and risk factors for perioperative complications of CKD patients undergoing elective hip surgery | Insufficient data reporting |
| 125 | Xiong et al., 2019 | Association of mineral content outside of bone with coronary artery calcium and 1‐year cardiovascular prognosis in maintenance hemodialysis patients | Insufficient data reporting |
| 126 | Veyrat et al., 2019 | Conservative three-quarter versus subtotal seven-eighths parathyroidectomy in secondary hyperparathyroidism | Insufficient data reporting |
| 127 | Sugimoto et al., 2019 | Efficacy and safety of once-monthly risedronate in osteoporosis subjects with mild-to-moderate chronic kidney disease: a post hoc subgroup analysis of a phase III trial in Japan | Insufficient data reporting |
| 128 | Nigwekar et al., 2019 | Chronic prolonged hyponatremia and risk of hip fracture in elderly patients with chronic kidney disease | Insufficient data reporting |
| 129 | Miyaoka et al., 2019 | Impaired residual renal function predicts denosumab-induced serum calcium decrement as well as increment of bone mineral density in non-severe renal insufficiency | Insufficient data reporting |
| 130 | Liu et al., 2019 | Correlation of the severity of chronic kidney disease with serum inflammation, osteoporosis and vitamin D deficiency | Insufficient data reporting |
| 131 | Li et al., 2019 | Association between renal function and bone mineral density in healthy postmenopausal Chinese women | Insufficient data reporting |
| 132 | Lee et al., 2019 | Serum myostatin levels are associated with abdominal aortic calcification in dialysis patients | Insufficient data reporting |
| 133 | Kuo et al., 2019 | Serum sclerostin levels are positively related to bone mineral density in peritoneal dialysis patients: a cross-sectional study | Insufficient data reporting |
| 134 | Jansz et al., 2020 | The prevalence and incidence of vertebral fractures in end-stage renal disease and the role of parathyroid hormone | Insufficient data reporting |
| 135 | Ho et al., 2019 | Evaluation of the association of Wnt signaling with coronary artery calcification in patients on dialysis with severe secondary hyperparathyroidism | Insufficient data reporting |
| 136 | Han et al., 2019 | Kidney Stones and Risk of Osteoporotic Fracture in Chronic Kidney Disease | Insufficient data reporting |
| 137 | Goto et al., 2019 | Effects of lanthanum carbonate on bone markers and bone mineral density in incident hemodialysis patients | Insufficient data reporting |
| 138 | Desbiens et al., 2019 | Fracture status in middle-aged individuals with early CKD: cross-sectional analysis of the CARTaGENE survey | Insufficient data reporting |
| 139 | Chen et al., 2019 | Free 25-Vitamin D Is Correlated with Cardiovascular Events in Prevalent Hemodialysis Patients but Not with Markers of Renal Mineral Bone Disease | Insufficient data reporting |
| 140 | Tominaga et al., 2018 | Association between bone mineral density, muscle volume, walking ability, and geriatric nutritional risk index in hemodialysis patients | Insufficient data reporting |
| 141 | Ramalho et al., 2018 | The trabecular bone score: Relationships with trabecular and cortical microarchitecture measured by HR-pQCT and histomorphometry in patients with chronic kidney disease | Insufficient data reporting |
| 142 | Przedlacki et al., 2018 | The utility of FRAX® in predicting bone fractures in patients with chronic kidney disease on hemodialysis: a two-year prospective multicenter cohort study | Insufficient data reporting |
| 143 | Pan et al., 2018 | Chronic kidney disease associated with decreased bone mineral density, uric acid and metabolic syndrome | Insufficient data reporting |
| 144 | Nakanishi et al., 2018 | Bone density of the femoral neck in patients on maintenance dialysis | Insufficient data reporting |
| 145 | Le et al., 2018 | Osteoporotic fractures in patients with systemic lupus erythematosus and end stage renal disease | Insufficient data reporting |
| 146 | Jørgensen et al., 2018 | Bioavailable Testosterone Is Positively Associated With Bone Mineral Density in Male Kidney Transplantation Candidates | Insufficient data reporting |
| 147 | Hrnjak et al., 2018 | The effects of extreme low frequency pulsed electromagnetic field on bone mineral density and incidence of fractures in patients with end - stage renal disease on dialysis - three year follow up study | Insufficient data reporting |
| 148 | Fang et al., 2018 | Changes in bone mineral density after total parathyroidectomy without autotransplantation in the end-stage renal disease patients with secondary hyperparathyroidism | Insufficient data reporting |
| 149 | Dusceac et al., 2018 | Chronic hemodialysis is associated with lower trabecular bone score, independent of bone mineral density: a case-control study | Insufficient data reporting |
| 150 | Chen et al., 2018 | Association of renal function with bone mineral density and fracture risk in the Longitudinal Aging Study Amsterdam | Insufficient data reporting |
| 151 | Chao et al., 2018 | Chronic kidney disease predicts a lower probability of improvement in patient-reported experience measures among patients with fractures: a prospective multicenter cohort study | Insufficient data reporting |
| 152 | Brunerová et al., 2018 | Predictors of bone fractures in a single-centre cohort of hemodialysis patients: a 2-year follow-up study | Insufficient data reporting |
| 153 | Aleksova et al., 2018 | Sex hormone–binding globulin is a biomarker associated with nonvertebral fracture in men on dialysis therapy | Insufficient data reporting |
| 154 | Aggarwal et al., 2018 | Effects of Short Term Alendronate Administration on Bone Mineral Density in Patients with Chronic Kidney Disease | Insufficient data reporting |
| 155 | Zhang et al., 2017 | Effect of lanthanum carbonate on coronary artery calcification and bone mineral density in maintenance hemodialysis patients with diabetes complicated with adynamic bone disease: A prospective pilot study | Insufficient data reporting |
| 156 | Shigematsu et al., 2017 | Risedronate therapy in patients with mild-to-moderate chronic kidney disease with osteoporosis: post-hoc analysis of data from the risedronate phase III clinical trials | Insufficient data reporting |
| 157 | Pérez-Sáez et al., 2017 | Bone density, microarchitecture, and material strength in chronic kidney disease patients at the time of kidney transplantation | Insufficient data reporting |
| 158 | Marinho et al., 2017 | Effect of a resistance exercise training program on bone markers in hemodialysis patients | Insufficient data reporting |
| 159 | Li et al., 2017 | Association of CKD-MBD Markers with All-Cause Mortality in Prevalent Hemodialysis Patients: A Cohort Study in Beijing | Insufficient data reporting |
| 160 | Jørgensen et al., 2017 | Bone turnover markers are associated with bone density, but not with fracture in end stage kidney disease: a cross-sectional study | Insufficient data reporting |
| 161 | Fournie et al., 2017 | The Relationship Between Body Composition and Bone Quality Measured with HR-pQCT in Peritoneal Dialysis Patients | Insufficient data reporting |
| 162 | Cheng et al., 2017 | Young patients and those with a low eGFR benefitted more from denosumab therapy in femoral neck bone mineral density | Insufficient data reporting |
| 163 | Carvalho et al., 2017 | Cortical bone analysis in a predialysis population: a comparison with a dialysis population | Insufficient data reporting |
| 164 | Bielesz et al., 2017 | Cortical porosity not superior to conventional densitometry in identifying hemodialysis patients with fragility fracture | Insufficient data reporting |
| 165 | Bala et al., 2017 | Biochemical markers of mineral bone disorder in South African patients on maintenance haemodialysis | Insufficient data reporting |
| 166 | Atteritano et al., 2017 | Higher serum sclerostin levels and insufficiency of vitamin D are strongly associated with vertebral fractures in hemodialysis patients: a case control study | Insufficient data reporting |
| 167 | Aleksova et al., 2018 | The Trabecular Bone Score is associated with bone mineral density, markers of bone turnover and prevalent fracture in patients with chronic kidney disease stages 5 and 5D | Insufficient data reporting |
| 168 | Zhou et al., 2016 | Mineral and bone disorder and its association with cardiovascular parameters in Chinese patients with chronic kidney disease | Insufficient data reporting |
| 169 | Shaheen et al., 2016 | Multinational observational study on clinical practices and therapeutic management of mineral and bone disorders in patients with chronic kidney disease stages 4, 5, and 5D: The OCEANOS study | Insufficient data reporting |
| 170 | Sakaguchi et al., 2016 | Association between Density of Coronary Artery Calcification and Serum Magnesium Levels among Patients with Chronic Kidney Disease | Insufficient data reporting |
| 171 | Nishikawa et al., 2016 | Safety and effectiveness of daily teriparatide for osteoporosis in patients with severe stages of chronic kidney disease: post hoc analysis of a postmarketing observational study | Insufficient data reporting |
| 172 | Naylor et al., 2016 | Trabecular Bone Score and Incident Fragility Fracture Risk in Adults with Reduced Kidney Function | Insufficient data reporting |
| 173 | Mohamed et al., 2016 | Correlation of serum sclerostin levels and bone mineral density and vascular calcification in hemodialysis egyptian patients | Insufficient data reporting |
| 174 | Kim et al., 2016 | Association of Serum Osteoprotegerin Levels with Bone Loss in Chronic Kidney Disease: Insights from the KNOW-CKD Study | Insufficient data reporting |
| 175 | Keronen et al., 2016 | Bone histomorphometry and indicators of bone and mineral metabolism in wait-listed dialysis patients | Insufficient data reporting |
| 176 | Chen et al., 2016 | Vertebral bone density associates with coronary artery calcification and is an independent predictor of poor outcome in end-stage renal disease patients | Insufficient data reporting |
| 177 | Cheng et al., 2017 | Can Lumbar Spine Bone Mineral Density Predict Readmission in Denosumab-Treated Chronic Kidney Disease Patients? | Insufficient data reporting |
| 178 | Căpuşă et al., 2016 | THE PREVALENCE OF BIOCHEMICAL ABNORMALITIES OF CHRONIC KIDNEY DISEASE. MINERAL AND BONE DISORDERS IN UNTREATED NON-DIALYSIS PATIENTS A MULTICENTER STUDY | Insufficient data reporting |
| 179 | Auguste et al., 2016 | Conventional hemodialysis is associated with greater bone loss than nocturnal hemodialysis: a retrospective observational study of a convenience cohort | Insufficient data reporting |
| 180 | Okoye et al., 2015 | Prevalence of CKD-MBD in pre-dialysis patients using biochemical markers in Enugu, South-East Nigeria | Insufficient data reporting |
| 181 | Negri et al., 2012 | Evaluation of bone microarchitecture by high-resolution peripheral quantitative computed tomography in patients with chronic kidney disease: Comparison with transiliac bone biopsy | Insufficient data reporting |
| 182 | Sandra et al., 2015 | Association Between Body Composition and Bone Mineral Density in Men on Hemodialysis | Insufficient data reporting |
| 183 | Malluche et al., 2015 | High parathyroid hormone level and osteoporosis predict progression of coronary artery calcification in patients on dialysis | Insufficient data reporting |
| 184 | Kuipers et al., 2015 | Renal Function and Bone Loss in a Cohort of Afro-Caribbean Men | Insufficient data reporting |
| 185 | Jorgensen et al., 2015 | Bone turnover markers are notassociated with bone density or fragility fractures in renal transplant candidates | Insufficient data reporting |
| 186 | Chen et al., 2015 | Effects of Denosumab and Calcitriol on Severe Secondary Hyperparathyroidism in Dialysis Patients With Low Bone Mass | Insufficient data reporting |
| 187 | Saafan et al., 2014 | Relation between Hyperparathyroidism and Osteoporosis in Chronic Renal Failure Patients with Regular Haemodialysis | Insufficient data reporting |
| 188 | Morishita et al., 2014 | Positive association of vigorous and moderate physical activity volumes with skeletal muscle mass but not bone density or metabolism markers in hemodialysis patients | Insufficient data reporting |
| 189 | Maravic et al., 2014 | Incidence and risk factors for hip fractures in dialysis patients | Insufficient data reporting |
| 190 | Haghverdi et al., 2014 | Effect of raloxifene on parathyroid hormone in osteopenic and osteoporotic postmenopausal women with chronic kidney disease stage 5 | Insufficient data reporting |
| 191 | Chen et al., 2014 | Greater risk of hip fracture in hemodialysis than in peritoneal dialysis | Insufficient data reporting |
| 192 | Soyupek et al., 2013 | The upper extremity musculoskeletal complications in dialysis patients: Comparison between hemodialysis and peritoneal dialysis | Insufficient data reporting |
| 193 | Park et al., 2013 | Determinants and survival implications of low bone mineral density in end-stage renal disease patients | Insufficient data reporting |
| 194 | Nybo et al., 2013 | Determinants of bone mineral density in patients on haemodialysis or peritoneal dialysis - a cross-sectional, longitudinal study | Insufficient data reporting |
| 195 | Nickolas et al., 2013 | Rapid cortical bone loss in patients with chronic kidney disease | Insufficient data reporting |
| 196 | Fusaro et al., 2013 | High Prevalence of Vertebral Fractures Assessed by Quantitative Morphometry in Hemodialysis Patients, Strongly Associated with Vascular Calcifications | Insufficient data reporting |
| 197 | Choi et al., 2013 | Association of Bone Mineral Density with Albuminuria and Estimated Glomerular Filtration Rate : The Dong-gu Study | Insufficient data reporting |
| 198 | BAKAN et al., 2013 | The osteoporotic effect of arteriovenous fistula on the ipsilateral upper extremity in hemodialysis patients | Insufficient data reporting |
| 199 | Amemiya et al., 2013 | Association between high-molecular-weight adiponectin and bone mineral density in hemodialysis patients | Insufficient data reporting |
| 200 | Al-Modeer et al., 2013 | Profile of morbidity among elderly at home health care service in Southern Saudi Arabia | Insufficient data reporting |
| 201 | Almirall et al., 2013 | Mineral and bone disease in black african hemodialysis patients | Insufficient data reporting |
| 202 | Ahmadi et al., 2013 | Relationship between serum leptin levels and bone mineral density and bone metabolic markers in patients on hemodialysis | Insufficient data reporting |
| 203 | Thambiah et al., 2012 | Circulating sclerostin and Dickkopf-1 (DKK1) in predialysis chronic kidney disease (CKD): relationship with bone density and arterial stiffness | Insufficient data reporting |
| 204 | Pelletier et al., 2012 | Bone microarchitecture is more severely affected in patients on hemodialysis than in those receiving peritoneal dialysis | Insufficient data reporting |
| 205 | Okuno et al., 2012 | Serum adiponectin and bone mineral density in male hemodialysis patients | Insufficient data reporting |
| 206 | Mitsopoulos et al., 2012 | Impact of long-term cinacalcet, ibandronate or teriparatide therapy on bone mineral density of hemodialysis patients: a pilot study | Insufficient data reporting |
| 207 | Lu et al., 2012 | Bone turnover markers predict changes in bone mineral density after parathyroidectomy in patients with renal hyperparathyroidism | Insufficient data reporting |
| 208 | Kang et al., 2012 | Low-calcium dialysate as a risk factor for decline in bone mineral density in peritoneal dialysis patients | Insufficient data reporting |
| 209 | Jamal et al., 2012 | Bone mineral density by DXA and HR pQCT can discriminate fracture status in men and women with stages 3 to 5 chronic kidney disease | Insufficient data reporting |
| 210 | Iimori et al., 2012 | Diagnostic usefulness of bone mineral density and biochemical markers of bone turnover in predicting fracture in CKD stage 5D patients-a single-center cohort study | Insufficient data reporting |
| 211 | Desjardins et al., 2012 | FGF23 is independently associated with vascular calcification but not bone mineral density in patients at various CKD stages | Insufficient data reporting |
| 212 | Chavlovski et al., 2012 | Retrospective review of bone mineral metabolism management in end-stage renal disease patients wait-listed for renal transplant | Insufficient data reporting |
| 213 | Bosworth et al., 2011 | The effect of combined calcium and cholecalciferol supplementation on bone mineral density in elderly women with moderate chronic kidney disease | Insufficient data reporting |
| 214 | Bogoch et al., 2012 | Secondary Causes of Osteoporosis in Fracture Patients | Insufficient data reporting |
| 215 | Osorio et al., 2011 | Mineral-bone metabolism markers in young hemodialysis patients | Insufficient data reporting |
| 216 | Manghat et al., 2011 | Association of bone turnover markers and arterial stiffness in pre-dialysis chronic kidney disease (CKD) | Insufficient data reporting |
| 217 | Ishimura et al., 2011 | Effect of cinacalcet on bone mineral density of the radius in hemodialysis patients with secondary hyperparathyroidism | Insufficient data reporting |
| 218 | Filgueira et al., 2011 | Is coronary artery calcification associated with vertebral bone density in nondialyzed chronic kidney disease patients? | Insufficient data reporting |
| 219 | Ambrus et al., 2011 | Vitamin D insufficiency and bone fractures in patients on maintenance hemodialysis | Insufficient data reporting |
| 220 | Toussaint et al., 2010 | Using vertebral bone densitometry to determine aortic calcification in patients with chronic kidney disease | Insufficient data reporting |
| 221 | Sanusi et al., 2010 | Prevalence and pattern of renal bone disease in end stage renal disease patients in Ile-Ife, Nigeria | Insufficient data reporting |
| 222 | Park et al., 2010 | The relationship between circulating fibroblast growth factor 23 and bone metabolism factors in Korean hemodialysis patients | Insufficient data reporting |
| 223 | Park et al., 2010 | Association of serum alkaline phosphatase and bone mineral density in maintenance hemodialysis patients | Insufficient data reporting |
| 224 | Nickolas et al., 2010 | Bone mass and microarchitecture in CKD patients with fracture | Insufficient data reporting |
| 225 | Manghat et al., 2010 | Fibroblast growth factor-23 is associated with C-reactive protein, serum phosphate and bone mineral density in chronic kidney disease | Insufficient data reporting |
| 226 | Kaji et al., 2010 | Mild renal dysfunction is a risk factor for a decrease in bone mineral density and vertebral fractures in Japanese postmenopausal women | Insufficient data reporting |
| 227 | Kadiroglu et al., 2010 | The frequency of osteoporosis in hemodialysis and continuous ambulatory peritoneal dialysis patients according to PTH levels after active vitamin D therapy during the two years period | Insufficient data reporting |
| 228 | Cejka et al., 2010 | Bone microstructure and volumetric density in hemodialysis patients: assessed by high-resolution peripheral quantitative computed tomography | Insufficient data reporting |
| 229 | Yilmaz et al., 2009 | Leptin and Bone Mineral Density in Haemodialysis Patients | Insufficient data reporting |
| 230 | Mares et al., 2009 | Determinants of prevalent vertebral fractures and progressive bone loss in long-term hemodialysis patients | Insufficient data reporting |
| 231 | Maeno et al., 2009 | Significant association of fracture of the lumbar spine with mortality in female hemodialysis patients: a prospective observational study | Insufficient data reporting |
| 232 | Lee et al., 2009 | Association between C-reactive protein and biomarkers of bone and mineral metabolism in chronic hemodialysis patients: a cross-sectional study | Insufficient data reporting |
| 233 | Kirkpantur et al., 2009 | Association among serum fetuin-A level, coronary artery calcification, and bone mineral densitometry in maintenance hemodialysis patients | Insufficient data reporting |
| 234 | Kirkpantur et al., 2009 | Proton pump inhibitor omeprazole use is associated with low bone mineral density in maintenance haemodialysis patients | Insufficient data reporting |
| 235 | Grzegorzewska et al., 2009 | Total body mass is better than body mass index as a prognostic parameter for bone mineral density in dialyzed patients | Insufficient data reporting |
| 236 | Fahrleitner-Pammer et al., 2009 | The effect of RANKL and OPG on bone mineral density in pre-dialysis chronic renal failure | Insufficient data reporting |
| 237 | Castillo et al., 2009 | Relation between body mass index and bone mineral density among haemodialysis patients with chronic kidney disease | Insufficient data reporting |
| 238 | Bouchard et al., 2009 | Comparison of the prevalence of calcidiol insufficiency in predialysis and osteoporotic populations | Insufficient data reporting |
| 239 | Torres et al., 2008 | Bone mass does not correlate with the serum fibroblast growth factor 23 in hemodialysis patients | Insufficient data reporting |
| 240 | Mohamed et al., 2008 | Bone densitometric analysis in Egyptian hemodialysis patients | Insufficient data reporting |
| 241 | Matsubara et al., 2008 | Bone mineral density in end-stage renal disease patients: association with wasting, cardiovascular disease and mortality | Insufficient data reporting |
| 242 | Doumouchtsis et al., 2008 | The effect of sexual hormone abnormalities on proximal femur bone mineral density in hemodialysis patients and the possible role of RANKL | Insufficient data reporting |
| 243 | Donadio et al., 2008 | Single- and multi-frequency bioelectrical impedance analyses to analyse body composition in maintenance haemodialysis patients: comparison with dual-energy x-ray absorptiometry | Insufficient data reporting |
| 244 | Chou et al., 2008 | Parathyroidectomy followed by kidney transplantation can improve bone mineral density in patients with secondary hyperparathyroidism | Insufficient data reporting |
| 245 | Bergua et al., 2008 | Effect of Cinacalcet on hypercalcemia and bone mineral density in renal transplanted patients with secondary hyperparathyroidism | Insufficient data reporting |
| 246 | Adragao et al., 2008 | Bone mineral density, vascular calcifications, and arterial stiffness in peritoneal dialysis patients | Insufficient data reporting |
| 247 | Raggi et al., 2007 | Pulse wave velocity is inversely related to vertebral bone density in hemodialysis patients | Insufficient data reporting |
| 248 | Obatake et al., 2007 | Annual change in bone mineral density in predialysis patients with chronic renal failure: significance of a decrease in serum 1, 25-dihydroxy-vitamin D | Insufficient data reporting |
| 249 | Miller et al., 2007 | Teriparatide in postmenopausal women with osteoporosis and mild or moderate renal impairment | Insufficient data reporting |
| 250 | Hernandes et al., 2007 | Evaluation of the role of severe hyperparathyroidism on coronary artery calcification in dialysis patients | Insufficient data reporting |
| 251 | Elder et al., 2007 | Vitamin D levels, bone turnover and bone mineral density show seasonal variation in patients with chronic kidney disease stage 5 | Insufficient data reporting |
| 252 | Nakashima et al., 2006 | Osteoprotegerin and bone mineral density in hemodialysis patients | Insufficient data reporting |
| 253 | Jamal et al., 2006 | Impaired muscle strength is associated with fractures in hemodialysis patients | Insufficient data reporting |
| 254 | Elder et al., 2006 | 25-Hydroxyvitamin D deficiency and diabetes predict reduced BMD in patients with chronic kidney disease | Insufficient data reporting |
| 255 | Buargub et al., 2006 | Prevalence and pattern of renal osteodystrophy in chronic hemodialysis patients: a cross sectional study of 103 patients | Insufficient data reporting |
| 256 | Barreto et al., 2006 | Osteoporosis in hemodialysis patients revisited by bone histomorphometry: A new insight into an old problem | Insufficient data reporting |
| 257 | Wetmore et al., 2005 | Effects of short-term alendronate on bone mineral density in haemodialysis patients | Insufficient data reporting |
| 258 | Okuno et al., 2005 | Serum levels of C-terminal telopeptide of type I collagen: a useful new marker of cortical bone loss in hemodialysis patients | Insufficient data reporting |
| 259 | Negri et al., 2005 | Relationship between weight, body composition, and bone mass in peritoneal dialysis | Insufficient data reporting |
| 260 | Mucsi et al., 2005 | Serum 25 (OH)-vitamin D levels and bone metabolism in patients on maintenance hemodialysis | Insufficient data reporting |
| 261 | Crisafulli et al., 2005 | Osteoprotegerin and bone mineral density in hemodiafiltration patients | Insufficient data reporting |
| 262 | Cauley et al., 2005 | Factors associated with the lumbar spine and proximal femur bone mineral density in older men | Insufficient data reporting |
| 263 | Lobao et al., 2004 | High prevalence of low bone mineral density in pre-dialysis chronic kidney disease patients: bone histomorphometric analysis | Insufficient data reporting |
| 264 | Hasegawa et al., 2004 | Estimation of bone mineral density and architectural parameters of the distal radius in hemodialysis patients using peripheral quantitative computed tomography | Insufficient data reporting |
| 265 | Yano et al., 2003 | Effect of parathyroidectomy on bone mineral density in hemodialysis patients with secondary hyperparathyroidism: possible usefulness of preoperative determination of parathyroid hormone level for prediction of bone regain | Insufficient data reporting |
| 266 | Nakashima et al., 2003 | Radial bone mineral density in hemodialysis patients with adynamic bone disease | Insufficient data reporting |
| 267 | Kaji et al., 2003 | Factors affecting bone mineral density in hemodialysis patients with diabetic nephropathy | Insufficient data reporting |
| 268 | Groothoff et al., 2003 | Severe bone disease and low bone mineral density after juvenile renal failure | Insufficient data reporting |
| 269 | Ghazali et al., 2003 | Bone mineral density directly correlates with elevated serum leptin in haemodialysis patients | Insufficient data reporting |
| 270 | Nakashima et al., 2003 | Bone mineral density may be related to atherosclerosis in hemodialysis patients with non diabetic nephropathy | Insufficient data reporting |
| 271 | Haas et al., 2002 | Osteoprotegerin and parathyroid hormone as markers of high-turnover osteodystrophy and decreased bone mineralization in hemodialysis patients | Insufficient data reporting |
| 272 | Gohda et al., 2002 | An (CA) n dinucleotide repeat polymorphism of the interleukin-6 (IL-6) gene is associated with metacarpal bone mineral density in hemodialysis patients | Insufficient data reporting |
| 273 | Balon et al., 2002 | Bone mineral density in patients beginning hemodialysis treatment | Insufficient data reporting |
| 274 | Lai et al., 2001 | Effect of low molecular weight heparin on bone metabolism and hyperlipidemia in patients on maintenance hemodialysis | Insufficient data reporting |
| 275 | Zivicnjak et al., 2000 | Does growth hormone therapy harmonize distorted morphology and body composition in chronic renal failure? | Insufficient data reporting |
| 276 | Torregrosa et al., 2000 | Vitamin D receptor gene polymorphisms and bone mineral density in patients on hemodialysis | Insufficient data reporting |
| 277 | Peretz et al., 2000 | Quantitative ultrasound and dual X-ray absorptiometry measurements of the calcaneus in patients on maintenance hemodialysis | Insufficient data reporting |
| 278 | Arici et al., 2000 | Bone mineral density in haemodialysis patients: A comparative study of dual-energy X-ray absorptiometry and quantitative ultrasound | Insufficient data reporting |
| 279 | Lacativa et al., 2005 | Risk Factors for Decreased Total Body and Regional Bone Mineral Density in Hemodialysis Patients With Severe Secondary Hyperparathyroidism | Non-compliance with inclusion criteria |
| 280 | Rivadeneira et al., 2005 | Prevalence and risk factors of osteoporosis in haemodiaylisis | Non-compliance with inclusion criteria |
| 281 | Barratt et al., 2020 | Correlation between Dialysis Vintage and Vitamin D and Bone Mass Density on Patients with Chronic Kidney Disease Undergoing Hemodialysis In Zainoel Abidin Hospital | Non-compliance with inclusion criteria |
| 282 | Abdelsattar et al., 2024 | Serum osteocalcin concentration as a biomarker of osteoporosis in Egyptian patients with chronic renal failure on regular hemodialysis | Non-compliance with inclusion criteria |
| 283 | Hoffmann et al., 2016 | Vitamin D Status and Bone Mineral Density is Influenced by Vitamin D Supplementation and Vitamin K1 Intake in Adults with Diabetes and Chronic Kidney Disease | Non-compliance with inclusion criteria |
| 284 | El-Husseini et al., 2022 | Low Turnover Renal Osteodystrophy With Abnormal Bone Quality and Vascular Calcification in Patients With Mild-to-Moderate CKD | Non-compliance with inclusion criteria |
| 285 | Nickolas et al., 2006 | Relationship between Moderate to Severe Kidney Disease and Hip Fracture in the United States | Non-compliance with inclusion criteria |
| 286 | Rigalleau et al., 2007 | Bone loss in diabetic patients with chronic kidney disease | Non-compliance with inclusion criteria |
| 287 | Chang et al., 2016 | Evaluation of three risk assessment tools in discriminating fracture status among Chinese patients undergoing hemodialysis | Non-compliance with inclusion criteria |
| 288 | Jeong et al., 2017 | Differentially expressed miR-3680-5p is associated with parathyroid hormone regulation in peritoneal dialysis patients | Non-compliance with inclusion criteria |
| 289 | Ozdemir et al., 2010 | Evaluation of Bone Mineral Density in Hemodialysis Patients | Non-compliance with inclusion criteria |
| 290 | Mandıroğlu et al., 2013 | The Evaluation of Renal Osteodystrophy in Patients on Hemodialysis by Biochemical and Radiological Methods | Non-compliance with inclusion criteria |
| 291 | Mohamed et al., 2020 | The Role of Dexa Scan Application in Uremic Patients on Hemodialysis in Ibn-Sina Hemodialysis Center | Non-compliance with inclusion criteria |
| 292 | Paranhos-Neto et al., 2018 | HR-pQCT detects alterations in bone microstructure in men with CKD stages 3 and 4, which are influenced by hormonal changes and body composition | Non-compliance with inclusion criteria |
| 293 | Sanoesan et al., 2024 | Bone turnover prediction in patients with chronic kidney disease (CKD) undergoing hemodialysis using shortened dynamic 18F‑NaF PET/CT Ki–Patlak | Non-compliance with inclusion criteria |
| 294 | Trombetti et al., 2013 | Alterations of bone microstructure and strength in end-stage renal failure | Non-compliance with inclusion criteria |
| 295 | Wang et al., 2022 | Combination of Quantitative Computed Tomography and Blood Biochemistry for Evaluating the Relationship Between Nutrition and Bone Mineral Density in Patients on Maintenance Hemodialysis | Non-compliance with inclusion criteria |
| 296 | Wu et al., 2024 | An Automated Assessment Method for Chronic Kidney Disease–Mineral and Bone Disorder (CKD-MBD) Utilizing Metacarpal Cortical Percentage | Non-compliance with inclusion criteria |
| 297 | Zhan et al., 2023 | Changes in Bone Mineral Density and Related Influencing Factors Assessed by Quantitative Computed Tomography in Maintenance Dialysis Patients | Non-compliance with inclusion criteria |
| 298 | Eid Mohamed et al., 2022 | Prevalence and severity of mineral bone disorders in chronic kidney disease patients | Non-compliance with inclusion criteria |
| 299 | Nakano et al., 2024 | Effect of osteosarcopenia on longitudinal mortality risk and chronic kidney  disease progression in older adults | Non-compliance with inclusion criteria |
| 300 | Yilmaz et al., 2015 | FGF-23, α-Klotho Gene Polymorphism and Their Relationship with the Markers of Bone Metabolism in Chronic Peritoneal Dialysis Patients | Non-compliance with inclusion criteria |
| 301 | Govindarajan et al., 2011 | Bone mineral density in patients with end-stage renal disease and its evolution after kidney transplantation | Non-compliance with inclusion criteria |
| 302 | Dolgos et al., 2010 | Osteoporosis is a prevalent finding in patients with solid organ failure awaiting transplantation – a population based study | Non-compliance with inclusion criteria |
| 303 | Jørgensen et al., 2016 | Effect of Intravenous Contrast on Volumetric Bone Mineral Density in Patients with Chronic Kidney Disease | Non-compliance with inclusion criteria |
| 304 | Torregrosa et al., 2003 | Usefulness of pamidronate in severe secondary hyperparathyroidism in patients undergoing hemodialysis | Non-compliance with inclusion criteria |
| 305 | Zosin et al., 2005 | CROSS SECTIONAL DATA IN RENAL OSTEODYSTROPHY | Non-compliance with inclusion criteria |
| 306 | Montenegro et al., 2024 | Changes in bone mineral density in patients with non-dialysis-dependent chronic kidney disease are associated with body composition | Non-compliance with inclusion criteria |
| 307 | Kumar et al., 2023 | Correlation of vitamin D and bone mineral density in diabetic patients with chronic renal disease: A single institute study | Non-compliance with inclusion criteria |
| 308 | Kao et al., 2023 | Effects of osteoporosis medications on bone fracture in patients with chronic kidney disease | Non-compliance with inclusion criteria |
| 309 | Lee et al., 2022 | Association between depressive symptoms and bone density in elderly patients with non-dialysis dependent chronic kidney disease | Non-compliance with inclusion criteria |
| 310 | Huang et al., 2022 | The predictive value of coronary artery calcification score combined with bone mineral density for the 2-year risk of cardiovascular events in maintenance hemodialysis patients | Non-compliance with inclusion criteria |
| 311 | Xu et al., 2021 | Secondary hyperparathyroidism and adverse health outcomes in adults with chronic kidney disease | Non-compliance with inclusion criteria |
| 312 | Druck et al., 2019 | Osteopontin Levels in Patients With Chronic Kidney Disease Stage 5 on Hemodialysis Directly Correlate With Intact Parathyroid Hormone and Alkaline Phosphatase | Non-compliance with inclusion criteria |
| 313 | Santo Ismoyo et al., 2018 | Risk Factors for Impaired Bone Density in Male Patients Underwent Hemodialysis Due to Chronic Kidney Disease | Non-compliance with inclusion criteria |
| 314 | Chang et al., 2016 | Prevalence of low bone mineral density in patients undergoing maintenance hemodialysis and relevant factors | Non-compliance with inclusion criteria |
| 315 | Calza et al., 2016 | Improvement in renal function and bone mineral density after a switch from tenofovir/emtricitabine plus ritonavir-boosted protease inhibitor to raltegravir plus nevirapine: a pilot study | Non-compliance with inclusion criteria |
| 316 | Ambrus et al., 2011 | Bone mineral density and parathyroid function in patients on maintenance hemodialysis | Non-compliance with inclusion criteria |
| 317 | Özdemir et al., 2010 | Evaluation of bone mineral density in hemodialysis patients | Non-compliance with inclusion criteria |
| 318 | Briet et al., 2010 | Age-independent association between arterial and bone remodeling in mild-to-moderate chronic kidney disease | Non-compliance with inclusion criteria |
| 319 | Bacchetta et al., 2010 | Early impairment of trabecular microarchitecture assessed with HR-pQCT in patients with stage II-IV chronic kidney disease | Non-compliance with inclusion criteria |
| 320 | Hernández et al., 2003 | Effects of raloxifene on bone metabolism and serum lipids in postmenopausal women on chronic hemodialysis | Non-compliance with inclusion criteria |
| 321 | Asai et al., 2001 | Collagen type Iα1 gene polymorphism may be associated with the rate of bone mineral density decrease in female hemodialyzed patients | Non-compliance with inclusion criteria |
| 322 | Lu et al., 2023 | Serum Osteoprotegerin Level Is Negatively Associated with Bone Mineral Density in Patients Undergoing Maintenance Hemodialysis | Non-compliance with inclusion criteria |
| 323 | Grzegorzewska et al., 2007 | Influence of Age and Sex on Bone Mineral Density in Dialysis Patients | Non-compliance with inclusion criteria |
| 324 | Lu et al., 2022 | Serum Sclerostin Level Is Negatively Associated with Bone Mineral Density in Hemodialysis Patients | Non-compliance with inclusion criteria |
| 325 | Toussaint et al., 2008 | Associations between vascular calcification, arterial stiffness and bone mineral density in chronic kidney disease | Non-compliance with inclusion criteria |

**eTable 2.** Risk of bias assessment of the studies according to the JBI checklist.

| **Author/Year** | **Type of study** | **Item 1** | **Item 2** | **Item 3** | **Item 4** | **Item 5** | **Item 6** | **Item 7** | **Item 8** | **Item 9** | **Total** |
| --- | --- | --- | --- | --- | --- | --- | --- | --- | --- | --- | --- |
| Afifi et al., 2019 [[1](#_ENREF_1)] | Cross-sectional | 0 | 0 | 0 | 1 | 1 | 1 | 1 | NA | 1 | Medium |
| Aggarwal et al., 2013 [[2](#_ENREF_2)] | Cross-sectional | 1 | 0 | 0 | 1 | 1 | 1 | 1 | NA | 1 | Medium |
| Aghighi et al., 2017 [[3](#_ENREF_3)] | Cross-sectional | 1 | 0 | 0 | 1 | 1 | 1 | 1 | NA | 1 | Medium |
| Akkupalli et al., 2013 [[4](#_ENREF_4)] | Cross-sectional | 0 | 0 | 0 | 1 | 1 | 1 | 1 | NA | 1 | Medium |
| Amirkhanlou et al., 2021 [[5](#_ENREF_5)] | Cross-sectional | 0 | 0 | 1 | 1 | 1 | 1 | 1 | NA | 1 | Medium |
| Anwar et al., 2021 [[6](#_ENREF_6)] | Cross-sectional | 0 | 0 | 0 | 1 | 1 | 1 | 1 | NA | 1 | Medium |
| Bezerra de Carvalho et al., 2019 [[7](#_ENREF_7)] | Cohort | 1 | 1 | 1 | 1 | 1 | 1 | 1 | NA | 1 | Low |
| Binici et al., 2010 [[8](#_ENREF_8)] | Cross-sectional | 1 | 0 | 0 | 1 | 1 | 1 | 1 | NA | 1 | Medium |
| Blomquist et al., 2016 [[9](#_ENREF_9)] | Cross-sectional | 1 | 0 | 0 | 0 | 1 | 1 | 1 | NA | 1 | Medium |
| Bouksila et al., 2019 [[10](#_ENREF_10)] | Cross-sectional | 1 | 0 | 0 | 0 | 1 | 1 | 1 | NA | 1 | Medium |
| Brunerová et al., 2016 [[11](#_ENREF_11)] | Cross-sectional | 1 | 0 | 0 | 0 | 1 | 1 | 1 | NA | 1 | Medium |
| Chen et al., 2019 [[12](#_ENREF_12)] | Cross-sectional | 1 | 0 | 1 | 1 | 1 | 1 | 1 | NA | 1 | Low |
| Chi et al., 2022 [[13](#_ENREF_13)] | Cross-sectional | 0 | 0 | 0 | 0 | 1 | 1 | 1 | NA | 1 | High |
| Chue et al., 2012 [[14](#_ENREF_14)] | Cross-sectional | 1 | 0 | 0 | 1 | 1 | 1 | 1 | NA | 1 | Medium |
| Dave et al., 2015 [[15](#_ENREF_15)] | Cross-sectional | 0 | 0 | 0 | 1 | 1 | 1 | 1 | NA | 1 | Medium |
| Davenport et al., 2022 [[16](#_ENREF_16)] | Cross-sectional | 0 | 0 | 1 | 1 | 1 | 1 | 1 | NA | 1 | Medium |
| Davenport et al., 2023 [[17](#_ENREF_17)] | Cross-sectional | 1 | 1 | 1 | 1 | 1 | 1 | 1 | NA | 1 | Low |
| Davina et al., 2017 [[18](#_ENREF_18)] | Cross-sectional | 0 | 0 | 0 | 0 | 1 | 1 | 1 | NA | 1 | High |
| Demir et al., 2023 [[19](#_ENREF_19)] | Cross-sectional | 0 | 0 | 0 | 0 | 1 | 1 | 1 | NA | 1 | High |
| Disthabanchong et al., 2014 [[20](#_ENREF_20)] | Cohort | 1 | 0 | 0 | 1 | 1 | 1 | 1 | NA | 1 | Medium |
| Dogan et al., 2023 [[21](#_ENREF_21)] | Cross-sectional | 0 | 0 | 0 | 1 | 1 | 1 | 1 | NA | 1 | Medium |
| Doumouchtsis et al., 2008 [[22](#_ENREF_22)] | Cross-sectional | 1 | 0 | 0 | 0 | 1 | 1 | 1 | NA | 1 | Medium |
| Ersoy et al., 2006 [[23](#_ENREF_23)] | Cross-sectional | 1 | 1 | 1 | 1 | 1 | 1 | 1 | NA | 1 | Low |
| Fidan et al., 2016 [[24](#_ENREF_24)] | Cross-sectional | 1 | 0 | 0 | 1 | 1 | 1 | 1 | NA | 1 | Medium |
| Filon et al., 2019 [[25](#_ENREF_25)] | Cross-sectional | 1 | 0 | 0 | 0 | 1 | 1 | 1 | NA | 1 | Medium |
| Gomez-Islas et al., 2020 [[26](#_ENREF_26)] | Cohort | 0 | 1 | 1 | 1 | 1 | 1 | 1 | NA | 1 | Low |
| Grzegorzewska et al., 2008 [[27](#_ENREF_27)] | Cross-sectional | 1 | 0 | 0 | 0 | 1 | 1 | 1 | NA | 1 | Medium |
| Huang et al., 2009 [[28](#_ENREF_28)] | Cross-sectional | 1 | 0 | 0 | 1 | 1 | 1 | 1 | NA | 1 | Medium |
| Huang et al., 2015 [[29](#_ENREF_29)] | Cross-sectional | 1 | 0 | 0 | 0 | 1 | 1 | 1 | NA | 1 | Medium |
| Huang et al., 2024 [[30](#_ENREF_30)] | Cross-sectional | 0 | 0 | 0 | 1 | 1 | 1 | 1 | NA | 1 | Medium |
| Hussain et al., 2023 [[31](#_ENREF_31)] | Cross-sectional | 0 | 0 | 0 | 0 | 1 | 1 | 1 | NA | 1 | High |
| Haarhaus et al., 2009 [[32](#_ENREF_32)] | Cross-sectional | 0 | 1 | 0 | 1 | 1 | 1 | 1 | NA | 1 | Medium |
| Hyun et al., 2020 [[33](#_ENREF_33)] | Cohort | 1 | 1 | 1 | 1 | 1 | 1 | 1 | NA | 1 | Low |
| Jabbar et al., 2013 [[34](#_ENREF_34)] | Cross-sectional | 1 | 0 | 0 | 0 | 1 | 1 | 1 | NA | 1 | Medium |
| Jamal et al., 2002 [[35](#_ENREF_35)] | Cross-sectional | 1 | 0 | 0 | 1 | 1 | 1 | 1 | NA | 1 | Medium |
| Jeong et al., 2010 [[36](#_ENREF_36)] | Cross-sectional | 1 | 0 | 0 | 1 | 1 | 1 | 1 | NA | 1 | Medium |
| Kang et al., 2024 [[37](#_ENREF_37)] | Cohort | 1 | 1 | 1 | 1 | 1 | 1 | 1 | NA | 1 | Low |
| Karatas et al., 2018 [[38](#_ENREF_38)] | Cross-sectional | 0 | 0 | 0 | 0 | 1 | 1 | 1 | NA | 1 | High |
| Kart-Koseoglu et al., 2005 [[39](#_ENREF_39)] | Cross-sectional | 1 | 0 | 0 | 1 | 1 | 1 | 1 | NA | 1 | Medium |
| Khan et al., 2014 [[40](#_ENREF_40)] | Cross-sectional | 1 | 0 | 0 | 0 | 1 | 1 | 1 | NA | 1 | Medium |
| Kim et al., 2017 [[41](#_ENREF_41)] | Cohort | 1 | 1 | 1 | 0 | 1 | 1 | 1 | NA | 1 | Low |
| Kim et al., 2021 [[42](#_ENREF_42)] | Cross-sectional | 0 | 0 | 0 | 1 | 1 | 1 | 1 | NA | 1 | Medium |
| Kong et al., 2015 [[43](#_ENREF_43)] | Cross-sectional | 1 | 0 | 1 | 1 | 1 | 1 | 1 | NA | 1 | Low |
| Kocak et al., 2022 [[44](#_ENREF_44)] | Cohort | 0 | 0 | 0 | 1 | 1 | 1 | 1 | NA | 1 | Medium |
| Kratochvílová et al., 2019 [[45](#_ENREF_45)] | Cohort | 0 | 0 | 1 | 1 | 1 | 1 | 1 | NA | 1 | Medium |
| Lee et al., 2014 [[46](#_ENREF_46)] | Cross-sectional | 1 | 1 | 1 | 1 | 1 | 1 | 1 | NA | 1 | Low |
| Lee et al., 2020 [[47](#_ENREF_47)] | Cross-sectional | 1 | 0 | 0 | 1 | 1 | 1 | 1 | NA | 1 | Medium |
| Lee et al., 2023 [[48](#_ENREF_48)] | Cohort | 1 | 0 | 0 | 0 | 1 | 1 | 1 | NA | 1 | Medium |
| Lima et al., 2016 [[49](#_ENREF_49)] | Cross-sectional | 0 | 0 | 0 | 0 | 1 | 1 | 1 | NA | 1 | High |
| Lin et al., 2017 [[50](#_ENREF_50)] | Cross-sectional | 1 | 0 | 0 | 1 | 1 | 1 | 1 | NA | 1 | Medium |
| Lofdahl et al., 2020 [[51](#_ENREF_51)] | Cohort | 0 | 0 | 1 | 0 | 0 | 1 | 1 | NA | 0 | High |
| Lu et al., 2021 [[52](#_ENREF_52)] | Cross-sectional | 1 | 0 | 0 | 1 | 1 | 1 | 1 | NA | 1 | Medium |
| Malmgren et al., 2017 [[53](#_ENREF_53)] | Cohort | 0 | 1 | 1 | 1 | 1 | 1 | 1 | NA | 1 | Low |
| Mann et al., 2008 [[54](#_ENREF_54)] | Cross-sectional | 0 | 0 | 0 | 0 | 1 | 1 | 1 | NA | 1 | High |
| Marinho et al., 2016 [[55](#_ENREF_55)] | Cohort | 0 | 0 | 0 | 1 | 1 | 1 | 1 | NA | 1 | Medium |
| Mirfakhraee et al., 2012 [[56](#_ENREF_56)] | Cross-sectional | 0 | 0 | 0 | 1 | 1 | 1 | 1 | NA | 1 | Medium |
| Muxí et al., 2009 [[57](#_ENREF_57)] | Cohort | 1 | 0 | 0 | 0 | 1 | 1 | 1 | NA | 1 | Medium |
| Najar et al., 2017 [[58](#_ENREF_58)] | Cohort | 1 | 0 | 1 | 0 | 1 | 1 | 1 | NA | 1 | Medium |
| Nazzal et al., 2020 [[59](#_ENREF_59)] | Cross-sectional | 1 | 0 | 1 | 1 | 1 | 1 | 1 | NA | 1 | Low |
| Negri et al., 2004 [[60](#_ENREF_60)] | Cross-sectional | 1 | 0 | 0 | 0 | 1 | 1 | 1 | NA | 1 | Medium |
| Orlic et al., 2010 [[61](#_ENREF_61)] | Cross-sectional | 1 | 0 | 0 | 0 | 1 | 1 | 1 | NA | 1 | Medium |
| Ozkan et al., 2013 [[62](#_ENREF_62)] | Cross-sectional | 1 | 0 | 0 | 0 | 1 | 1 | 1 | NA | 1 | Medium |
| Park et al., 2017 [[63](#_ENREF_63)] | Cross-sectional | 1 | 0 | 1 | 1 | 1 | 1 | 1 | NA | 1 | Low |
| Patro et al., 2022 [[64](#_ENREF_64)] | Cross-sectional | 0 | 0 | 0 | 1 | 1 | 1 | 1 | NA | 1 | Medium |
| Petrauskiene et al., 2024 [[65](#_ENREF_65)] | RCT | 1 | 0 | 0 | 0 | 1 | 1 | 1 | NA | 1 | Medium |
| Polymeris et al., 2012 [[66](#_ENREF_66)] | Cross-sectional | 1 | 0 | 0 | 0 | 1 | 1 | 1 | NA | 1 | Medium |
| Prasad et al., 2019 [[67](#_ENREF_67)] | Cohort | 1 | 0 | 1 | 1 | 1 | 1 | 1 | NA | 1 | Low |
| Ray et al., 2017 [[68](#_ENREF_68)] | Cross-sectional | 0 | 0 | 0 | 1 | 1 | 1 | 1 | NA | 1 | Medium |
| Salam et al., 2018 [[69](#_ENREF_69)] | Cross-sectional | 0 | 0 | 0 | 1 | 1 | 1 | 1 | NA | 1 | Medium |
| Shin et al., 2014 [[70](#_ENREF_70)] | Cohort | 1 | 0 | 0 | 0 | 1 | 1 | 1 | NA | 1 | Medium |
| Sit et al., 2007 [[71](#_ENREF_71)] | Cross-sectional | 0 | 0 | 0 | 0 | 1 | 1 | 1 | NA | 1 | High |
| Slouma et al., 2020 [[72](#_ENREF_72)] | Cross-sectional | 1 | 0 | 0 | 1 | 1 | 1 | 1 | NA | 1 | Medium |
| Stavroulopoulos et al., 2008 [[73](#_ENREF_73)] | Cross-sectional | 1 | 0 | 0 | 0 | 1 | 1 | 1 | NA | 1 | Medium |
| Suh et al., 2023 [[74](#_ENREF_74)] | Cohort | 1 | 1 | 1 | 1 | 1 | 1 | 1 | NA | 1 | Low |
| Taal et al., 2003 [[75](#_ENREF_75)] | Cohort | 0 | 0 | 0 | 0 | 1 | 1 | 1 | NA | 1 | High |
| Tamadon et al., 2018 [[76](#_ENREF_76)] | Cross-sectional | 1 | 0 | 0 | 0 | 1 | 1 | 1 | NA | 1 | Medium |
| Tangvoraphonkchai et al., 2019 [[77](#_ENREF_77)] | Cohort | 1 | 0 | 0 | 1 | 1 | 1 | 1 | NA | 1 | Medium |
| Toussaint et al., 2009 [[78](#_ENREF_78)] | Cross-sectional | 0 | 0 | 0 | 1 | 1 | 1 | 1 | NA | 1 | Medium |
| Toussaint et al., 2009 [[79](#_ENREF_79)] | Cross-sectional | 0 | 0 | 0 | 1 | 1 | 1 | 1 | NA | 1 | Medium |
| Toussaint et al., 2010 [[80](#_ENREF_80)] | RCT | 0 | 0 | 0 | 1 | 1 | 1 | 1 | NA | 1 | Medium |
| Ureña et al., 2003 [[81](#_ENREF_81)] | Cross-sectional | 1 | 0 | 0 | 1 | 1 | 1 | 1 | NA | 1 | Medium |
| Valkovsky et al., 2015 [[82](#_ENREF_82)] | Cross-sectional | 1 | 0 | 0 | 1 | 1 | 1 | 1 | NA | 1 | Medium |
| Wang et al., 2016 [[83](#_ENREF_83)] | Cohort | 1 | 0 | 0 | 1 | 1 | 1 | 1 | NA | 1 | Medium |
| Wang et al., 2020 [[84](#_ENREF_84)] | Cross-sectional | 1 | 0 | 0 | 1 | 1 | 1 | 1 | NA | 1 | Medium |
| Wang et al., 2023 [[85](#_ENREF_85)] | Cross-sectional | 1 | 0 | 0 | 1 | 1 | 1 | 1 | NA | 1 | Medium |
| Wu et al., 2014 [[86](#_ENREF_86)] | Cross-sectional | 1 | 0 | 0 | 1 | 1 | 1 | 1 | NA | 1 | Medium |
| Wu et al., 2020 [[87](#_ENREF_87)] | Cross-sectional | 1 | 0 | 0 | 1 | 1 | 1 | 1 | NA | 1 | Medium |
| Yang et al., 2020 [[88](#_ENREF_88)] | Cohort | 1 | 0 | 0 | 1 | 1 | 1 | 1 | NA | 1 | Medium |
| Yap et al., 2017 [[89](#_ENREF_89)] | Cohort | 1 | 0 | 0 | 1 | 1 | 1 | 1 | NA | 1 | Medium |
| Yavropoulou et al., 2020 [[90](#_ENREF_90)] | Case-control | 0 | 0 | 0 | 1 | 1 | 1 | 1 | NA | 1 | Medium |
| Yoon et al, 2019 [[91](#_ENREF_91)] | Case-control | 1 | 0 | 0 | 1 | 1 | 1 | 1 | NA | 1 | Medium |
| Yucel et al., 2004 [[92](#_ENREF_92)] | Cross-sectional | 0 | 0 | 0 | 0 | 1 | 1 | 1 | NA | 1 | High |
| Zayour et al., 2004 [[93](#_ENREF_93)] | Cross-sectional | 1 | 0 | 0 | 1 | 1 | 1 | 1 | NA | 1 | Medium |
| Zheng et al., 2018 [[94](#_ENREF_94)] | Cross-sectional | 1 | 0 | 0 | 1 | 1 | 1 | 1 | NA | 1 | Medium |

High risk of bias: score 3 or 4, Medium risk of bias: score 5 or 6, Low risk of bias: score 7 or 8.

**eTable 3.** The baseline characteristics of the included studies.

| **Author/Year** | **Location** | **Continent** | **HDI tier** | **Type_Study** | **Sample size** | **Male sex** | **Female sex** | **CKD status** |
| --- | --- | --- | --- | --- | --- | --- | --- | --- |
| Afifi et al., 2019 [[1](#_ENREF_1)] | Egypt | Africa | 2 | Cross-sectional | 53 | 34 | 19 | HD |
| Aggarwal et al., 2013 [[2](#_ENREF_2)] | India | Asia | 3 | Cross-sectional | 75 | 51 | 24 | 3-5 (ND) |
| Aghighi et al., 2017 [[3](#_ENREF_3)] | Iran | Asia | 2 | Cross-sectional | 98 | 60 | 38 | HD |
| Akkupalli et al., 2013 [[4](#_ENREF_4)] | India | Asia | 3 | Cross-sectional | 20 | 10 | 10 | 3-5 (HD) |
| Amirkhanlou et al., 2021 [[5](#_ENREF_5)] | Iran | Asia | 2 | Cross-sectional | 200 | 80 | 120 | HD |
| Anwar et al., 2021 [[6](#_ENREF_6)] | Bangladesh | Asia | 3 | Cross-sectional | 41 | 23 | 18 | HD |
| Bezerra de Carvalho et al., 2019 [[7](#_ENREF_7)] | Brazil | America | 2 | Cohort | 415 | 66 | 349 | 3-5D (HD) |
| Binici et al., 2010 [[8](#_ENREF_8)] | Turkey | Asia | 1 | Cross-sectional | 124 | 67 | 57 | HD |
| Blomquist et al., 2016 [[9](#_ENREF_9)] | United states | America | 1 | Cross-sectional | 46 | 22 | 24 | HD |
| Bouksila et al., 2019 [[10](#_ENREF_10)] | Tunisia | Africa | 2 | Cross-sectional | 100 | 64 | 36 | HD |
| Brunerová et al., 2016 [[11](#_ENREF_11)] | Czech Republic | Europe | 1 | Cross-sectional | 59 | 43 | 16 | HD |
| Chen et al., 2019 [[12](#_ENREF_12)] | Taiwan | Asia | 2 | Cross-sectional | 164 | 90 | 74 | HD |
| Chi et al., 2022 [[13](#_ENREF_13)] | Taiwan | Asia | 2 | Cross-sectional | 80 |  |  | 3-5 (ND) |
| Chue et al., 2012 [[14](#_ENREF_14)] | United Kingdom | Europe | 1 | Cross-sectional | 120 | 65 | 55 | 3 (ND) |
| Dave et al., 2015 [[15](#_ENREF_15)] | Australia | Australia | 1 | Cross-sectional | 14 | 0 | 14 | 4-5 (HD) |
| Davenport et al., 2022 [[16](#_ENREF_16)] | United Kingdom | Europe | 1 | Cross-sectional | 573 | 331 | 242 | PD |
| Davenport et al., 2023 [[17](#_ENREF_17)] | United Kingdom | Europe | 1 | Cross-sectional | 734 | 420 | 314 | PD |
| Davina et al., 2017 [[18](#_ENREF_18)] | India | Asia | 3 | Cross-sectional | 32 | 32 | 0 | 4-5 (ND) |
| Demir et al., 2023 [[19](#_ENREF_19)] | Turkey | Asia | 1 | Cross-sectional | 23 | 14 | 9 | HD |
| Disthabanchong et al., 2014 [[20](#_ENREF_20)] | Thailand | Asia | 1 | Cohort | 83 | 38 | 45 | HD |
| Dogan et al., 2023 [[21](#_ENREF_21)] | Turkey | Asia | 1 | Cross-sectional | 46 | 27 | 19 | PD |
| Doumouchtsis et al., 2008 [[22](#_ENREF_22)] | Greece | Europe | 1 | Cross-sectional | 54 | 27 | 27 | HD |
| Ersoy et al., 2006 [[23](#_ENREF_23)] | Canada, Greece, Turkey | Multi | 1 | Cross-sectional | 292 | 162 | 130 | PD |
| Fidan et al., 2016 [[24](#_ENREF_24)] | Turkey | Asia | 1 | Cross-sectional | 83 | 41 | 42 | 3-5 (ND) |
| Filon et al., 2019 [[25](#_ENREF_25)] | Poland | Europe | 1 | Cross-sectional | 76 | 47 | 29 | HD |
| Gomez-Islas et al., 2020 [[26](#_ENREF_26)] | Mexico | America | 2 | Cohort | 218 | 0 | 218 | 3-5 (ND) |
| Grzegorzewska et al., 2008 [[27](#_ENREF_27)] | Poland | Europe | 1 | Cross-sectional | 30 | 12 | 18 | HD+PD |
| Huang et al., 2009 [[28](#_ENREF_28)] | Taiwan | Asia | 2 | Cross-sectional | 63 | 35 | 28 | HD |
| Huang et al., 2015 [[29](#_ENREF_29)] | Taiwan | Asia | 2 | Cross-sectional | 11 |  |  | 3-5 (ND) |
| Huang et al., 2024 [[30](#_ENREF_30)] | China | Asia | 2 | Cross-sectional | 87 | 57 | 30 | 5 (HD + PD + non-dialysis) |
| Hussain et al., 2023 [[31](#_ENREF_31)] | India | Asia | 3 | Cross-sectional | 50 | 35 | 15 | HD |
| Haarhaus et al., 2009 [[32](#_ENREF_32)] | Sweden | Europe | 1 | Cross-sectional | 44 |  |  | 3-5 (ND) |
| Haarhaus et al., 2009 [[32](#_ENREF_32)] | Sweden |  |  | Cross-sectional | 41 |  |  | 3-5 (ND) |
| Hyun et al., 2020 [[33](#_ENREF_33)] | South Korea | Asia | 4 | Cohort | 2128 | 824 | 1304 | ND |
| Jabbar et al., 2013 [[34](#_ENREF_34)] | India | Asia | 3 | Cross-sectional | 74 | 54 | 20 | 4-5 (ND) |
| Jamal et al., 2002 [[35](#_ENREF_35)] | Canada | America | 1 | Cross-sectional | 104 | 71 | 33 | HD |
| Jeong et al., 2010 [[36](#_ENREF_36)] | South Korea | Asia | 1 | Cross-sectional | 91 | 50 | 41 | PD |
| Kang et al., 2024 [[37](#_ENREF_37)] | South Korea | Asia | 1 | Cohort | 717 | 456 | 261 | 3-4 (ND) |
| Karatas et al., 2018 [[38](#_ENREF_38)] | Turkey | Asia | 1 | Cross-sectional | 57 | 30 | 27 | HD |
| Kart-Koseoglu et al., 2005 [[39](#_ENREF_39)] | Turkey | Asia | 1 | Cross-sectional | 76 |  |  | HD |
| Khan et al., 2014 [[40](#_ENREF_40)] | Saudi Arabia | Asia | 1 | Cross-sectional | 120 | 42 | 78 | HD |
| Kim et al., 2017 [[41](#_ENREF_41)] | South Korea | Asia | 1 | Cohort | 929 |  |  | 3-5 (ND) |
| Kim et al., 2021 [[42](#_ENREF_42)] | South Korea | Asia | 1 | Cross-sectional | 117 | 66 | 51 | 3-4 (ND) |
| Kong et al., 2015 [[43](#_ENREF_43)] | China | Asia | 2 | Cross-sectional | 299 | 232 | 67 | 3 (ND) |
| Kocak et al., 2022 [[44](#_ENREF_44)] | Turkey | Asia | 1 | Cohort | 81 | 39 | 42 | HD+PD |
| Kratochvílová et al., 2019 [[45](#_ENREF_45)] | Czech Republic | Europe | 1 | Cohort | 161 |  |  | 4-5D (HD+PD) |
| Kratochvílová et al., 2019 [[45](#_ENREF_45)] | Czech Republic |  |  | Cohort | 177 | 115 | 62 | 4-5D (HD+PD) |
| Lee et al., 2014 [[46](#_ENREF_46)] | South Korea | Asia | 1 | Cross-sectional | 571 | 276 | 295 | 3-4 (undefined) |
| Lee et al., 2020 [[47](#_ENREF_47)] | South Korea | Asia | 1 | Cross-sectional | 131 | 71 | 60 | HD |
| Lee et al., 2023 [[48](#_ENREF_48)] | South Korea | Asia | 1 | Cohort | 37 |  |  | HD+PD |
| Lima et al., 2016 [[49](#_ENREF_49)] | Brazil | America | 2 | Cross-sectional | 51 | 51 | 0 | 3-4 (ND) |
| Lin et al., 2017 [[50](#_ENREF_50)] | Taiwan | Asia | 2 | Cross-sectional | 48 | 19 | 29 | PD |
| Lofdahl et al., 2020 [[51](#_ENREF_51)] | Sweden | Europe | 1 | Cohort | 59 |  |  | 3-5 (ND) |
| Lofdahl et al., 2020 [[51](#_ENREF_51)] | Sweden |  |  | Cohort | 61 |  |  | 3-5 (ND) |
| Lu et al., 2021 [[52](#_ENREF_52)] | Taiwan | Asia | 2 | Cross-sectional | 80 | 41 | 39 | HD |
| Malmgren et al., 2017 [[53](#_ENREF_53)] | Sweden | Europe | 1 | Cohort | 1165 | 0 | 1165 | 3-5 (ND) |
| Mann et al., 2008 [[54](#_ENREF_54)] | United States | America | 1 | Cross-sectional | 30 | 12 | 18 | PD |
| Marinho et al., 2016 [[55](#_ENREF_55)] | Brazil | America | 2 | Cohort | 21 | 14 | 7 | HD |
| Mirfakhraee et al., 2012 [[56](#_ENREF_56)] | United states | America | 1 | Cross-sectional | 66 | 66 | 0 | HD |
| Muxí et al., 2009 [[57](#_ENREF_57)] | Spain | Europe | 1 | Cohort | 30 | 21 | 9 | HD |
| Najar et al., 2017 [[58](#_ENREF_58)] | India | Asia | 3 | Cohort | 151 | 98 | 53 | 3-5 (ND) |
| Nazzal et al., 2020 [[59](#_ENREF_59)] | Palestine | Asia | 2 | Cross-sectional | 194 | 114 | 80 | HD+PD |
| Negri et al., 2004 [[60](#_ENREF_60)] | Argentina | America | 1 | Cross-sectional | 65 | 20 | 45 | PD |
| Orlic et al., 2010 [[61](#_ENREF_61)] | Croatia | Europe | 1 | Cross-sectional | 134 | 72 | 62 | HD |
| Ozkan et al., 2013 [[62](#_ENREF_62)] | Turkey | Asia | 1 | Cross-sectional | 53 | 23 | 30 | PD |
| Park et al., 2017 [[63](#_ENREF_63)] | South Korea | Asia | 1 | Cross-sectional | 207 | 111 | 96 | HD+PD |
| Patro et al., 2022 [[64](#_ENREF_64)] | India | Asia | 3 | Cross-sectional | 50 | 26 | 24 | HD |
| Petrauskiene et al., 2024 [[65](#_ENREF_65)] | Sweden | Europe | 1 | RCT | 107 | 69 | 38 | 3-5 (ND) |
| Polymeris et al., 2012 [[66](#_ENREF_66)] | Greece | Europe | 1 | Cross-sectional | 37 | 19 | 18 | HD |
| Prasad et al., 2019 [[67](#_ENREF_67)] | Canada | America | 1 | Cohort | 374 | 228 | 146 | 3-5 (ND) |
| Ray et al., 2017 [[68](#_ENREF_68)] | India | Asia | 3 | Cross-sectional | 72 | 44 | 28 | 4-5 (ND) |
| Salam et al., 2018 [[69](#_ENREF_69)] | United Kingdom | Europe | 1 | Cross-sectional | 69 | 53 | 16 | 4-5 (HD + PD + non-dialysis) |
| Shin et al., 2014 [[70](#_ENREF_70)] | South Korea | Asia | 1 | Cohort | 89 | 30 | 59 | 3-5 (ND) |
| Sit et al., 2007 [[71](#_ENREF_71)] | Turkey | Asia | 1 | Cross-sectional | 70 | 37 | 33 | HD |
| Slouma et al., 2020 [[72](#_ENREF_72)] | Tunisia | Africa | 2 | Cross-sectional | 90 | 58 | 32 | HD |
| Stavroulopoulos et al., 2008 [[73](#_ENREF_73)] | United kingdom | Europe | 1 | Cross-sectional | 89 | 56 | 33 | 3-4 (ND) |
| Suh et al., 2023 [[74](#_ENREF_74)] | South Korea | Asia | 1 | Cohort | 1348 |  |  | 3-5 (ND) |
| Taal et al., 2003 [[75](#_ENREF_75)] | United Kingdom | Europe | 1 | Cohort | 77 | 40 | 37 | HD |
| Tamadon et al., 2018 [[76](#_ENREF_76)] | Iran | Asia | 2 | Cross-sectional | 77 | 39 | 38 | HD |
| Tangvoraphonkchai et al., 2019 [[77](#_ENREF_77)] | United Kingdom | Europe | 1 | Cohort | 125 | 71 | 54 | PD |
| Toussaint et al., 2009 [[78](#_ENREF_78)] | Australia | Australia | 1 | Cross-sectional | 45 | 29 | 16 | HD |
| Toussaint et al., 2009 [[79](#_ENREF_79)] | Australia | Australia | 1 | Cross-sectional | 40 | 28 | 12 | HD |
| Toussaint et al., 2010 [[80](#_ENREF_80)] | Australia | Australia | 1 | RCT | 50 | 33 | 17 | 3-4 (ND) |
| Ureña et al., 2003 [[81](#_ENREF_81)] | France | Europe | 1 | Cross-sectional | 70 | 44 | 26 | HD |
| Valkovsky et al., 2015 [[82](#_ENREF_82)] | Czech Republic | Europe | 1 | Cross-sectional | 82 | 54 | 28 | HD |
| Wang et al., 2016 [[83](#_ENREF_83)] | Taiwan | Asia | 2 | Cohort | 52 | 20 | 32 | PD |
| Wang et al., 2020 [[84](#_ENREF_84)] | Taiwan | Asia | 2 | Cross-sectional | 98 | 48 | 50 | HD |
| Wang et al., 2023 [[85](#_ENREF_85)] | China | Asia | 2 | Cross-sectional | 130 | 72 | 58 | HD |
| Wu et al., 2014 [[86](#_ENREF_86)] | China | Asia | 2 | Cross-sectional | 64 | 30 | 34 | HD |
| Wu et al., 2020 [[87](#_ENREF_87)] | Taiwan | Asia | 2 | Cross-sectional | 95 | 50 | 45 | HD |
| Yang et al., 2020 [[88](#_ENREF_88)] | China | Asia | 2 | Cohort | 72 | 40 | 32 | HD |
| Yap et al., 2017 [[89](#_ENREF_89)] | Australia | Australia | 1 | Cohort | 53 |  |  | HD |
| Yavropoulou et al., 2020 [[90](#_ENREF_90)] | Greece | Europe | 1 | Case-control | 30 | 22 | 8 | HD |
| Yoon et al, 2019 [[91](#_ENREF_91)] | South Korea | Asia | 1 | Case-control | 76 | 30 | 46 | HD+PD+transplant |
| Yucel et al., 2004 [[92](#_ENREF_92)] | Turkey | Asia | 1 | Cross-sectional | 76 | 44 | 32 | HD |
| Zayour et al., 2004 [[93](#_ENREF_93)] | Lebanon | Asia | 2 | Cross-sectional | 28 | 20 | 8 | HD |
| Zheng et al., 2018 [[94](#_ENREF_94)] | China | Asia | 2 | Cross-sectional | 125 | 73 | 52 | HD |

HDI: Human Development Index, CKD: Chronic kidney disease, HD: hemodialysis, PD: peritoneal dialysis, ND: Non-dialysis, HD + PD: hemodialysis + peritoneal dialysis

**eTable 4.** The osteopenic rate of the included studies.

| **Author/Year** | **General Ope** | **Lumbar Ope** | **Femoral neck Ope** | **Total hip Ope** | **Forearm Ope** | **Distal radius Ope** | **Male sex Ope** | **Female sex Ope** |
| --- | --- | --- | --- | --- | --- | --- | --- | --- |
| Afifi et al., 2019 [[1](#_ENREF_1)] | 30 | 15 | 27 |  |  |  |  |  |
| Aggarwal et al., 2013 [[2](#_ENREF_2)] |  | 25 |  |  |  |  |  |  |
| Aghighi et al., 2017 [[3](#_ENREF_3)] |  | 33 |  |  |  |  |  |  |
| Akkupalli et al., 2013 [[4](#_ENREF_4)] |  | 10 | 7 |  | 7 |  |  |  |
| Amirkhanlou et al., 2021 [[5](#_ENREF_5)] | 72 |  |  |  |  |  |  |  |
| Anwar et al., 2021 [[6](#_ENREF_6)] | 16 | 19 | 19 |  |  |  | 8 | 11 |
| Bezerra de Carvalho et al., 2019 [[7](#_ENREF_7)] |  | 141 |  | 132 |  |  |  |  |
| Binici et al., 2010 [[8](#_ENREF_8)] | 62 |  |  |  |  |  |  |  |
| Blomquist et al., 2016 [[9](#_ENREF_9)] |  | 11 | 15 | 18 |  |  |  |  |
| Bouksila et al., 2019 [[10](#_ENREF_10)] | 38 | 29 | 36 |  |  |  | 27 | 11 |
| Brunerová et al., 2016 [[11](#_ENREF_11)] | 21 |  |  |  |  |  |  |  |
| Chen et al., 2019 [[12](#_ENREF_12)] |  | 57 | 74 | 74 |  | 35 |  |  |
| Chi et al., 2022 [[13](#_ENREF_13)] | 35 |  |  |  |  |  |  |  |
| Chue et al., 2012 [[14](#_ENREF_14)] |  |  |  | 10 |  |  |  |  |
| Dave et al., 2015 [[15](#_ENREF_15)] |  |  | 2 |  |  |  |  | 2 |
| Davenport et al., 2022 [[16](#_ENREF_16)] |  |  | 344 |  |  |  |  |  |
| Davenport et al., 2023 [[17](#_ENREF_17)] |  | 244 | 416 |  |  |  | 135 | 109 |
| Davina et al., 2017 [[18](#_ENREF_18)] |  | 19 |  | 18 |  |  | 19 |  |
| Demir et al., 2023 [[19](#_ENREF_19)] | 12 |  |  |  |  |  |  |  |
| Disthabanchong et al., 2014 [[20](#_ENREF_20)] |  | 33 |  | 49 |  |  |  |  |
| Dogan et al., 2023 [[21](#_ENREF_21)] |  | 18 |  |  |  |  |  |  |
| Doumouchtsis et al., 2008 [[22](#_ENREF_22)] |  |  | 30 |  |  |  | 15 | 15 |
| Ersoy et al., 2006 [[23](#_ENREF_23)] |  | 106 |  |  |  |  |  |  |
| Fidan et al., 2016 [[24](#_ENREF_24)] |  | 43 | 47 |  |  |  | 24 | 23 |
| Filon et al., 2019 [[25](#_ENREF_25)] |  |  | 27 |  |  |  | 16 | 11 |
| Gomez-Islas et al., 2020 [[26](#_ENREF_26)] |  | 93 |  | 92 |  |  |  | 93 |
| Grzegorzewska et al., 2008 [[27](#_ENREF_27)] |  | 12 | 11 |  |  |  |  |  |
| Huang et al., 2009 [[28](#_ENREF_28)] | 43 | 24 | 40 | 28 |  |  | 14 | 10 |
| Huang et al., 2015 [[29](#_ENREF_29)] | 1 |  |  |  |  |  |  |  |
| Huang et al., 2024 [[30](#_ENREF_30)] |  | 17 | 37 |  |  |  |  |  |
| Hussain et al., 2023 [[31](#_ENREF_31)] |  | 20 | 24 |  |  | 21 |  |  |
| Haarhaus et al., 2009 [[32](#_ENREF_32)] |  |  |  | 17 |  |  |  |  |
| Haarhaus et al., 2009 [[32](#_ENREF_32)] |  |  |  |  |  | 6 |  |  |
| Hyun et al., 2020 [[33](#_ENREF_33)] | 705 |  |  |  |  |  | 389 | 316 |
| Jabbar et al., 2013 [[34](#_ENREF_34)] |  |  |  |  |  | 28 |  |  |
| Jamal et al., 2002 [[35](#_ENREF_35)] | 72 |  |  |  |  |  |  |  |
| Jeong et al., 2010 [[36](#_ENREF_36)] |  | 37 | 48 |  |  |  |  |  |
| Kang et al., 2024 [[37](#_ENREF_37)] | 129 |  |  |  |  |  |  |  |
| Karatas et al., 2018 [[38](#_ENREF_38)] | 21 |  |  |  |  |  |  |  |
| Kart-Koseoglu et al., 2005 [[39](#_ENREF_39)] |  | 25 |  |  | 23 |  |  |  |
| Khan et al., 2014 [[40](#_ENREF_40)] | 29 |  |  |  |  |  | 7 | 22 |
| Kim et al., 2017 [[41](#_ENREF_41)] |  | 342 | 464 | 309 |  |  |  |  |
| Kim et al., 2021 [[42](#_ENREF_42)] |  | 33 | 52 |  |  |  | 24 | 9 |
| Kong et al., 2015 [[43](#_ENREF_43)] |  | 85 |  |  |  |  |  |  |
| Kocak et al., 2022 [[44](#_ENREF_44)] |  | 30 | 42 | 33 |  |  |  |  |
| Kratochvílová et al., 2019 [[45](#_ENREF_45)] |  | 55 | 98 | 96 |  | 54 |  |  |
| Kratochvílová et al., 2019 [[45](#_ENREF_45)] |  |  |  |  |  |  | 65 | 35 |
| Lee et al., 2014 [[46](#_ENREF_46)] |  |  | 271 | 211 |  |  | 129 | 142 |
| Lee et al., 2020 [[47](#_ENREF_47)] | 58 |  |  |  |  |  | 43 | 15 |
| Lee et al., 2023 [[48](#_ENREF_48)] |  |  |  | 9 |  |  |  |  |
| Lima et al., 2016 [[49](#_ENREF_49)] | 23 |  |  |  |  |  | 23 |  |
| Lin et al., 2017 [[50](#_ENREF_50)] |  | 22 |  |  |  |  |  |  |
| Lofdahl et al., 2020 [[51](#_ENREF_51)] |  | 22 |  |  |  |  |  |  |
| Lofdahl et al., 2020 [[51](#_ENREF_51)] |  |  | 24 |  |  |  |  |  |
| Lu et al., 2021 [[52](#_ENREF_52)] |  | 19 |  |  |  |  | 4 | 15 |
| Malmgren et al., 2017 [[53](#_ENREF_53)] |  |  | 487 |  |  |  |  | 487 |
| Mann et al., 2008 [[54](#_ENREF_54)] |  | 5 | 12 | 7 |  |  |  |  |
| Marinho et al., 2016 [[55](#_ENREF_55)] | 8 |  |  |  |  |  |  |  |
| Mirfakhraee et al., 2012 [[56](#_ENREF_56)] |  | 24 | 36 | 33 |  | 12 | 24 |  |
| Muxí et al., 2009 [[57](#_ENREF_57)] |  |  |  |  | 8 |  |  |  |
| Najar et al., 2017 [[58](#_ENREF_58)] |  | 56 | 37 |  |  |  | 41 | 15 |
| Nazzal et al., 2020 [[59](#_ENREF_59)] | 78 | 74 |  | 81 |  |  | 38 | 36 |
| Negri et al., 2004 [[60](#_ENREF_60)] |  | 29 | 37 |  |  |  | 9 | 20 |
| Orlic et al., 2010 [[61](#_ENREF_61)] |  | 45 | 68 | 60 | 51 | 52 | 24 | 21 |
| Ozkan et al., 2013 [[62](#_ENREF_62)] |  |  | 16 |  |  |  | 6 | 10 |
| Park et al., 2017 [[63](#_ENREF_63)] |  |  | 87 |  |  |  |  |  |
| Patro et al., 2022 [[64](#_ENREF_64)] | 10 |  |  |  |  |  |  |  |
| Petrauskiene et al., 2024 [[65](#_ENREF_65)] |  | 32 |  |  |  |  |  |  |
| Polymeris et al., 2012 [[66](#_ENREF_66)] |  | 12 | 19 |  |  |  |  |  |
| Prasad et al., 2019 [[67](#_ENREF_67)] |  |  |  | 162 |  |  | 93 | 69 |
| Ray et al., 2017 [[68](#_ENREF_68)] |  |  |  |  |  | 30 |  |  |
| Salam et al., 2018 [[69](#_ENREF_69)] | 41 |  |  |  |  |  |  |  |
| Shin et al., 2014 [[70](#_ENREF_70)] | 6 |  |  |  |  |  | 1 | 5 |
| Sit et al., 2007 [[71](#_ENREF_71)] |  | 25 | 38 |  |  |  | 13 | 12 |
| Slouma et al., 2020 [[72](#_ENREF_72)] | 42 | 30 | 45 | 40 |  |  |  |  |
| Stavroulopoulos et al., 2008 [[73](#_ENREF_73)] | 31 |  |  | 25 | 14 |  |  |  |
| Suh et al., 2023 [[74](#_ENREF_74)] |  |  | 388 |  |  |  |  |  |
| Taal et al., 2003 [[75](#_ENREF_75)] |  |  |  | 34 |  |  |  |  |
| Tamadon et al., 2018 [[76](#_ENREF_76)] |  | 33 | 35 | 35 |  |  |  |  |
| Tangvoraphonkchai et al., 2019 [[77](#_ENREF_77)] |  | 59 | 99 |  |  |  |  |  |
| Toussaint et al., 2009 [[78](#_ENREF_78)] |  | 17 | 29 |  |  |  |  |  |
| Toussaint et al., 2009 [[79](#_ENREF_79)] |  | 8 |  |  |  |  |  |  |
| Toussaint et al., 2010 [[80](#_ENREF_80)] |  | 8 | 20 |  |  |  |  |  |
| Ureña et al., 2003 [[81](#_ENREF_81)] |  | 42 | 30 |  |  |  |  |  |
| Valkovsky et al., 2015 [[82](#_ENREF_82)] |  | 27 | 44 | 37 |  |  | 15 | 12 |
| Wang et al., 2016 [[83](#_ENREF_83)] |  | 23 |  |  |  |  |  |  |
| Wang et al., 2020 [[84](#_ENREF_84)] |  | 25 |  |  |  |  | 9 | 16 |
| Wang et al., 2023 [[85](#_ENREF_85)] | 44 |  |  |  |  |  | 24 | 20 |
| Wu et al., 2014 [[86](#_ENREF_86)] |  |  | 24 |  |  |  | 10 | 14 |
| Wu et al., 2020 [[87](#_ENREF_87)] |  | 27 |  |  |  |  |  |  |
| Yang et al., 2020 [[88](#_ENREF_88)] | 38 |  |  |  |  |  |  |  |
| Yap et al., 2017 [[89](#_ENREF_89)] |  |  | 30 |  |  |  |  |  |
| Yavropoulou et al., 2020 [[90](#_ENREF_90)] | 11 |  |  |  |  |  |  |  |
| Yoon et al, 2019 [[91](#_ENREF_91)] | 33 |  |  |  |  |  |  |  |
| Yucel et al., 2004 [[92](#_ENREF_92)] |  | 26 | 31 |  |  | 23 |  |  |
| Zayour et al., 2004 [[93](#_ENREF_93)] | 8 |  |  |  |  |  |  |  |
| Zheng et al., 2018 [[94](#_ENREF_94)] |  | 36 | 41 |  |  |  | 21 | 15 |

Ope: Osteopenia

**eFigures**

**eFigure 1.** Funnel plot of publication bias of primary studies with osteopenia for the femoral neck region.

**eFigure 2.** Funnel plot of publication bias of primary studies with osteopenia for the lumbar spine region.

**eFigure 3.** The forest plot of the prevalence of total hip osteopenia by the primary studies in adults with chronic kidney disease, and the overall estimate (95% CI).

**eFigure 4.** Funnel plot of publication bias of primary studies with osteopenia for the total hip region.

**eFigure 5.** The forest plot of the prevalence of general osteopenia by the primary studies in adults with chronic kidney disease, and the overall estimate (95% CI).

**eFigure 6.** Funnel plot of publication bias of primary studies with general osteopenia.

**eFigure 7.** The forest plot of the prevalence of male osteopenia by the primary studies in adults with chronic kidney disease, and the overall estimate (95% CI).

**eFigure 8.** Funnel plot of publication bias of primary studies with male osteopenia.

**eFigure 9.** The forest plot of the prevalence of female osteopenia by the primary studies in adults with chronic kidney disease, and the overall estimate (95% CI).

**eFigure 10.** Funnel plot of publication bias of primary studies with female osteopenia.

**References**

1. Afifi, W.M., et al., *Musculoskeletal manifestations in end-stage renal disease patients on hemodialysis and relation to parathyroid dysfunction.* Saudi Journal of Kidney Diseases and Transplantation, 2019. **30**(1): p. 68-82.

2. Aggarwal, H., et al., *Bone mineral density in patients with predialysis chronic kidney disease.* Renal failure, 2013. **35**(8): p. 1105-1111.

3. Aghighi, M., et al., *Factors associated with lumbar and femoral bone mineral density in kidney transplants candidates.* Iranian journal of kidney diseases, 2017. **11**(5): p. 379.

4. Akkupalli, L., et al., *Bone Mineral Density In Chronic Kidney Disease Patients.* Int J Biol Med Res, 2013. **4**(1): p. 2870-2874.

5. Amirkhanlou, S., et al., *Assessment of bone mineral density in patients undergoing hemodialysis; An iranian population-based study.* Archives of Iranian Medicine, 2021. **24**(8): p. 599-606.

6. Anwar, S.I., et al., *Bone Mineral Density to Identify Osteopenia and Osteoporosis in Patients of CKD on Maintenance Hemodialysis.* Journal of Dhaka Medical College, 2020. **29**(1): p. 3-11.

7. Bezerra de Carvalho, K., et al., *Chronic kidney disease is associated with low BMD at the hip but not at the spine.* Osteoporosis International, 2019. **30**: p. 1015-1023.

8. Binici, D.N. and N. Gunes, *Risk factors leading to reduced bone mineral density in hemodialysis patients with metabolic syndrome.* Renal failure, 2010. **32**(4): p. 469-474.

9. Blomquist, G.A., et al., *Diagnosis of low bone mass in CKD-5D patients.* Clinical Nephrology, 2015. **85**(2): p. 77.

10. Bouksila, M., et al., *Correlation of Fgf23 and balp with bone mineral density in hemodialysis patients.* Journal of medical biochemistry, 2019. **38**(4): p. 418.

11. Brunerová, L., et al., *Osteoporosis and impaired trabecular bone score in hemodialysis patients.* Kidney and Blood Pressure Research, 2016. **41**(3): p. 345-354.

12. Chen, S.-C., et al., *Associations among geriatric nutrition risk index, bone mineral density, body composition and handgrip strength in patients receiving hemodialysis.* Nutrition, 2019. **65**: p. 6-12.

13. Chi, P.-J., et al., *Serum osteocalcin concentration as an independent biomarker of osteoporosis in patients with chronic kidney disease.* Clinical Nephrology, 2022. **98**(1): p. 1.

14. Chue, C.D., et al., *Aortic calcification and femoral bone density are independently associated with left ventricular mass in patients with chronic kidney disease.* PloS one, 2012. **7**(6): p. e39241.

15. Dave, V., et al., *Hypocalcemia post denosumab in patients with chronic kidney disease stage 4-5.* American journal of nephrology, 2015. **41**(2): p. 129-137.

16. Davenport, A., *Frailty, appendicular lean mass, osteoporosis and osteosarcopenia in peritoneal dialysis patients.* Journal of Nephrology, 2022. **35**(9): p. 2333-2340.

17. Davenport, A., *Differences in prevalence of reduced and low bone mineral density between lumbar spine and femoral neck in peritoneal dialysis patients using dual-energy X-ray absorptiometry (DXA).* Peritoneal Dialysis International, 2023. **43**(4): p. 334-338.

18. Davina, J.J., et al., *Assessment of bone turnover markers to predict mineral and bone disorder in men with pre-dialysis non-diabetic chronic kidney disease.* Clinica Chimica Acta, 2017. **469**: p. 195-200.

19. Demir, C., et al., *Serum irisin levels and osteoporosis in patients with advanced chronic kidney disease and renal transplant recipients.* International Urology and Nephrology, 2023. **55**(7): p. 1821-1828.

20. Disthabanchong, S., et al., *Low hip bone mineral density predicts mortality in maintenance hemodialysis patients: a five-year follow-up study.* Blood purification, 2014. **37**(1): p. 33-38.

21. Doğan, A.G., et al., *The relationship between serum pro-B type natriuretic peptide level and bone mineral density in peritoneal dialysis patients.* Medicine, 2023. **102**(38): p. e34666.

22. Doumouchtsis, K.K., et al., *Associations between osteoprotegerin and femoral neck BMD in hemodialysis patients.* Journal of bone and mineral metabolism, 2008. **26**: p. 66-72.

23. Ersoy, F.F., et al., *Bone mineral density and its correlation with clinical and laboratory factors in chronic peritoneal dialysis patients.* Journal of bone and mineral metabolism, 2006. **24**: p. 79-86.

24. Fidan, N., et al., *Bone mineral density and biochemical markers of bone metabolism in predialysis patients with chronic kidney disease.* Journal of Investigative Medicine, 2016. **64**(4): p. 861-866.

25. Fiłon, T., et al., *Muscle strength and bone mass density in haemodialysis patients.* Physiotherapy Quarterly, 2019. **27**(1): p. 39-45.

26. Gómez-Islas, V.E., et al., *Evaluation of bone densitometry by dual-energy x-ray absorptiometry as a fracture prediction tool in women with chronic kidney disease.* Bone Reports, 2020. **13**: p. 100298.

27. Grzegorzewska, A.E. and M. Mlot-Michalska, *Coffee consumption and bone mineral density in dialysis patients.* Advances in Peritoneal Dialysis, 2008. **24**: p. 84-89.

28. Huang, G.-S., et al., *Factors associated with low bone mass in the hemodialysis patients–a cross-sectional correlation study.* BMC musculoskeletal disorders, 2009. **10**: p. 1-10.

29. Huang, J.-H., F.-C. Cheng, and H.-C. Wu, *Low magnesium exacerbates osteoporosis in chronic kidney disease patients with diabetes.* International journal of endocrinology, 2015. **2015**(1): p. 380247.

30. Huang, T., et al., *The relationship between serum fibroblast growth factor 23 and klotho protein and low bone mineral density in middle-aged and elderly patients with end-stage renal disease.* Hormone and Metabolic Research, 2024. **56**(02): p. 142-149.

31. Hussain, I., et al., *Correlation of FGF-23 with biochemical markers and bone density in chronic kidney disease-bone mineral density disorder.* Cureus, 2023. **15**(1).

32. Haarhaus, M., et al., *Clinical significance of bone alkaline phosphatase isoforms, including the novel B1x isoform, in mild to moderate chronic kidney disease.* Nephrology Dialysis Transplantation, 2009. **24**(11): p. 3382-3389.

33. Hyun, Y., et al., *Risk factors and renal outcomes of low bone mineral density in patients with non-dialysis chronic kidney disease.* Osteoporosis International, 2020. **31**: p. 2373-2382.

34. Jabbar, Z., et al., *Noninvasive assessment of bone health in Indian patients with chronic kidney disease.* Indian journal of nephrology, 2013. **23**(3): p. 161-167.

35. Jamal, S.A., et al., *Bone density and heel ultrasound testing do not identify patients with dialysis-dependent renal failure who have had fractures.* American journal of kidney diseases, 2002. **39**(4): p. 843-849.

36. Jeong, J., et al., *Nutritional markers, not markers of bone turnover, are related predictors of bone mineral density in chronic peritoneal dialysis patients.* Clinical nephrology, 2010. **74**(5): p. 336-342.

37. Kang, D.H., et al., *Kidney function and bone mineral density in chronic kidney disease patients.* Clinical Kidney Journal, 2024. **17**(9): p. sfae248.

38. Karataş, A. and E. Çanakçı, *Factors Affecting Bone Mineral Density in Hemodialysis Patients.* Middle Black Sea Journal of Health Science, 2018. **4**(3): p. 26-33.

39. Kart-Köseoglu, H., et al., *Osteoarthritis in hemodialysis patients: relationships with bone mineral density and other clinical and laboratory parameters.* Rheumatology international, 2005. **25**: p. 270-275.

40. Khan, M.I., et al., *Mean bone mineral density and frequency of occurrence of osteopenia and osteoporosis in patients on hemodialysis: a single-center study.* Saudi Journal of Kidney Diseases and Transplantation, 2014. **25**(1): p. 38-43.

41. Kim, C.S., et al., *Chronic kidney disease-mineral bone disorder in Korean patients: a report from the Korean cohort study for outcomes in patients with chronic kidney disease (KNOW-CKD).* Journal of Korean medical science, 2017. **32**(2): p. 240-248.

42. Kim, K., et al., *Is dual-energy absorptiometry accurate in the assessment of bone status of patients with chronic kidney disease?* Osteoporosis International, 2021. **32**: p. 1859-1868.

43. Kong, X., et al., *Relationship between mild-to-moderate chronic kidney disease and decreased bone mineral density in Chinese adult population.* International urology and nephrology, 2015. **47**: p. 1547-1553.

44. Kocak, S.Y. and A. Ozdemir, *Comparison of bone mineral density and biochemical factors in hemodialysis and peritoneal dialysis patients.* Clinical Nephrology, 2022. **98**(3): p. 115.

45. Kratochvílová, S., et al., *Retrospective Analysis of Bone Metabolism in Patients on Waiting List for Simultaneous Pancreas‐Kidney Transplantation.* Journal of Diabetes Research, 2019. **2019**(1): p. 5143021.

46. Lee, Y.-h., et al., *The combination of vitamin D deficiency and mild to moderate chronic kidney disease is associated with low bone mineral density and deteriorated femoral microarchitecture: results from the KNHANES 2008–2011.* The Journal of Clinical Endocrinology & Metabolism, 2014. **99**(10): p. 3879-3888.

47. Lee, H., et al., *Association of nutritional status with osteoporosis, sarcopenia, and cognitive impairment in patients on hemodialysis.* Asia Pacific Journal of Clinical Nutrition, 2020. **29**(4): p. 712-723.

48. Lee, S.-M., et al., *Hip arthroplasty for patients with chronic renal failure on dialysis.* Scientific Reports, 2023. **13**(1): p. 3311.

49. Lima, G.A.C., et al., *Bone density is directly associated with glomerular filtration and metabolic acidosis but do not predict fragility fractures in men with moderate chronic kidney disease.* Journal of Clinical Densitometry, 2016. **19**(2): p. 146-153.

50. Lin, Y.L., et al., *Triceps skinfold thickness is associated with lumbar bone mineral density in peritoneal dialysis patients.* Therapeutic Apheresis and Dialysis, 2017. **21**(1): p. 102-107.

51. Löfdahl, E., C. Haggård, and G. Rådegran, *Bone mineral density in relation to chronic kidney disease after heart transplantation: a retrospective single-center study at Skåne University Hospital in Lund 1988–2016.* Transplantation direct, 2020. **6**(3): p. e537.

52. Lu, C.-W., et al., *Serum irisin level is positively associated with bone mineral density in patients on maintenance hemodialysis.* International journal of endocrinology, 2021. **2021**(1): p. 8890042.

53. Malmgren, L., et al., *Reduced kidney function is associated with BMD, bone loss and markers of mineral homeostasis in older women: a 10-year longitudinal study.* Osteoporosis International, 2017. **28**: p. 3463-3473.

54. Mann, M.L., et al., *The effect of peritoneal dialysate on DXA bone densitometry results in patients with end-stage renal disease.* Journal of Clinical Densitometry, 2008. **11**(4): p. 532-536.

55. Marinho, S.M., et al., *Exercise training alters the bone mineral density of hemodialysis patients.* The Journal of Strength & Conditioning Research, 2016. **30**(10): p. 2918-2923.

56. Mirfakhraee, S., et al., *Risk factors for diminished bone mineral density among male hemodialysis patients—a cross-sectional study.* Archives of osteoporosis, 2012. **7**: p. 283-290.

57. Muxí, A., et al., *Arteriovenous fistula affects bone mineral density measurements in end-stage renal failure patients.* Clinical Journal of the American Society of Nephrology, 2009. **4**(9): p. 1494-1499.

58. Najar, S.M., M.M. Mir, and M. Muzamil, *Prevalence of osteoporosis in patients with chronic kidney disease (stages 3-5) in comparison with age-and sex-matched controls: A study from Kashmir Valley Tertiary Care Center.* Saudi Journal of Kidney Diseases and Transplantation, 2017. **28**(3): p. 538-544.

59. Nazzal, Z., et al., *Bone mineral density in Palestinian patients with end-stage renal disease and the related clinical and biochemical factors: cross-sectional study.* PLoS One, 2020. **15**(11): p. e0241201.

60. Negri, A.L., et al., *Bone mineral density: serum markers of bone turnover and their relationships in peritoneal dialysis.* Peritoneal dialysis international, 2004. **24**(2): p. 163-168.

61. Orlic, L., et al., *Bone mineral densitometry in patients on hemodialysis: difference between genders and what to measure.* Renal failure, 2010. **32**(3): p. 300-308.

62. Özkan, O., et al., *The factors effective on bone mineral density in peritoneal dialysis patients.* European Journal of General Medicine, 2013. **10**(4): p. 219-225.

63. Park, W., et al. *Progression of osteoporosis after kidney transplantation in patients with end-stage renal disease*. in *Transplantation proceedings*. 2017. Elsevier.

64. Patro, S.K., N. Pawar, and D. Biswas, *A study to predict fracture risk using bone mineral density and FRAX score in patients on chronic maintenance haemodialysis.* Journal of Family Medicine and Primary Care, 2022. **11**(1): p. 170-175.

65. Petrauskiene, V., et al., *Bone mineral density after exercise training in patients with chronic kidney disease stages 3 to 5: a sub-study of RENEXC—a randomized controlled trial.* Clinical Kidney Journal, 2024. **17**(1): p. sfad287.

66. Polymeris, A., K. Doumouchtsis, and E. Grapsa, *Bone mineral density and bone metabolism in hemodialysis patients. Correlation with PTH, 25OHD3 and leptin.* Nefrología (English Edition), 2012. **32**(1): p. 73-78.

67. Prasad, B., et al., *Association of bone mineral density with fractures across the spectrum of chronic kidney disease: the Regina CKD-MBD study.* Canadian journal of kidney health and disease, 2019. **6**: p. 2054358119870539.

68. Ray, S., et al., *Profile of chronic kidney disease related-mineral bone disorders in newly diagnosed advanced predialysis diabetic kidney disease patients: A hospital based cross-sectional study.* Diabetes & Metabolic Syndrome: Clinical Research & Reviews, 2017. **11**: p. S931-S937.

69. Salam, S., et al., *Diagnostic accuracy of biomarkers and imaging for bone turnover in renal osteodystrophy.* Journal of the American Society of Nephrology, 2018. **29**(5): p. 1557-1565.

70. Shin, J.-h., S.H. Kim, and S.-H. Yu, *Metabolic syndrome and chronic kidney disease as risk factors of osteoporosis.* Clinical Nephrology, 2014. **81**(1): p. 1-8.

71. Sit, D., et al., *Relationship between bone mineral density and biochemical markers of bone turnover in hemodialysis patients.* Advances in therapy, 2007. **24**: p. 987-995.

72. Slouma, M., et al., *Mineral bone disorder and osteoporosis in hemodialysis patients.* Advances in Rheumatology, 2020. **60**: p. 1-7.

73. Stavroulopoulos, A., et al., *Relationship between vitamin D status, parathyroid hormone levels and bone mineral density in patients with chronic kidney disease stages 3 and 4.* Nephrology, 2008. **13**(1): p. 63-67.

74. Suh, S.H., et al., *Bone mineral density and all-cause mortality in patients with nondialysis chronic kidney disease: results from KNOW-CKD study.* Journal of clinical medicine, 2023. **12**(5): p. 1850.

75. Taal, M.W., et al., *Total hip bone mass predicts survival in chronic hemodialysis patients.* Kidney international, 2003. **63**(3): p. 1116-1120.

76. Tamadon, M.R., J. Moghimi, and V. Semnani, *Bone mineral density and bone metabolism biochemical markers in patients with chronic kidney disease at the hemodialysis treatment.* Journal of Parathyroid Disease, 2017. **6**(2): p. 50-56.

77. Tangvoraphonkchai, K. and A. Davenport, *Aortic pulse wave velocity is greater in peritoneal dialysis patients with lower dual energy X-ray absorptiometry (DXA) femoral neck bone mineral density.* Journal of Nephrology, 2019. **32**: p. 471-476.

78. Toussaint, N.D., et al., *Determination and validation of aortic calcification measurement from lateral bone densitometry in dialysis patients.* Clinical Journal of the American Society of Nephrology, 2009. **4**(1): p. 119-127.

79. Toussaint, N.D., et al., *Relationship between vascular calcification, arterial stiffness and bone mineral density in a cross‐sectional study of prevalent Australian haemodialysis patients.* Nephrology, 2009. **14**(1): p. 105-112.

80. Toussaint, N.D., et al., *Effect of alendronate on vascular calcification in CKD stages 3 and 4: a pilot randomized controlled trial.* American Journal of Kidney Diseases, 2010. **56**(1): p. 57-68.

81. Urena, P., et al., *Bone mineral density, biochemical markers and skeletal fractures in haemodialysis patients.* Nephrology Dialysis Transplantation, 2003. **18**(11): p. 2325-2331.

82. Valkovsky, I., et al., *Evaluation of biochemical markers and bone mineral density in patients with chronic kidney disease stage 5D at the start of hemodialysis treatment.* Biomedical Papers, 2015. **159**(1): p. 93-99.

83. Wang, C.-H., et al., *Inverse relationship of bone mineral density and serum level of N-terminal pro-B-type natriuretic peptide in peritoneal dialysis patients.* Tzu Chi Medical Journal, 2016. **28**(2): p. 68-72.

84. Wang, C.-H., et al., *Increased serum leptin level predicts bone mineral density in hemodialysis patients.* International Journal of Endocrinology, 2020. **2020**(1): p. 8451751.

85. Wang, Y., et al., *Interrelationships between sarcopenia, bone turnover markers and low bone mineral density in patients on hemodialysis.* Renal Failure, 2023. **45**(1): p. 2200846.

86. Wu, Q., et al., *Effect of Serum Fibroblast Growth Factor‐23, Matrix G la Protein and F etuin‐A in Predicting Osteoporosis in Maintenance Hemodialysis Patients.* Therapeutic Apheresis and Dialysis, 2014. **18**(5): p. 427-433.

87. Wu, Y.-T., et al., *Lower serum fibroblast growth factor 21 levels are associated with normal lumbar spine bone mineral density in hemodialysis patients.* International journal of environmental research and public health, 2020. **17**(6): p. 1938.

88. Yang, S., et al., *Effect of long‐term use of unfractionated or low‐molecular‐weight heparin on bone mineral density in maintenance hemodialysis patients.* Hemodialysis International, 2020. **24**(3): p. 374-382.

89. Yap, N., et al., *Femoral neck X-ray absorptiometry parameters and peripheral quantitative computer tomography tibial cortical density predict survival in dialysis patients.* Nephron, 2017. **136**(3): p. 183-192.

90. Yavropoulou, M.P., et al., *Expression of circulating MicroRNAs linked to bone metabolism in chronic kidney disease-mineral and bone disorder.* Biomedicines, 2020. **8**(12): p. 601.

91. Yoon, H.E., et al., *Factors associated with low trabecular bone scores in patients with end-stage kidney disease.* Journal of Bone and Mineral Metabolism, 2019. **37**: p. 475-483.

92. Yücel, A.E., et al., *Bone mineral density in patients on maintenance hemodialysis and effect of chronic hepatitis C virus infection.* Renal failure, 2004. **26**(2): p. 159-164.

93. Zayour, D., et al. *Predictors of bone mineral density in patients on hemodialysis*. in *Transplantation proceedings*. 2004. Elsevier.

94. Zheng, S., et al., *Correlation of serum levels of fibroblast growth factor 23 and Klotho protein levels with bone mineral density in maintenance hemodialysis patients.* European Journal of Medical Research, 2018. **23**: p. 1-7.
